# Molecular and Functional Divergence of Zebrafish Sox Paralogs Controlling Endoderm Formation and Left–Right Patterning

**DOI:** 10.1093/gbe/evaf213

**Published:** 2025-11-11

**Authors:** Simaran Johal, Randa Elsayed, Dongfeng Wang, Conor D Talbot, Roberto Feuda, Kristen A Panfilio, Andrew C Nelson

**Affiliations:** School of Life Sciences, Gibbet Hill Campus, University of Warwick, Coventry CV4 7AL, UK; Warwick Medical School, Gibbet Hill Campus, University of Warwick, Coventry CV4 7AL, UK; Neurogenetics Group, University of Leicester, Leicester, UK; Department of Genetics, Genomics and Cancer Sciences, University of Leicester, Leicester, UK; School of Life Sciences, Gibbet Hill Campus, University of Warwick, Coventry CV4 7AL, UK; Neurogenetics Group, University of Leicester, Leicester, UK; Department of Genetics, Genomics and Cancer Sciences, University of Leicester, Leicester, UK; School of Life Sciences, Gibbet Hill Campus, University of Warwick, Coventry CV4 7AL, UK; Department of Molecular Genetics, Institute of Biology, University of Hohenheim, Stuttgart 70599, Germany; Institute for Zoology: Developmental Biology, University of Cologne, Cologne 50674, Germany; School of Life Sciences, Gibbet Hill Campus, University of Warwick, Coventry CV4 7AL, UK

**Keywords:** endoderm, zebrafish, left–right patterning, gene regulation

## Abstract

Endoderm, one of three primary germ layers of vertebrate embryos, makes major contributions to the respiratory and gastrointestinal tracts and associated organs, including the liver and pancreas. In mammals, transcription factor (TF) *SOX17* is vital for endoderm organ formation and can induce endoderm progenitor identity. Duplication of ancestral *sox17* before or during the early evolution of ray-finned fishes produced paralogs *sox32* and *sox17* in zebrafish. Sox32 is required for specification of endoderm and progenitors of the left–right (LR) organizer (Kupffer's Vesicle, KV), with Sox17 a downstream target of Sox32 implicated in further KV development. Phenotypic evidence, therefore, suggests functional similarities between zebrafish Sox32 and Sox17 and mammalian SOX17. Here, we directly compare these orthologs and paralogs, using the early zebrafish embryo as a biological platform for functional testing. Our results indicate that, unlike Sox32, human SOX17 cannot induce endoderm specification in zebrafish. Furthermore, using hybrid protein functional analyses, we show that Sox32 specificity for the endoderm gene regulatory network is linked to evolutionary divergence in its DNA-binding High Mobility Group domain from its paralog Sox17. Additionally, changes in the C-terminal regions of Sox32 and Sox17 underpin their differing target specificities. Finally, we establish that specific conserved peptides in the Sox17 C-terminal domain are essential for its role in establishing correct organ asymmetry. Overall, our results illuminate the molecular basis for functional divergence of Sox32 and Sox17 in vertebrate endoderm development and LR patterning, and reveal that alterations in specific domains of both TFs at different points during the evolution of fish are critical to their distinct and essential functions.

Significance statement The Sox family transcription factor (TF) *Sox17* has key roles in endoderm induction and left–right patterning in vertebrate embryos but underwent duplication, leading to two TFs (Sox32 and Sox17) in the *Actinopterygii*, with distinct functions in these processes that are not well understood at the molecular level. We find that the evolutionary divergence of the DNA-binding High Mobility Group domain and C-terminal helical domains of Sox32 and Sox17 is critical for the ability of Sox32 to engage the zebrafish endoderm gene regulatory network, and to distinguish zebrafish Sox32 and Sox17 function. Our findings illuminate the molecular basis for functional divergence of these two factors via subfunctionalization through accelerated sequence divergence that enabled one paralog to act as the endodermal “master regulator,” upstream of its paralog, while neofunctionalization has additionally led to novel roles in later development for the downstream paralog.

## Introduction

Sox family transcription factors (TFs) are evolutionarily conserved proteins with roles in cell fate decisions in a range of developmental processes (Sarkar and Hochedlinger 2013). Mutations in Sox factors have been linked to an array of developmental defects and congenital diseases in humans (Angelozzi and Lefebvre 2019). Sox TFs belong to the superfamily of High Mobility Group (HMG) domain containing TFs. Historically, membership of the Sox family has been dictated by having >50% conservation of the DNA-binding HMG domain with mouse SRY—the founding Sox TF (Gubbay et al. 1990; Sinclair et al. 1990; Pevny and Lovell-Badge 1997). Sox factors have been further characterized into subfamilies, whereby members of a subfamily typically exhibit 75% to 80% homology within their HMG domain (Bowles et al. 2000; Wegner 2010). One subfamily is SoxF, which in mammals consists of SOX7, SOX17, and SOX18. In ray-finned fish, SoxF is expanded to include Sox32. How has this additional TF been functionally integrated into SoxF developmental gene regulatory networks (GRNs)?

Most duplicated *sox* genes in the teleost lineage arose through a whole genome duplication event that occurred 226 to 316 million years ago (Voldoire et al. 2017). An exception to this is *sox32*, which is hypothesized to have emerged through tandem duplication of *Sox17* (Voldoire et al. 2017). While we and others have speculated that zebrafish Sox32 may function in similar processes to mammalian SOX17 (Lilly et al. 2017; Figiel et al. 2021), this has not been explored at the molecular level. Furthermore, it is unknown whether functions of the ancestral gene have been split among the two paralogs (subfunctionalization) or whether they have functionally diverged post-duplication and acquired new roles (neofunctionalization) (Force et al. 1999). Although *sox17* has some unique expression domains in later embryogenesis (Chung et al. 2011), *sox17* and *sox32* are substantially co-expressed both spatially and temporally in early development, including in progenitors of the endoderm and left–right (LR) organizer during gastrulation (Alexander and Stainier 1999; Kikuchi et al. 2001). We thus consider that functional evolution (sub- or neofunctionalization) in early development is likely to reflect changes in protein function rather than in expression domains. Later in development, *sox17* is expressed in anterior and posterior lateral plate mesoderm (LPM), posterior vasculature, and primitive erythrocytes. However, *sox32* substantially lacks these expression domains (Chung et al. 2011). Since mammalian *Sox17* is expressed in similar LPM and endothelial populations to *sox17* (Matsui et al. 2006; Saba et al. 2019), it seems likely that subfunctionalization has occurred at the gene regulatory level, leading to *sox17* but not *sox32* maintaining this portion of the ancestral expression pattern.

Sox32 is considered the master regulator of endoderm identity in zebrafish. Loss-of-function mutations in *sox32* lead to a profound loss of endoderm progenitors and consequent absence of the respiratory tract and gut, and its associated organs, including the liver and pancreas. Instead, presumptive endoderm cells differentiate into mesoderm (Alexander et al. 1999; Dickmeis et al. 2001). Conversely, *sox32* overexpression (OE) through mRNA injection during early development is sufficient to respecify presumptive mesodermal cells to endodermal fates, resulting in ectopic endoderm formation (Kikuchi et al. 2001). Furthermore, transplantation studies show that cells from *sox32* mRNA-injected donor zebrafish embryos preferentially incorporate into developing endoderm tissues in wild-type hosts, and they are capable of endoderm restoration in Sox32-deficient hosts (Kikuchi et al. 2001; Aoki et al. 2002; Stafford et al. 2006).

Similarly, *Sox17* null mutant mice are embryonic lethal and show narrower expression domains of definitive endoderm markers in the embryonic gut, indicating that loss of Sox17 results in depletion of the definitive endoderm (Kanai-Azuma et al. 2002). Furthermore, ectopic expression of SOX17 in mouse and human embryonic stem cells causes differentiation toward the extraembryonic and definitive endoderm fates, respectively (Séguin et al. 2008; Niakan et al. 2010; McDonald et al. 2014). Thus, phenotypic evidence suggests functional similarity between SoxF subfamily members, with both mammalian SOX17 and zebrafish Sox32 inducing endoderm cell identity within their respective species. However, a molecular functional comparison is lacking.

In zebrafish, Sox32 is also required for correct specification of the Dorsal Forerunner Cells (DFCs)—progenitors of the LR organizer, which is essential for correct positioning of endoderm-derived organs within the body cavity (Alexander et al. 1999; Essner et al. 2005). Specifically, the DFCs give rise to the zebrafish laterality organ, known as Kupffer's Vesicle (KV) (Cooper and D'Amico 1996; Melby et al. 1996; Essner et al. 2005; Warga and Kane 2018). KV function involves cilia-mediated fluid flow in an anticlockwise direction, which leads to unilateral induction in the left LPM of Nodal (Spaw in teleosts [Long et al. 2003; Montague et al. 2018]). Defects in KV formation and cilia function lead to abnormalities in Nodal expression and consequent laterality defects affecting organs including the brain, heart, and pancreas (Essner et al. 2005; Kramer-Zucker et al. 2005). As a downstream target of Sox32, Sox17 is implicated in KV function (Aamar and Dawid 2010), and unlike its paralog, is expressed in the LPM at segmentation stages (Chung et al. 2011). Similarly, in mice, Sox17 is implicated in the establishment of LR patterning. LR defects in *Sox17*^−/−^ mutants are caused both indirectly through inability to maintain gut endoderm, which is involved in the transfer of LR asymmetry from the node to LPM, and directly through morphogenesis defects in the mouse laterality organ (the node) (Kanai-Azuma et al. 2002; Saund et al. 2012). While there is evidence that mammalian gut endoderm transfers LR signals from the node to the LPM (Saund et al. 2012), to the best of current knowledge, the gut does not have such a role in fish. Thus, given a consistent requirement for mammalian SOX17 and zebrafish Sox32 in early endoderm development, and mammalian SOX17 and zebrafish Sox32 and Sox17 in LR organizer formation (node and KV), it appears likely that the common ancestral Sox17 had a composite role, being required in both endodermal and LR organizer tissue domains. The ancestral gene also likely had a role in the formation of LPM derivatives, given conserved *Sox17*/*sox17* expression in LPM, though the functions of zebrafish *sox17* in LPM and its derivatives, such as heart and vasculature, remain elusive.

Building on previous phenotypic observations and to gain novel insights at the molecular level, here we examine whether SoxF factors exhibit functional conservation across species and identify functional domains within the zebrafish Sox32 and Sox17 proteins that are required for their respective developmental roles in endoderm specification and LR patterning. We performed molecular dissection and hybrid protein functional analyses of zebrafish Sox32 and Sox17 to draw functional comparisons with human SOX17 (hSOX17). We find that Sox32 shares limited functional similarities with hSOX17 or Sox17, with neither of the latter two able to induce endoderm specification in the zebrafish embryo. Sox17 and hSOX17 also differ from each other in terms of molecular capabilities. In detail, Sox32's fundamental role in initiating the zebrafish endoderm GRN appears to be linked to divergence in its HMG domain. We also show how changes in putative helical peptides within their C-terminal domains (CTDs) account for the zebrafish paralogs’ differing functional roles, including in cell fate specification and organ placement. Overall, our analyses reveal how divergence in specific protein domains underpins distinct TF target gene repertoires. We thus elucidate the SoxF molecular evolutionary changes that determine endoderm GRN specificity in the ray-finned fish and mammalian lineages.

## Results

### Sox32 Likely Emerged Through Duplication of the Ancestral *sox17* Gene in the Common Ancestor of the *Actinopterygii*

To determine the relationship between Sox32 and other SoxF family members, we performed phylogenetic analyses of all identifiable orthologs of *sox7*, *sox17*, *sox18*, and *sox32* across 40 species sampled from all major branches of the vertebrate species tree. As expected, this revealed distinct branches corresponding to the four SoxF TFs, with Sox32 orthologs only identifiable in ray-finned fish species, and Sox32 exhibiting the greatest similarity to Sox17 (Fig. S1). To more comprehensively determine the prevalence of *sox32* in vertebrate species, we searched for orthologs of *sox32* in genomic data available at Ensembl. As expected, high-confidence orthologs of *sox32* were detected in the overwhelming majority (63/65) of ray-finned bony fish species, but not in non-fish species, cartilaginous fish, or lobe-finned fish (Ensembl release 113). Since the majority (63/65) of ray-finned fish genomes in Ensembl correspond to teleost species, we broadened our taxonomic sampling of the *Polypteridae*, *Acipenseriformes*, and *Holostei* (GenBank) to ensure adequate coverage of non-teleost ray-finned bony fish (Fig. S1). Our analyses reveal that the majority of species across clades within the ray-finned fish have recognizable homologues of *sox32* (Fig. S1).

Analysis of synteny across ray-finned fish species reveals that *sox32* is consistently adjacent to *sox17*, suggestive of a small-scale tandem duplication event, as previously suggested by analyses restricted to teleost species (Fig. S2) (Voldoire et al. 2017). However, based on tree topology, it is not clear whether *sox32* emerged in the last common ancestor of all ray-finned fish, followed by rapid divergence, or if *sox32* was present in the last common ancestor of all jawed vertebrates and was subsequently lost in cartilaginous fish and lobe-finned fish. To explore this, we used GeneRax, which uses maximum likelihood to reconcile the gene tree with the species tree (Morel et al. 2020). Reconciliation of the *SoxF* family gene tree (Fig. S1) with the classically accepted vertebrate species tree (Figs. S2 and S3) suggests the following: A duplication of the ancestral *SoxF* gene occurred at the base of the vertebrates, producing two lineages: a *Sox7*-like gene and a *Sox17*/*Sox18*/*Sox32* ancestor. Both genes were retained in jawless vertebrates (*Cyclostomes*). Later, in the ancestor of jawed vertebrates (*Gnathostomes*), the *Sox17*/*Sox18*/*Sox32* ancestor duplicated to give rise to *Sox17* and *Sox18*, which are conserved across all jawed vertebrates. Similarly, the *Sox7*-like gene was also duplicated in the *Gnathostome* ancestor, generating two *Sox7* paralogs. One of the *Sox7*-like paralogs was subsequently lost in the bony vertebrate lineage (*Osteichthyes*), while cartilaginous fishes (*Chondrichthyes*) retained both *Sox7*-like copies. Finally, *Sox32* arose as a lineage-specific duplication within the ray-finned fishes (*Actinopterygii*) (Fig. S3).

Overall, we conclude that tandem duplication of ancestral *sox17* most likely occurred early in the evolution of ray-finned fish, leading to *sox32* and *sox17*. Rare examples of subsequent loss of *sox32* also exist. A notable ray-finned bony fish lacking *sox32* is the spotted gar (*Lepisosteus oculatus*) (Braasch et al. 2016). Our analyses therefore also support a possible secondary loss of *sox32* restricted to the *Lepisosteidae* lineage (Figs. S1 and S2). Furthermore, our analyses suggest higher rates of evolutionary divergence of Sox32 compared to Sox17, potentially suggestive of neofunctionalization post-duplication (Fig. S1). We therefore selected hSOX17 for functional comparison with zebrafish Sox17 on the basis of its similarity to Sox17 in other tetrapods as well as its previously studied properties in human endoderm specification (Séguin et al. 2008).

#### hSOX17 Cannot Induce Endoderm Identity in Zebrafish Embryos

**Fig. 1.**
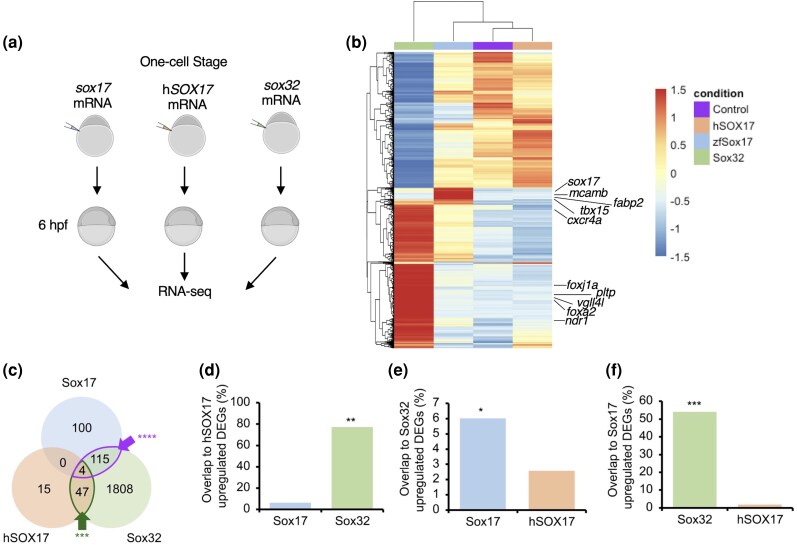
Sox32, Sox17, and hSOX17 are capable of inducing largely distinct gene sets in the early zebrafish embryo. a) Experimental schematic. b) Heatmap of all 3,866 DEGs (Bonferroni-adjusted *P* < 0.05, Tables S2 to S4), displayed as row *Z*-score calculated from median counts from *N* = 2 per condition. See Fig. S6 for an equivalent heatmap illustrating log2 fold-changes relative to control. c) Venn diagram showing overlap of genes (Table S5) significantly upregulated by each TF (Bonferroni-adjusted *P* < 0.05). Overlaps significantly greater than expected according to Fisher's Exact test are indicated. **** *P* < 0.0001. d) to f) Bar charts indicating percentage overlap of upregulated DEGs to d) hSOX17, e) Sox32, and f) Sox17 upregulated DEGs. Bars represent the percentage of genes upregulated by the factor on the *y* axis, also upregulated by the factor on the *x* axis; note the TF-specific *y* axis scale for each chart. Statistical tests to determine whether the overlap was significantly greater with one factor on the *x* axis compared to the other were carried out using Fisher's Exact test. *P < 1 × 10^−4^; **P < 5 × 10^−18^; ***P < 1 × 10^−39^; and ****P < 1 × 10^−71^. Panel A created in BioRender. Johal, S. (2025) https://BioRender.com/yhnlwz6.

Although hSOX17 and Sox32 can induce endoderm in humans and zebrafish, respectively, these orthologs’ molecular capabilities have not been tested in a directly comparable biological context. Also, it is unknown whether Sox17, downstream of Sox32, can induce endoderm gene expression directly. To address this, we injected equimolar quantities of mRNA encoding hSOX17, Sox32, or Sox17 into the zebrafish embryo at the one-cell stage, followed by RNA-seq analysis at 6 h postfertilization (hpf), the stage by which endoderm has been specified in wild-type embryos (Fig. 1a).

Sox32 OE elicited widespread changes in gene expression compared to Sox17, with hSOX17 affecting relatively few genes (Fig. 1b to c, Fig. S5 and S6, Tables S2 to S6). We observed a small but significant overlap in upregulated transcripts between Sox32 and hSOX17, and Sox32 and Sox17, but not between hSOX17 and Sox17 (Fig. 1c-f). However, we note that while there are overlaps in the gene sets significantly upregulated in these experiments (Fig. 1c), there are clear quantitative differences in their degree of induction by the Sox factors. Thus, while Sox32 appears functionally more similar to both hSOX17 and Sox17 than hSOX17 and Sox17 are to each other in the context of the early zebrafish embryo, overall, the three Sox factors appear to have largely distinct functional capabilities in these experiments.

To examine the pathways and processes influenced by OE of these TFs, we performed functional and anatomical annotation analysis of the differentially expressed genes (DEGs), including comparison to established cell identity markers from single-cell (sc)RNA-seq time course analysis of the early embryo (Wagner et al. 2018). This revealed largely distinct results for each TF, further supporting the notion that they are functionally distinct in this context (Fig. S7 and S8). As expected, transcripts upregulated by Sox32 OE were significantly enriched for terms associated with endoderm development (Fig. S8). This includes 6 hpf “nondorsal involuted anterior,” which contains endoderm progenitors, and 8 hpf endoderm. Transcripts induced by Sox32 also showed strong enrichment for 8 hpf DFC markers, suggesting that Sox32 is not only necessary (Alexander et al. 1999) but also sufficient to drive DFC gene expression. In comparison, Sox17 OE results in a less profound effect on endoderm marker expression, with no significant effect on 6 hpf nondorsal involuted anterior marker induction but modest significant enrichment for endoderm markers at 8 hpf and for DFC markers, consistent with a role in KV formation and function (Fig. S8). Meanwhile, genes upregulated after hSOX17 ectopic expression did not yield any significantly enriched gene ontology (GO) terms, and downregulated genes do not pertain to endoderm but may be indicative of misregulation of Wnt signaling (Fig. S7).

Beyond endoderm development, analysis of transcripts upregulated by Sox17 revealed significant enrichment for terms associated with cardiovascular development, such as “Regulation of Sprouting Angiogenesis,” “Endothelial Tube Morphogenesis,” and “Endocardium” (Fig. S7). This is consistent with *sox17* expression patterns during cardiovascular development in both zebrafish and mammals (Chung et al. 2011; Saba et al. 2019) (Fig. S9, Video S1), and known *Sox17* loss-of-function phenotypes in mice vasculature and heart (Kanai-Azuma et al. 2002; Saba et al. 2019).

Thus, functional and anatomical enrichment analyses suggest Sox17 has different roles in early developmental processes compared to its paralog Sox32, and hSOX17 cannot induce endoderm specification in zebrafish.

#### Sox32 Has a Divergent HMG Domain and Longer C-terminal Helix

**Fig. 2.**
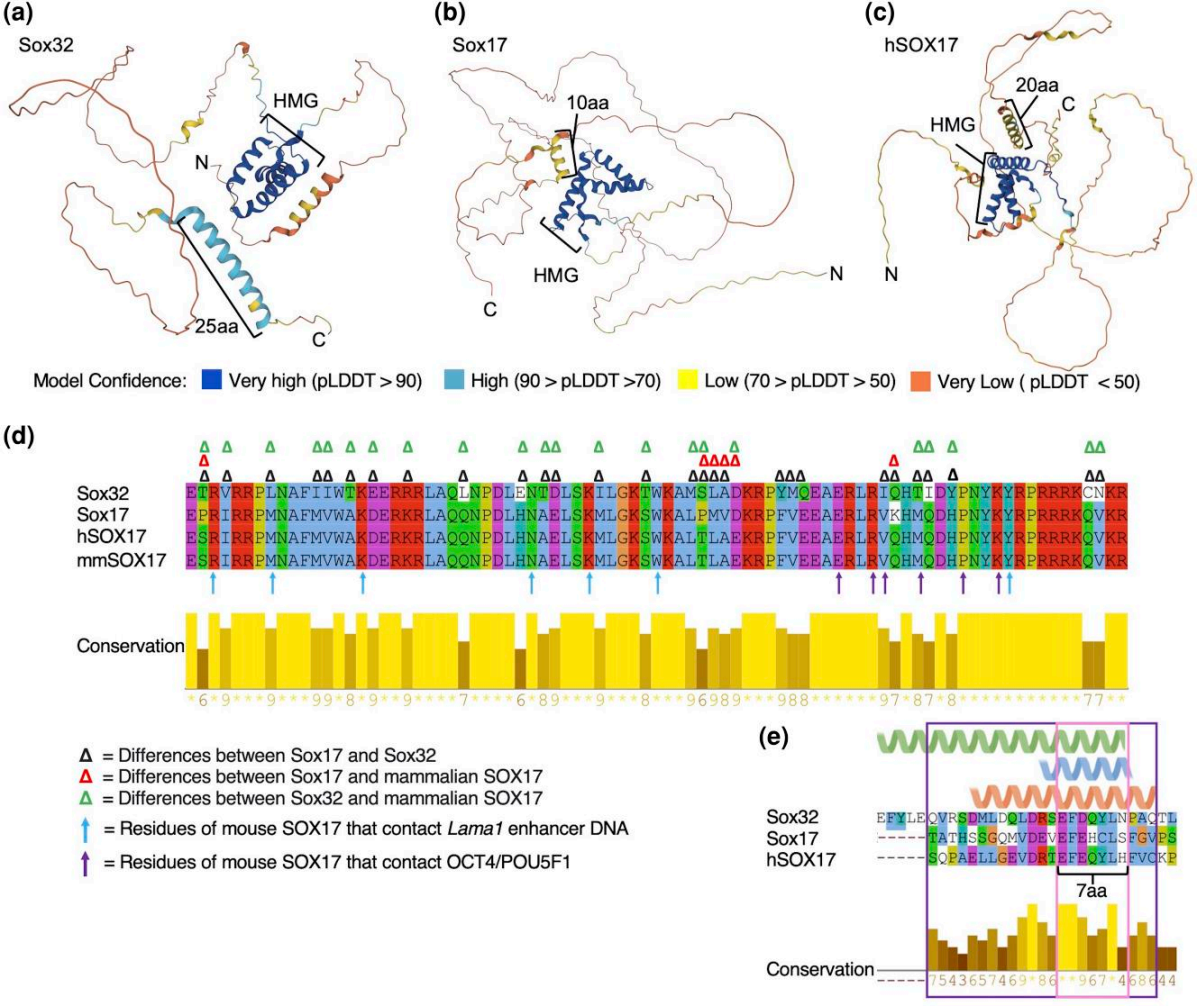
AlphaFold predicts C-terminal helical domains within Sox32, Sox17, and hSOX17. a) to c) AlphaFold models coloured by model confidence as shown in the key. DNA-binding HMG domains and C-terminal helices of different lengths are annotated. d) Alignment of HMG domains of Sox32, Sox17, hSOX17, and *Mus musculus* (mm)Sox17. Alignment was carried out using Clustal Omega in Jalview, and color-coded based on conserved amino acid properties. Conservation levels calculated according to the AMAS are displayed. Delta symbols denote differences in amino acids as per the key. Arrows indicate mapped amino acid contact sites between mouse SOX17 and the *Lama1* enhancer (blue), and equivalent SOX2 sites and OCT4/POU5F1 protein (purple) (Kikuchi et al. 2001; Palasingam et al. 2009; Ng et al. 2012). e) Alignment of the CTD helical domain region for Sox32, Sox17, and hSOX17. Amino acids within AlphaFold predicted helical domains annotated: green = Sox32; blue = Sox17; and peach = hSOX17. The 7-aa peptide previously shown to be required for Sox32 induction of endoderm fate (Zhao et al. 2013) is annotated. The aligned broader helical region and 7-aa region are outlined in purple and pink, respectively.

To determine which structural motifs may explain the functional differences between Sox32, Sox17, and hSOX17, we analyzed AlphaFold structural predictions and performed protein sequence alignments (Fig. 2a to e). 3D structural models indicate a very high confidence prediction in the center of all three proteins corresponding to the HMG domain. The HMG domains of Sox17 and hSOX17 are most similar, with 92% amino acid identity, while the Sox32 HMG domain differs from both Sox17 and hSOX17 with 67% and 69% identity, respectively (Fig. 2d). Interestingly, 44% of amino acid differences between Sox32 and Sox17 correspond to changes in chemical properties that may influence the 3D folding of the protein, resulting in exposure of different residues to binding partners. For example, there are three polar glutamine (Q) residues in Sox17 and hSOX17 that have been substituted for nonpolar leucine (L), isoleucine (I), or cysteine (C) in the equivalent location in the Sox32 HMG domain. Other examples include nonpolar alanine (A) and valine (V) in Sox17 and hSOX17, replaced by polar threonine (T) and asparagine (N) in Sox32. Elsewhere, negatively charged glutamic acid (E) and positively charged histidine (H) in Sox17 and hSOX17 have been replaced with uncharged glutamine (Q) and negatively charged glutamic acid (E), respectively (Fig. 2d).

Phylogenetic analysis of Sox17/32 HMG domains across fish, mammalian, avian, and amphibian species further supports divergence of the Sox32 HMG domain (Fig. S10). Sox32 HMG domains across ray-finned fish form a monophyletic grouping distinct from the other vertebrate Sox17 HMG domains (Fig. S10). Our analyses additionally indicate that ray-finned fish Sox17 HMG domains form a distinct subclade relative to Sox17 HMG domains from other species (Fig. S10). Ray-finned fish Sox17 HMG domains have therefore potentially undergone a degree of divergence concurrent with alterations to Sox32.

To determine whether differences in the HMG domains of these proteins likely reflect positive, purifying, or neutral selection, we performed analyses of rates of nonsynonymous changes, using the single SoxF factor in the tunicate *Ciona intestinalis* as an outgroup. This revealed that nonsynonymous substitution rates are significantly greater in Sox32 HMG domains compared to both fish Sox17 and tetrapod HMG domains (*P* = 1.90 × 10^−2^ and 1.48 × 10^−2^ respectively). Conversely, fish and tetrapod Sox17 HMG domains do not show significantly different rates of nonsynonymous changes (*P* = 0.95). Similar results were obtained using the elephant shark Sox17 HMG domain coding sequence as an outgroup (Sox32 vs. fish Sox17 HMG domains: *P* = 5.53 × 10^−6^; Sox32 vs. tetrapod Sox17 HMG domains: *P* = 1.04 × 10^−6^; fish Sox17 vs. tetrapod Sox17 HMG domains: *P* = 0.46; Table S7). Overall, this suggests the HMG domains of ray-finned fish and tetrapod Sox17 have been subject to purifying selection to maintain the resulting amino acid sequence. Conversely, the *sox32* HMG domain coding sequence shows a significantly higher rate of nonsynonymous change. This suggests that the Sox32 HMG domain has not been subject to the same selective constraints and thus duplication of the ancestral gene permitted sequence changes that potentially contributed to altered molecular function.

Another notable structured region within Sox32 is a predicted 25-aa C-terminal helix (Fig. 2a). C-terminal helices were also predicted in Sox17 (Fig. 2b) and hSOX17 (Fig. 2c), albeit the confidence of the prediction is lower and they are shorter (Fig. 2e). Amino acid sequence alignments of the putative helices suggest the overall level of conservation is low, although they do encompass a 7-aa peptide region previously recognized to be conserved throughout the SoxF subfamily, including in Sox7 and Sox18 (Kikuchi et al. 2001; Sinner et al. 2004) (Fig. S11). This 7-aa zebrafish Sox32 peptide (EFDQYLN, Fig. 2e) has previously been shown to be required for Sox32 induction of endoderm fate (Zhao et al. 2013). The core 6-aa EFDQYL sequence is conserved in *Xenopus* Sox17β, where it has been shown to interact with Wnt pathway effector β-catenin (Sinner et al. 2004). To determine whether the C-terminal helices and particularly putative β-catenin-interacting peptides of Sox17 and 32 may have been subject to different selective pressures, we compared synonymous and nonsynonymous substitutions across a diverse panel of fish and tetrapod orthologs (Fig. S12). While the length of this short peptide precludes statistical analyses, some striking patterns emerge. A clear consensus sequence of EFEQYL is broadly conserved across vertebrate clades in both Sox17 and (where present) Sox32. Some lineage-specific, conservative, single amino acid changes exist, including E3D in zebrafish and bowfin Sox32 and *Xenopus* Sox17β, and F2L in Sox32 of *Acipenseriformes* fish species. Striking exceptions are found in a subset of teleost fish Sox17. While the consensus of EF(E/D)QYL is common in Sox32 and Sox17 of most species, Atlantic cod (*Gadus morhua*) Sox17 completely lacks this peptide, while nonsynonymous changes in zebrafish *sox17* lead to amino acids with different physicochemical properties in Sox17. Specifically, the uncharged polar glutamine (Q) and polar tyrosine (Y) in Sox32 and hSOX17 (and most other species) are replaced with positively charged histidine (H) and nonpolar cysteine (C) in Sox17 (Fig. 2e, Fig. S12). These recent, teleost-specific alterations in Sox17 could potentially foster evolutionary changes in Sox17 function in a subset of fish species.

Having identified the Q4H and Y5C substitutions in zebrafish, a member of the *Cypriniformes*, we then analyzed *sox17* orthologs throughout this order to trace their evolutionary origin (Fig. S13). This revealed that families within *Cypriniformes*, including *Catostomidae* and *Cobitidae*, have maintained the EFEQYL consensus. However, the consensus in *Cyprinidae* species is EFEQCL, while *Danionidae* species, including zebrafish, have EFEHCL. *Cyprinidae* and *Danionidae* share a more recent common ancestor with each other than with *Catostomidae* and *Cobitidae* (Stout et al. 2016). It therefore seems likely that the Y5C change occurred in the common ancestor of *Cyprinidae* and *Danionidae*, followed by Q4H in *Danionidae*.

Overall, we conclude that the Sox32 HMG domain shows a higher degree of divergence from the common ancestor than Sox17 or hSOX17. Additionally, we identify putative CTD helices of differing lengths between these Sox factors that encompass an EF(E/D)QYL consensus peptide region of known importance in Sox32. This peptide shows a greater degree of divergence in zebrafish Sox17, which we explore functionally below.

#### Sox32 Specificity for the Endoderm GRN is Driven by HMG Domain Divergence

**Fig. 3.**
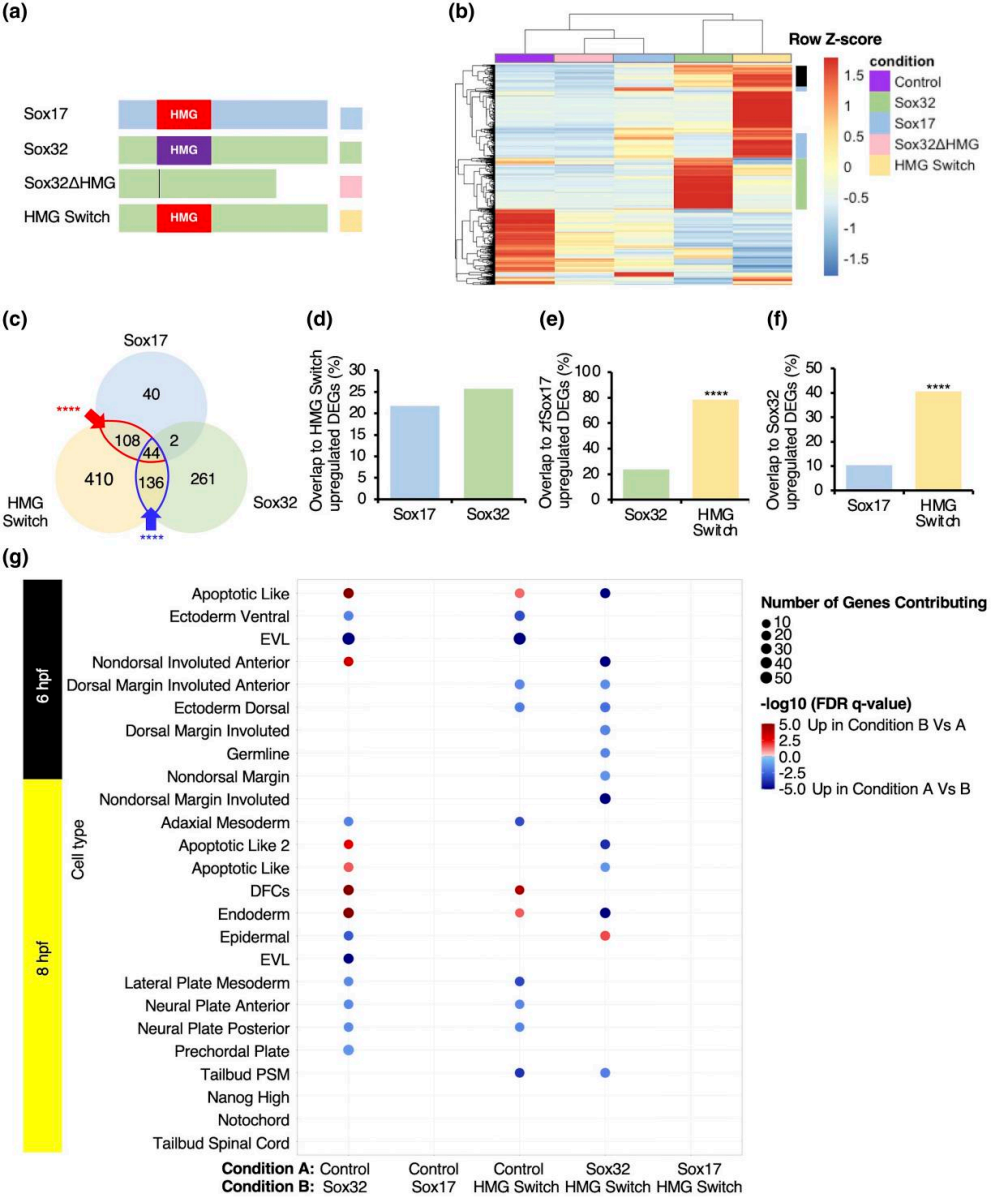
Differences between the Sox32 and Sox17 HMG domains are critical to the ability of Sox32 to induce endoderm and DFC gene expression. a) Protein models depicting constructs injected at the one-cell stage, followed by RNA-sequencing at 6 hpf. b) Heatmap of all 1,572 DEGs (Bonferroni-adjusted *P* < 0.05, Tables S8 to S12), depicted as median counts from *N* = 2 per condition. Green sidebar: induction of genes requiring the Sox32 HMG domain. Blue sidebar: induction of genes requiring the Sox17 HMG domain. Black sidebar: genes induced by Sox32 with either Sox32 or Sox17 HMG domain. c) Overlap of upregulated DEGs. Overlaps greater than expected according to Fisher's Exact test are indicated—****P < 0.0001. d) to f) Percentage overlap of upregulated DEGs to d) HMG Switch, e) Sox17, and f) Sox32 upregulated DEGs. Bars represent the percentage of genes upregulated by the factor on the *y* axis, also upregulated by the factor on the *x* axis. Statistical tests to determine whether the overlap was significantly greater with one factor on the *x* axis compared to the other were carried out using Fisher's Exact test. **** *P* < 0.001. g) GSEA carried out against cell type markers defined by scRNAseq at 6 and 8 hpf (Wagner et al. 2018). EVL, enveloping layer; PSM, presomitic mesoderm.

Compared to its homologues, Sox32 has a unique property allowing it to induce the zebrafish endoderm GRN (Fig. 1), and divergence in the DNA-binding HMG domain (Fig. 2d) may confer this function. To examine this, we tested the ability of a hybrid protein (HMG Switch), consisting of the Sox32 N- and CTDs flanking the Sox17 HMG domain, to induce endoderm gene expression, compared to wild-type Sox32 and Sox17 proteins and a Sox32 protein lacking the HMG domain (Sox32ΔHMG, Fig. 3a). These proteins’ transcriptional effects were assayed by RNA-seq after early ectopic mRNA injection (Fig. 3b).

As expected in the absence of a DNA-binding domain, Sox32ΔHMG had limited effects on gene expression compared to Sox32 (Fig. 3b). In contrast, HMG Switch-induced genes show significant overlap with both Sox32- and Sox17-induced genes, with either paralog's HMG domain sufficient for the induction of a core set of 180 genes (Fig. 3c). This is further supported in pairwise comparisons (Fig. 3d to f). In fact, the majority of Sox17-induced genes are also induced by the HMG Switch protein (Fig. 3c: 152 of 194 DEGs), albeit to a different quantitative degree (Fig. 3b), underscoring the importance of the HMG domain for specific induction of Sox17 targets. However, the HMG Switch protein has a distinct TF profile, with over half (58.8%) of the genes it induces here not induced by either Sox32 or Sox17. Furthermore, 58.9% of Sox32-induced genes are not induced by HMG Switch (Fig. 3b and c). Taken together, this indicates that amino acid differences distinguishing the Sox32 and Sox17 HMG domains confer significant functional differences.

Functional annotation of induced genes further illustrates the limits of the HMG Switch protein to recapitulate Sox32's induction of specific cell identities (Fig. 3g). Sox32 has a stronger capacity to significantly induce markers of endoderm and DFC cell types than the HMG Switch, and this enrichment occurs at the expense of other cell types, such as the adaxial mesoderm and ventral ectoderm. This suggests the Sox32 HMG domain shows endodermal gene specificity and is required to induce cell fate changes. Furthermore, while HMG Switch provides significant induction of endoderm markers compared to the control, Sox32 exhibits significantly greater induction of endoderm markers compared to HMG Switch (Fig. 3g).

Overall, we conclude that the divergence of the Sox32 HMG domain from ancestral Sox17 is critical in conferring key functional differences, allowing Sox32 to induce endoderm and DFC gene expression within the zebrafish embryo.

#### The Sox32 25-aa C-terminal Helix Is Required for Induction of Endoderm and DFC Marker Genes While the NTD Has Target-Specific Functions

Beyond the HMG domain, we also find evidence that the flanking protein regions influence target gene induction. One-fifth of Sox17-induced genes are unique (Fig. 3c), implicating other domains in determining the full repertoire of TF-specific target genes. This is further corroborated by the sizable overlap in induced targets between the HMG Switch and Sox32 proteins despite differing HMG domains (Fig. 3c: 136 targets). We therefore next explored which peptides outside the Sox32 HMG domain are required for target gene induction.

The Sox32 N-terminal domain (NTD) contains a conserved Glu-Lys-Arg phosphorylation motif that, when phosphorylated in response to Fibroblast Growth Factor (FGF) signaling, leads to attenuation of Sox32 function, antagonizing endoderm specification (Poulain et al. 2006). Furthermore, examination of conservation across *sox32* orthologs revealed conservation of a 30-aa region within the Sox32 CTD, which we consider in two parts. Specifically, previous studies have identified a 7-aa β-catenin interacting peptide, with higher conservation between Sox32 and hSOX17 than Sox17 (Kikuchi et al. 2001; Sinner et al. 2004), that is required for Sox32 induction of ectopic endoderm upon OE (Zhao et al. 2013). An upstream 23-aa peptide conserved among *sox32* orthologs remains unstudied (Fig. S11). Furthermore, AlphaFold predicts a high confidence 25-aa-long CTD helix which partly encompasses these two peptide regions (Fig. 2).

**Fig. 4.**
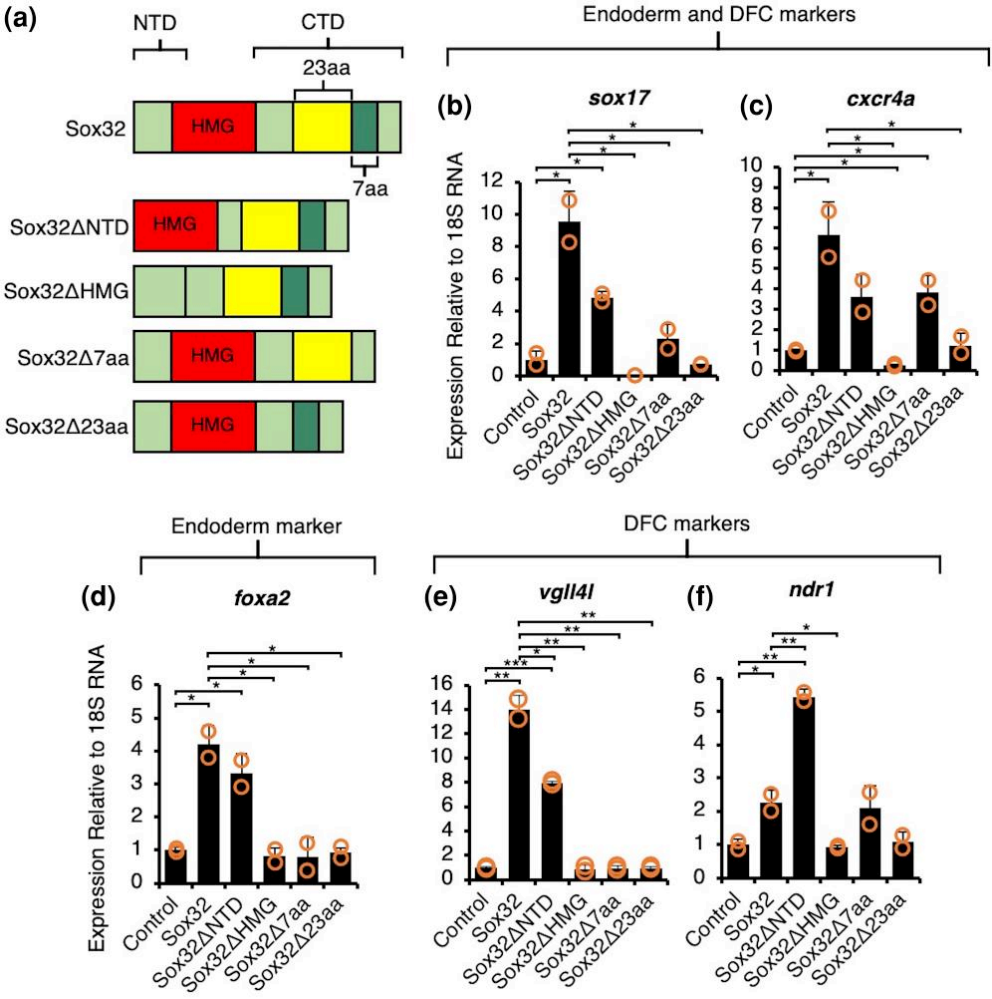
Putative helical CTD peptides highly conserved between Sox32 orthologs are required for induction of downstream endoderm and DFC marker genes, while NTD function is target-specific. a) Protein models of Sox32 deletions injected at the one-cell stage and RNA extraction followed by qPCR at 6 hpf. b) to f) qPCR analysis of endoderm and DFC (b, c) endoderm only (d), and DFC only (e to f) markers. Bar charts depict the mean fold change relative to uninjected controls, error bars ± one standard deviation, and orange circles indicate individual datapoints. Statistical tests on biological duplicate samples were carried out using Student's *t*-test. Raw *P*-values are indicated—*P < 0.05, **P < 0.01, and ***P < 0.001.

To examine the importance of these identified peptides to Sox32 target induction, we carried out specific domain deletions. We then analyzed the ability of these domain-deleted mRNAs injected at the one-cell stage to induce five diagnostic endoderm and DFC cell marker genes relative to full length (FL)-Sox32 by 6 hpf (Fig. 4a). All selected markers show induction by FL-Sox32 in RNA-seq experiments (Fig. 1b), with tissue-specific expression in the endoderm only (*foxa2*) (Alexander et al. 1999), endoderm and DFCs (*sox17*, *cxcr4a*) (Kikuchi et al. 2000; Thisse et al. 2001) or DFCs only (*vgll4l*, *ndr1*) (Rebagliati et al. 1998; Thisse et al. 2001).

Consistent with our global RNA-seq profiling (Fig. 3b), Sox32 can strongly induce all marker genes, and the HMG domain is essential (Fig. 4b-f: FL-Sox32 and Sox32ΔHMG constructs). Equally, both peptides within the CTD helix are required, as Sox32Δ7aa and Sox32Δ23aa OE show no deviation in expression of target genes compared to control embryos (Fig. 4b-f), except for significant induction of *cxcr4a* still attained with the Sox32Δ7aa construct.

The role of the NTD appears more complex, with differing roles depending on the target gene. Overall, the NTD would seem dispensable, as Sox32ΔNTD can significantly induce all analyzed genes compared to the control, and with comparable levels of expression of the endoderm-only marker *foxa2* (Fig. 4b). However, the Sox32ΔNTD construct only induced the dual endoderm/DFC markers *sox17* and *cxcr4a* to about half the level of FL-Sox32 (Fig. 4c-d). Furthermore, for DFC-specific marker genes, relative induction by FL-Sox32 and Sox32ΔNTD differed, with significantly greater induction of *vgll4l* with the full-length protein but, conversely, significantly higher *ndr1* levels with the NTD-deleted construct (Fig. 4e-f). Thus, the NTD may offer a tuneable interface for context-specific target gene regulation by Sox32, with the DFCs potentially evincing subtle modulation for different marker genes.

To further explore the ability of Sox32 to induce endoderm and DFC markers, and whether its effects are spatially restricted in the embryo, we also performed whole-mount in situ hybridization (WISH) for endoderm and DFC marker *sox17* and endoderm and axial chorda mesoderm marker *foxa2*. Consistent with the above results, we found very broad ectopic expression of both *sox17* and *foxa2* induced by both FL-Sox32 and Sox32ΔNTD in all embryos, but not by Sox32ΔHMG, Sox32Δ7aa, or Sox32Δ23aa. Furthermore, Sox32ΔHMG and Sox32Δ23aa OE led to reduced *sox17* expression in a significant subset of embryos (Fig. S14). We conclude that there is limited embryo-to-embryo variability in terms of response to Sox32 OE, and that Sox32ΔHMG and Sox32Δ23aa may potentially exert dominant negative effects.

#### Divergence of C-terminal Peptides Confers Sox32 and Sox17 Differential Target Specificity

**Fig. 5.**
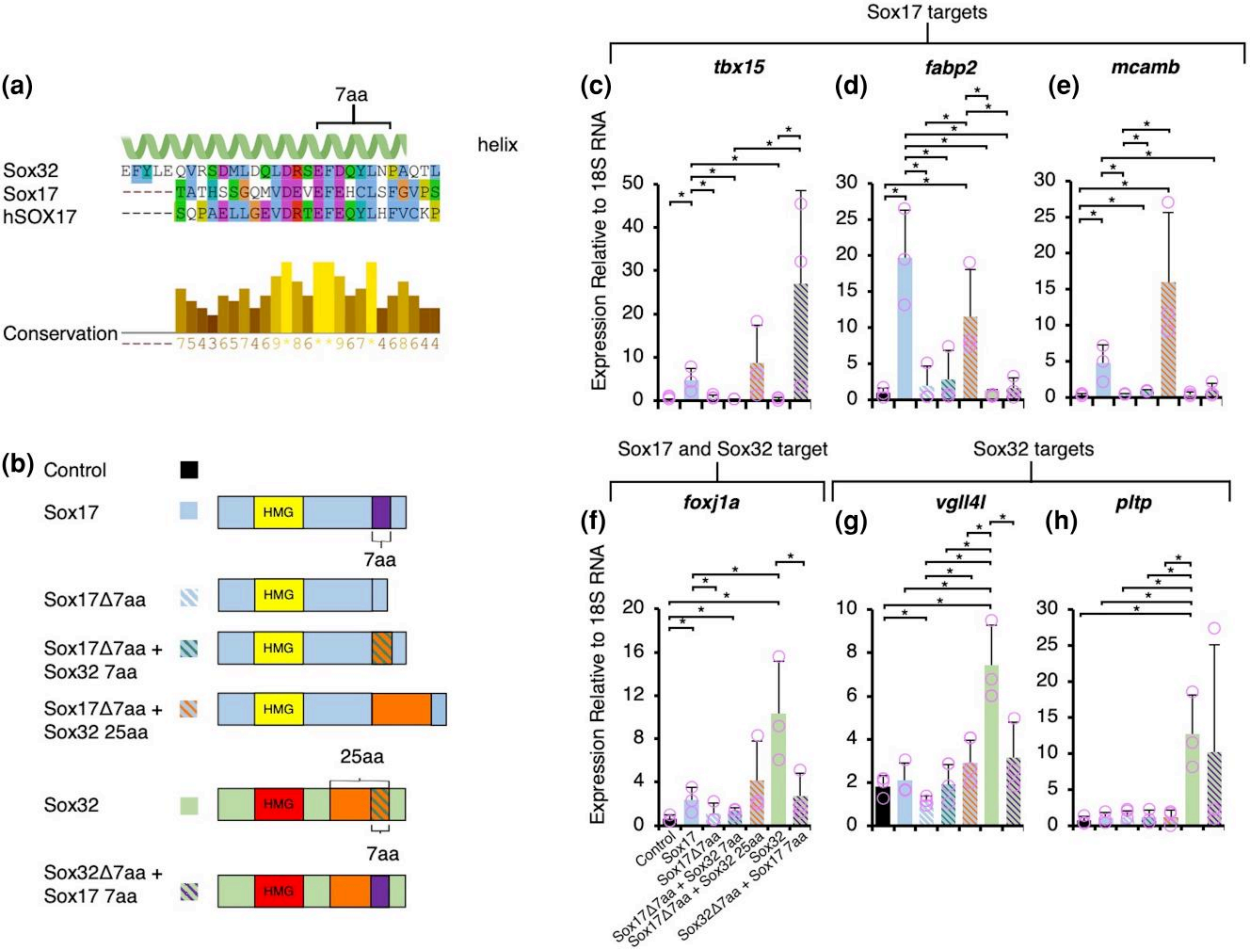
Divergence of 7-aa CTD putative helical peptides confers differential target specificity of Sox32 and Sox17. a) Alignment of Sox32 CTD helix (25 aa) to Sox17 and hSOX17 with conservation indicated. Alignment carried out using Clustal Omega in Jalview and color-coded based on conserved amino acid properties. Conservation levels calculated according to the AMAS are displayed. b) Protein models of Sox17 and Sox32 domain deletions and switches injected at the one-cell stage, and RNA extraction followed by qPCR at 6 hpf. c) to h) qPCR of Sox17 targets (c to e), Sox17 and Sox32 targets (f), and Sox32 targets (g to h); sample condition colour code and order of bars corresponds to the legend in panel (b). Bar charts depict the mean fold change relative to uninjected controls, error bars ± one standard deviation, and pink circles indicate individual datapoints. Statistical tests on biological triplicate samples were carried out using Student's *t*-test. *P < 0.05 (raw *P*-values).

Sox32 requires its CTD helix for proper induction of its endodermal and DFC targets, specifically the 7-aa peptide (Fig. 4), which is also necessary to induce ectopic endoderm upon Sox32 OE (Zhao et al. 2013). This 7-aa region shows complete conservation to the zebrafish SoxF subfamily members Sox7 and Sox18 (Fig. S11) and high conservation to hSOX17. However, the 7-aa peptide is divergent in Sox17 (Fig. 5a). This poses a question of whether this divergence between Sox32 and Sox17 confers differential target specificity. To decipher this, we generated constructs in which the respective 7-aa CTD regions were deleted from both Sox32 and Sox17, and hybrid constructs were also generated with 7-aa peptides switched between the two TFs (Fig. 5b). Lastly, as the overall C-terminal helix structure also differs between Sox32 and Sox17 (Fig. 2), we also produced a construct with the longer 25-aa Sox32 CTD helical peptide replacing the Sox17 7-aa peptide (Fig. 5b).

Based on injections of each of the resulting mRNAs at the one-cell stage and qPCR analysis of target genes that distinguish Sox32 and Sox17 (Fig. 1b), we find that induction of Sox17 targets indeed requires the Sox17 7-aa peptide. Replacement with the Sox32 7-aa fails to elicit FL-Sox17 levels of induction (Fig. 5c-e). The replacement of the Sox17 7-aa peptide with the Sox32 25-aa peptide does not allow Sox17 to induce Sox32 targets (Fig. 5g-h). Therefore, while the Sox32 CTD helix is necessary for Sox32 induction of endoderm and DFC targets (Fig. 4), it is not sufficient in the context of the Sox17 protein. However, replacement of the Sox17 7-aa peptide with the Sox32 25-aa peptide does result in significant induction of Sox17 targets *fabp2* and *mcamb* compared to the control (Fig. 5d-e). We therefore conclude that the Sox32 25-aa peptide that makes up the Sox32 CTD helix appears to be a potent transactivation domain.

In addition to replacement of the Sox17 7-aa peptide with that of Sox32 not inducing Sox17 targets to the capacity of FL-Sox17 (Fig. 5c-e), we also see that replacement of the Sox32 7-aa peptide with the Sox17 7-aa peptide cannot induce *foxj1a* or *vgll4l* to the same level as FL-Sox32 (Fig. 5g). This indicates that both Sox32 and Sox17 require their own intrinsic 7-aa domain for efficient induction of their respective targets. Therefore, we suggest that divergence in C-terminal peptides during evolution led to differential Sox32 and Sox17 target specificity and induction.

#### Sox17 Is Necessary for Establishing Correct Brain, Heart, and Pancreas Laterality

Given the distinct profile of Sox17 as a TF that is not merely redundant to Sox32, what is the full biological scope of its function? Organ development requires not only endoderm specification, but also subsequent tissue placement. For many species’ organ systems, bilateral asymmetry is vital for correct function, including in humans (Sutherland and Ware 2009; Agarwal et al. 2021). Heterotaxia is a syndrome of abnormal abdominal and thoracic organ placement across the LR axis of the body, including the pancreas and heart (Aiello et al. 2007). In the brain, similar defects can alter patterns of gene expression and affect neural connectivity (Bianco and Wilson 2008).

Zebrafish Sox17 has previously been implicated in the establishment of LR asymmetry. Knockdown of Sox17 using an antisense morpholino (MO) was previously reported to result in abnormal pancreas placement, which could be rescued by co-injection with *sox17* mRNA to which the MO did not bind (Aamar and Dawid 2010). However, an analysis of zebrafish *sox17* function in genetic mutants is lacking. Furthermore, we aim to identify key domains of Sox17 required for correct LR patterning.

**Fig. 6.**
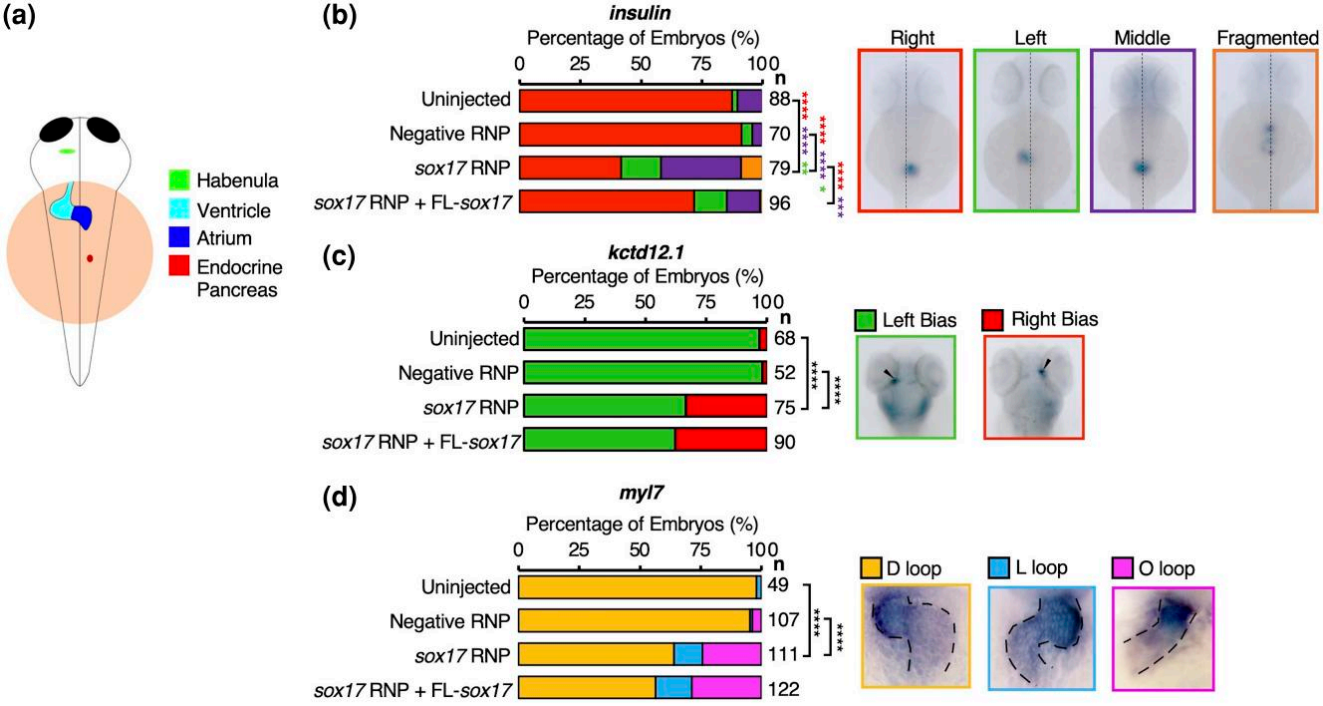
Sox17 CRISPants exhibit LR asymmetry defects. a) Diagram depicting correct asymmetry of organs (4 days postfertilization—dpf). *Ktcd12.1* expression in the brain shows a left habenula bias (green). The heart loops in a dextral (D-loop) fashion (blue). The endocrine pancreas is located to the right of the midline (red). b) to d) WISH analyzing the effect of Sox17 knockdown on endocrine pancreas placement relative to the midline (dotted line) at 48 hpf indicated by *insulin* (b); dorsal habenula *kctd12.1* asymmetric expression at 4 dpf—arrowheads indicate highest habenula expression (c); and heart looping at 48 hpf indicated by *myl7—*expression domain is outlined to highlight looping (d). Also shown are the phenotypic outcomes from rescue experiments from the co-injection of *sox17* exogenous mRNA along with CRISPR RNPs at the one-cell stage. Statistically significant differences in categorical scoring were inferred using Fisher's Exact test on independent biological triplicate datasets. Raw *P*-values are indicated—*P < 0.05, **P < 0.01, ***P < 0.001, and ****P < 0.0001. For b) red asterisks indicate a significant difference in the right-sided pancreas versus all other phenotypic outcomes, purple asterisks indicate a significant difference between middle-placed pancreas versus everything else, and green asterisks indicate a significant difference between left-sided pancreas and everything else. For c) to d) black asterisks indicate a significant difference between normal laterality and all other phenotypes. *n*, the number of embryos analyzed.

To further explore the role of Sox17 in LR asymmetry, we used an efficient method of G0 CRISPR (Wu et al. 2018) (Fig. S15) to disrupt the *sox17* locus by injection of Cas9–sgRNA complexes at the one-cell stage. We then analyzed LR patterning using WISH of marker genes. We assessed organ placement for the normally dextrally looped heart (myocardial marker *myl7* [Yelon et al. 1999; Bakkers 2011]), dextrally positioned pancreas (*insulin* expression [Argenton et al. 1999]), and sinistrally stronger gene expression in the habenular nuclei of the brain (*kctd12.1* expression [Sutherland 1982; Concha and Wilson 2001; Gamse et al. 2003; Gamse et al. 2005]) (Fig. 6a).

We find that Sox17 CRISPants show abnormal brain asymmetry, heart looping, and pancreas placement (Fig. 6b-d). This includes a significant increase in the proportion of embryos exhibiting a middle and left-sided pancreas compared to the control (Fig. 6b. We co-injected *sox17* mRNA into CRISPants at the one-cell stage to assess its ability to rescue the observed phenotypes. This resulted in a significant increase in embryos with normal right-sided pancreas placement. This occurred concurrently with a significant decrease in middle placement, while aberrant left-sided placement remained unaffected (Fig. 6b). This indicates that the middle placement of the pancreas can be rescued by exogenous *sox17* mRNA; however, left-sided pancreas, i.e. total reversal, cannot.

Neither brain asymmetry (Fig. 6c) nor heart looping (Fig. 6d) could be rescued by co-injection of exogenous *sox17* mRNA into CRISPants. To test whether exogenous Sox17 itself may be affecting LR patterning, we carried out OE studies through the injection of *sox17* mRNA across a broad range of concentrations. This indicated that Sox17 OE does not alter either brain asymmetry or heart looping (Fig. S16). The lack of rescue of abnormal phenotypes is therefore not due to the effect of Sox17 OE.

Going further, we considered whether the failure of *sox17* mRNA injection to rescue heart looping was due to underlying defects in adjacent tissue patterning. Sox32 mutants that lack anterior endoderm exhibit *cardia bifida* (Alexander et al. 1999), a condition in which independent left and right heart fields develop due to a failure of coalescing morphogenesis. We therefore analyzed our Sox17 CRISPants for expression of the anterior endoderm markers *foxa2* and *vgll4l.* Contrary to the reported Sox17 MO phenotype (Aamar and Dawid 2010), we found that the anterior endoderm is intact in Sox17 CRISPants (Fig. S17). Therefore, the lack of rescue of heart looping does not appear to be a secondary effect due to endoderm defects.

The failure of exogenous *sox17* mRNA to rescue defective pancreas positioning, brain asymmetry, or heart looping led us to test whether KV function can be rescued. A key early step in the establishment of LR asymmetry, which is indicative of KV function, is unilateral *spaw* expression in the left LPM. Consistent with a defect in organ placement, we found that exogenous *sox17* mRNA injection at the one-cell stage failed to restore left-sided *spaw* expression in Sox17 CRISPants at 18 hpf (Fig. S18). This suggests that the KV function is not restored by exogenous *sox17* mRNA. Since exogenous *sox17* mRNA is sufficient to restore right-sided placement of a proportion of pancreases that are aberrantly placed at the midline in Sox17 CRISPants, this suggests reduction of aberrant middle pancreas placement is independent of KV.

In conclusion, we have shown that Sox17 CRISPants have abnormalities in brain asymmetry, heart looping, and pancreas placement. Only an abnormal middle-placed pancreas can be rescued by co-injection of the *sox17* mRNA into CRISPants. The placement of the pancreas is determined by looping of the posterior foregut as a result of LPM migration, in which *sox17* is also expressed (Horne-Badovinac et al. 2003; Chung et al. 2011). Therefore, Sox17 likely has a role in gut looping, as exogenous *sox17* mRNA is sufficient to rescue, which we speculate may be linked to a function in the LPM.

#### Conserved C-terminal Peptides Are Required in Sox17 for Correct Pancreas Placement

**Fig. 7.**
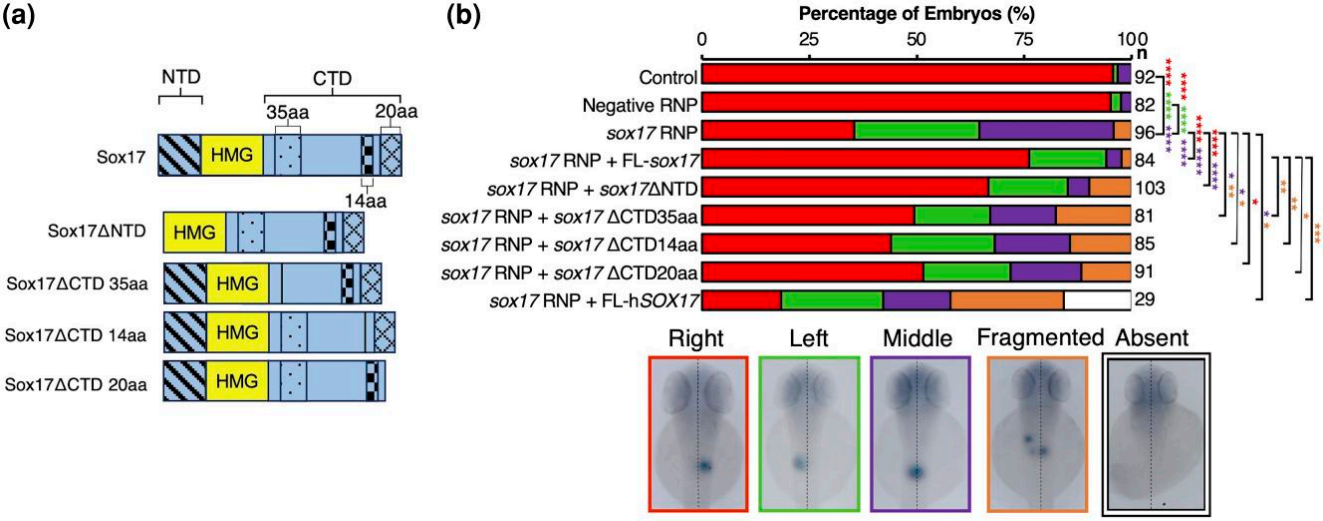
Sox17 CTD peptides are required for rescue of correct pancreas placement in Sox17 CRISPants. a) Diagram depicting protein models of Sox17 mutants. b) WISH at 48 hpf using insulin to assess the ability of Sox17 mutants in rescuing abnormal exocrine pancreas placement by co-injection of *sox17* exogenous mRNA alongside CRISPR RNPs at the one-cell stage. Statistically significant differences in categorical scoring were inferred using Fisher's Exact test on independent biological triplicate datasets. Raw *P*-values are indicated—*P < 0.05, **P < 0.01, ***P < 0.001, and ****P < 0.0001. Red asterisks indicate a significant difference in right-sided pancreas versus all other phenotypes, purple asterisks indicate a significant difference between middle-placed pancreas versus all other phenotypes, green asterisks indicate a significant difference between left-sided pancreas versus all other phenotypes, and yellow asterisks indicate a significant difference between fragmented *insulin*+ domains versus all other phenotypes. *n*, the number of embryos analyzed.

We have confirmed that Sox17 is required for organ asymmetry in zebrafish and that we can rescue pancreas placement through co-injection of *sox17* mRNA into CRISPants. We can therefore use this system to molecularly dissect which domains of Sox17 are required for its role in this process. We created *Sox17* mutant constructs with selected deletions based on high-level conservation among teleost and mammalian Sox17 orthologs (Fig. S19). Four conserved regions were identified and deleted: the Sox17 NTD, and three distinct regions within the CTD of varying lengths (Fig. 7a: 35 aa, 14 aa, and 20 aa). The mRNA corresponding to each mutant construct was injected into *sox17* CRISPants at the one-cell stage, and their capacity to rescue correct pancreas placement was assessed.

We find that Sox17ΔNTD can rescue pancreas placement in *sox17* CRISPants to a similar degree as FL-Sox17. Both constructs result in a significant increase in CRISPants exhibiting normal right-sided pancreas placement and a significant decrease in embryos showing middle placement, with left-sided placement unaffected compared to *sox17* CRISPants alone (Fig. 7b). This indicates the NTD of Sox17 is largely dispensable for its function in pancreas placement.

Sox17ΔCTD35aa and Sox17ΔCTD14aa mRNA injections lead to a significant decrease in *sox17* CRISPant embryos with a middle-placed pancreas. However, this does not coincide with an increase in normal right-sided placement as seen with FL-Sox17 and Sox17ΔNTD. Instead, there is a significant increase in embryos exhibiting multiple fragmented *insulin*+ domains (Fig. 7b). Sox17ΔCTD20aa mRNA injection leads to a significant increase in CRISPants with normal right-sided placement compared to embryos injected with *sox17* ribonucleoprotein (RNP) alone, suggesting this mutant construct retains some degree of relevant function. However, we also note that we reduction in aberrant pancreas placement is also accompanied by a moderate increase in pancreas fragmentation (Fig. 7b). Overall, all three CTD mutants may have some reduced capability to restore migration of the pancreas to the right, though not to the degree of FL-Sox17 and Sox17ΔNTD. However, the CTD mutants also disrupt pancreas formation, either by driving ectopic specification of pancreas identity or migration defects leading to the fragmented phenotype observed.

*Sox17* ^−/−^ mutant mice exhibit an open body wall and failure to turn from a concave to a convex “foetal” position (Kanai-Azuma et al. 2002; Viotti et al. 2012)—characteristics of mutants with LR patterning defects (Hamada et al. 2002). Given the requirement for Sox17 in LR patterning in mammals and zebrafish, we sought to determine whether mammalian SOX17 has the capacity to induce correct pancreas placement through the injection of h*SOX17* mRNA into *sox17* CRISPants. We found that hSOX17 is unable to rescue pancreas placement. Instead, it leads to a further decrease in right-sided pancreas placement in CRISPants, or even the total absence of the pancreas (Fig. 7b). The aberrant LR phenotype in mouse has been suggested to be largely a secondary effect due to depletion of gut endoderm (Viotti et al. 2012), while zebrafish Sox17 CRISPants do not exhibit loss of endoderm (Fig. S17). Taken together, this indicates that while Sox17 is required for LR asymmetry in mammals and zebrafish, mechanistic variability exists.

Overall, we conclude the Sox17 CTD is required for correct pancreas placement, while the NTD is dispensable. Additionally, our results indicate that peptides in the CTD can influence the formation of an intact endocrine pancreas, although whether this reflects aberrant morphogenesis or specification is unclear.

Taken together, our results reveal the molecular basis for functional divergence between Sox17 and Sox32, highlighting key functional peptides controlling both endoderm and DFC specification, and the establishment of pancreas placement during gut morphogenesis.

## Discussion

**Fig. 8.**
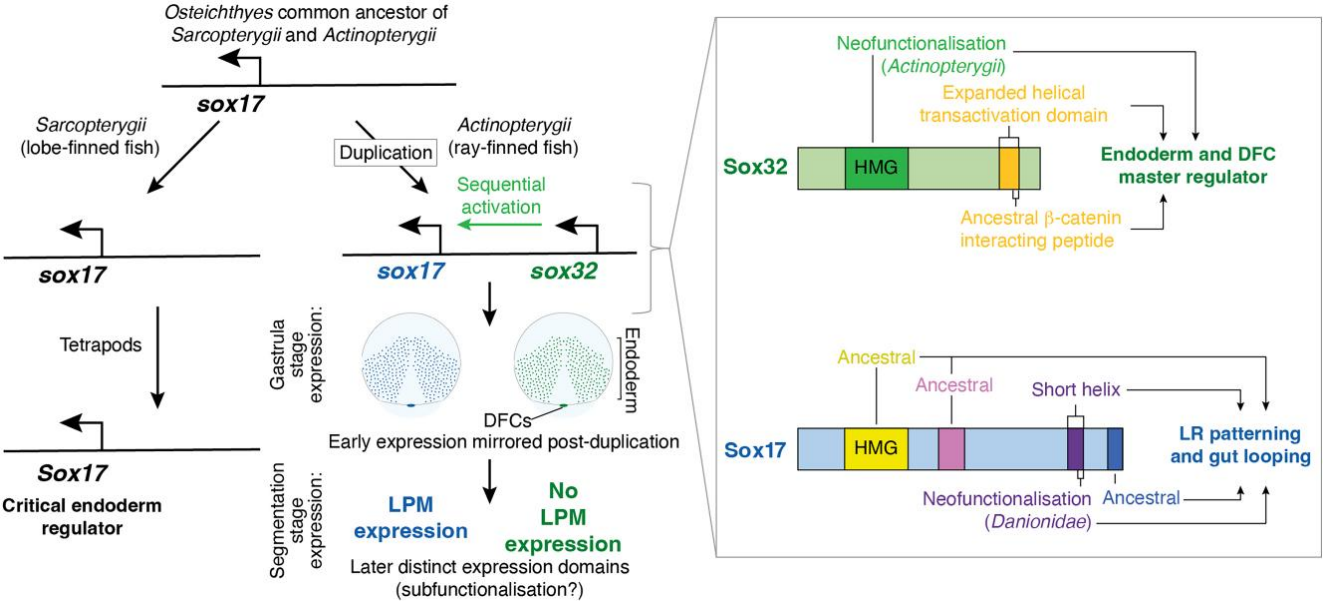
Evolutionary history of *sox17* and *sox32*, including when distinct changes in functional protein-coding domains occurred within the ray-finned fish lineage. Tandem duplication of ancestral *sox17* likely occurred early in the evolution of ray-finned fish after divergence from the common ancestor with lobe-finned fish. Gastrula stage expression of zebrafish *sox17* and *sox32* likely reflects the ancestral gene expression pattern, although in modern zebrafish, there is a temporal offset as Sox32 activates *sox17* (Alexander and Stainier 1999; Kikuchi et al. 2001). Subsequent expression of only *sox17* in LPM (Chung et al. 2011) likely represents subfunctionalization of the ancestral gene, allowing a more complex role for Sox17 in LR patterning. Key protein domains acting in endoderm and DFC formation and LR patterning, and their evolutionary relationships are indicated: coloured boxes in the protein schematics label the DNA-binding HMG domain and various critical CTDs.

A classical view of the fate of duplicated genes that remain functional during evolution is that they either undergo sub- or neofunctionalization (Force et al. 1999; Tasnim et al. 2024). How does the case of Sox17 and Sox32 reflect this? The paralogs’ spatiotemporal expression profiles alone already suggest a complex evolutionary history. On the one hand, the expression domains of *sox32* and *sox17* substantially overlap in the early embryo (pre-segmentation stage, Fig. 8 [Alexander and Stainier 1999; Kikuchi et al. 2001]), and they are similar to *Sox17* expression patterns in other species. However, expression differences after gastrulation stages, when only *sox17* but not *sox32* is expressed in the LPM (Chung et al. 2011), could indicate subfunctionalization through partitioning of the paralogs’ stage- and tissue-specific activities, given known roles of Sox17 in LPM-derived tissues in tetrapods (Lilly et al. 2017; Saba et al. 2019). Yet Sox32's essential functions in the early embryo are clearly not commensurate with mere redundancy after gene duplication, starting with the acquisition of the new regulatory role of activating *sox17*. Thus, here we further consider sub- or neofunctionalization in the context of nuances of protein function (Fig. 8).

### Divergence of the Sox32 HMG Domain Is Critical to Allow Engagement with the Teleost Endoderm GRN

Consistent with previous reports (Kikuchi et al. 2001; Stafford et al. 2006) our analyses indicate that ectopic Sox32 expression induces endodermal cell identity at the expense of other cell types, indicating its ability to change cell fate (Fig. 3g). Remarkably, it also induces a significant number of DFC marker genes, suggesting that Sox32 is both necessary for DFC identity and also substantially sufficient to direct the DFC gene expression program (Fig. 3g).

Our molecular analyses reveal that the ability of Sox32 to orchestrate the endoderm GRN is dependent on differences between the Sox32 and Sox17 HMG domains. Given the greater similarity between Sox17 and hSOX17 HMG domains, we suggest Sox32 diverged after the duplication event from which *sox32* and *sox17* emerged, consistent with our analyses on higher rates of nonsynonymous changes in *sox32* (Figs. S1 and S4, Table S7). This suggests a scenario wherein subfunctionalization has occurred in zebrafish compared to composite SOX17 functions in mammals, with Sox32 adopting the key role in endoderm formation while Sox17 retains other roles such as regulating genes involved in vasculogenesis (Figs. S7 and S9). Functional partitioning thus involves novel sequence divergence, with the acquisition of new regulatory interactions, including Sox32 activation of *sox17*. The evolution of cross-regulation may be a general feature of TF paralog diversification, as documented elsewhere (Cheatle Jarvela et al. 2020; Gurska et al. 2020).

However, the divergence of the Sox32 HMG domain and the inability of hSOX17 to induce endoderm marker expression in zebrafish raise questions about the mechanistic similarities by which these TFs orchestrate endoderm gene expression in their native species. It is possible that the different HMG domains bind different cis-regulatory modules (CRMs) and hence activate different downstream target genes in our experiments, either due to different DNA binding preferences or altered physicochemical interactions with cofactors. However, all Sox TFs are considered to individually bind similar sequence motifs (Kondoh and Kamachi 2010). Furthermore, contact sites between the mouse SOX17 HMG domain and a cis-regulatory enhancer upstream of the *Lama1* transcriptional start site have been mapped (Palasingam et al. 2009) and are highly conserved in Sox32 and Sox17 (Fig. 2d). This may indicate that the Sox32, Sox17, and hSOX17 HMG domains are individually capable of engaging similar DNA sequences. A more likely scenario is that the differences between HMG domains alter the spectrum or configuration of cofactor interactions, impacting co-operative engagement of TF complexes with CRMs. However, it does not necessarily follow that hSOX17 and Sox32 have substantially different cofactors during endoderm formation if orthologous cofactors have undergone parallel evolution. In this scenario, hSOX17 and Sox32 would only be able to interact with cofactors from their native species, which would preclude hSOX17 from inducing endoderm marker expression in zebrafish, even though it is biologically active and affects later development (e.g. Figure 7b). This may also explain why the Sox17 HMG domain cannot substantially engage the endoderm GRN, if key cofactors have evolved to engage Sox32. This scenario highlights a caveat of our comparison of hSOX17 with zebrafish proteins—that parallel cofactor evolution may mask functional similarities of the proteins in their normal endogenous contexts. Equally, differences in codon usage between zebrafish and human may have led to different efficiencies of protein production in our experiments despite the use of equimolar mRNA quantities.

While very few Sox32-interacting TFs have been identified, an interesting candidate that may explain the differences in HMG domain functions is the TF Pou5f3. Pou5f3 is required for Sox32-mediated induction of endoderm fate (Lunde et al. 2004; Reim et al. 2004; Zhao et al. 2013). In mammals, the SOX17 HMG domain interacts with the POU domain of POU5F1 to cooperatively bind CRMs and orchestrate target gene induction (Stefanovic et al. 2009; Ng et al. 2012). While not true orthologs, human *POU5F1* is the closest homologue of zebrafish *pou5f3*. Duplication of an ancestral *Pou5* gene occurred in the last common ancestor of extant cartilaginous fishes and bony fishes, leading to two genes. Subsequent gene loss has led to maintenance of only *pou5f3* in teleosts and *POU5F1* in eutherians, including humans (Frankenberg and Renfree 2013). POU5F1 (also known as OCT3 or OCT4) is known to interact with the HMG domains of many SOX factors (Ng et al. 2012). However, distinctions between HMG domains lead to alterations in POU5F1 engagement, and consequently different SOX-POU5F1 target sequence selection (Ng et al. 2012). Thus, an altered configuration of Sox32-Pou5f3 interactions compared to SOX17-POU5F1 would likely prevent engagement with the same CRMs. Similarly, the requirement for correct partner cooperation in target transcription has been established in human SOX TF function. For example, SOX2 can interact with both OCT3 and OCT1, but only the SOX2–OCT3 complex can promote transcriptional activation of the FGF4 enhancer (Yuan et al. 1995). It is therefore likely that Sox32 complexes have different sequence specificities to Sox17- or hSOX17-containing complexes, either through altered interaction with the same/homologous cofactors or altered cofactor selection. This would lead to different target site selection and distinct functional outcomes for SOX factors that are otherwise capable of binding the same individual consensus sequence. Future analyses should therefore focus on the interacting partners and DNA binding preferences of resulting Sox TF complexes, and whether hSOX17 and Sox32 have demonstrably similar or distinct target CRMs in their native species.

While we hypothesize that divergence of the Sox32 HMG domain may reflect coevolution of common interacting partners, it also may reflect loss, gain, or co-option of alternative cofactors leading to divergence of endoderm gene regulatory programs in ray-finned fish. Related to this, the homeodomain TF Vox acts downstream of BMP and via direct inhibitory interaction with Sox32 in zebrafish to restrict the endodermal domain at the onset of gastrulation (Zhao et al. 2013). Notably, Vox lacks obvious or direct mammalian orthologs. Vox interaction, therefore, likely reflects a key mechanistic distinction in how endoderm specification is achieved by Sox32 and hSOX17. However, since the Vox docking site on Sox32 has not been mapped, it is not presently known whether it is related to differences in the HMG domain. Substantial work is required to determine whether coevolution of common cofactors or altered sets of cofactors explains differences in Sox TF DNA binding. This includes identification of hSOX17, Sox17, and Sox32 interacting partners in their native biological contexts via proteomics approaches or other means, and subsequent analyses of cross-species interaction efficiencies, complex assembly, and CRM engagement. This would be a considerable undertaking but it would shed further light on why Sox32 has undergone faster sequence/structure evolution while seemingly maintaining similar functional requirements in endoderm formation.

Furthermore, while the present study yields valuable insights into key molecular differences underpinning distinct functional capabilities for Sox17 and Sox32, and their ability to induce endoderm and DFC marker genes, it does not reveal their direct target genes during normal development. To gain a fuller understanding of how Sox32 and Sox17 direct endoderm and KV formation at the chromatin level, it will be necessary to profile DNA binding of the endogenous proteins, such as through ChIP-seq, and to induce specific mutations in the endogenous *sox32* and *sox17* genes through genome editing. It will also be necessary to identify their key cofactors and how they combine to dictate target site selection and target gene induction. Such experiments remain challenging due to the endoderm progenitor cells and DFCs being such minor cell populations, and the complexities of precision genome editing. However, our analyses indicate that these are worthy avenues for future study.

### Functional Partition and Divergence Through Alteration of C-terminal Helices

A key distinction between these endoderm-relevant SoxF factors is a putative CTD helix, which is notably longer in Sox32 than Sox17 (Fig. 2a-c). The 25-aa high-confidence prediction in Sox32 (Fig. 2a) encompasses a short peptide (EFDQYL) that deviates from hSOX17 by only a single amino acid of similar properties (EFEQYL). Importantly, this EF(D/E)QYL motif was first identified in *Xenopus*, where Sox17α shows 100% sequence identity to hSOX17 and Sox17β to Sox32, with both peptides critical to Sox17α/β interaction with the Wnt pathway effector β-catenin (Sinner et al. 2004). Additionally, *Xenopus* Sox17α/β and hSOX17 co-occupy enhancers with β-catenin, resulting in activation of endoderm-specific genes and repression of mesoectodermal genes (Mukherjee et al. 2020, 2022). However, zebrafish Sox17 exhibits alterations in this peptide, including replacement of the central polar uncharged glutamine (Q) residue with a positively charged histidine (H) residue, and also substitution of the polar amino acid tyrosine (Y) for nonpolar cysteine (C) (Fig. 2e). It is therefore likely that Sox32 has retained the ancestral function of recruiting β-catenin to activate endoderm-specific genes, while divergence of this peptide in Sox17 has led to neofunctionalization. This is supported by our observation that both Sox32 and Sox17 require their own individual peptide sequences (EFDQYLN and EFEHCLS, respectively, as tested) for the induction of their distinct target genes. Thus, the function of Sox32 in directing the endoderm GRN relies on divergence of its HMG domain and retention of the C-terminal β-catenin interacting peptide, while alterations to this C-terminal peptide in Sox17 likely led to altered target gene selection and consequent functional diversification. It is notable that the EFEQYL β-catenin binding sequence is highly conserved in both Sox32 and Sox17 in the majority of fish species, with the alterations identified in zebrafish Sox17 seemingly restricted to a subset of families within the order *Cypriniformes* (Figs. S12 to S13). While functional divergence driven by changes in the Sox32 HMG domain is therefore likely to be ancient within the evolution of ray-finned fish, the functional differences we identify between zebrafish Sox32 and Sox17 C-terminal peptides likely reflect much more recent neofunctionalization of Sox17 (Fig. 8).

The 25-aa CTD helical domain in Sox32 contains 18 aa upstream of the β-catenin interacting peptide. Our analyses (via deletion of a 23-aa peptide encompassing the 18-aa helix and five upstream amino acids) indicates that this broader helical domain is also essential for Sox32-mediated induction of endoderm and DFC target genes (Fig. 4). Additionally, though Sox17 requires its divergent EFEHCLS peptide for induction of its target genes, substitution for the Sox32 25-aa CTD helical domain nevertheless leads to potent induction of Sox17 (though not Sox32) target genes (Fig. 5d-e). We therefore hypothesize that the Sox32 CTD helix is a potent transactivation domain. Interestingly, C-terminal helices in FoxA factors have been shown to be critical for gene activation, facilitating binding to histone octamers, leading to nucleosome eviction and increased chromatin accessibility (Pani et al. 1992; Iwafuchi et al. 2020). It would be interesting to determine whether the 25-aa CTD helix in Sox32 similarly confers pioneer TF activity.

Future investigation of Sox17 and Sox32 CTD helices should therefore focus on their common and distinct binding partners, with histones and β-catenin as interesting candidates to explore potential roles as pioneer factors or transducers of Wnt signaling. To determine whether and how differences in CTD helices dictate differences in target gene specificity, it would also be advantageous to perform precision editing of the endogenous *sox17* and *sox32* genes and to profile changes in genomic binding of the resulting gene products.

### Sox17 Potentially Has Multiple Roles in Establishing Organ Asymmetry

Our analyses of G0 CRISPants suggest Sox17 is necessary for correct unilateral Nodal (*spaw*) expression, brain asymmetry, heart looping, and pancreas placement (Fig. 6). Furthermore, we have shown that co-injection with exogenous *sox17* mRNA is sufficient to rescue abnormal pancreas placement phenotypes in Sox17 CRISPants, consistent with previous experiments with antisense MOs (Aamar and Dawid 2010). However, exogenous *sox17* mRNA is not sufficient to rescue abnormal Nodal expression, heart looping, or brain asymmetry. This tantalisingly suggests Sox17 may have distinct roles in different cell types to establish organ asymmetry, as we discuss below.

The process of zebrafish heart morphogenesis and looping has been extensively studied and reviewed (Bakkers 2011). While we have shown that an intact *sox17* locus is necessary for correct zebrafish heart looping, the lack of phenotypic rescue by exogenous *sox17* mRNA cannot be attributed to deleterious effects of exogenous *sox17* mRNA (Fig. S16) or a secondary consequence of an endodermal phenotype (Fig. S19). Our RNA-seq analyses show Sox17-induced genes are enriched for heart-related anatomical terms, including endocardium and ventricular myocardium (Fig. S8). Additionally, we have observed *sox17:EGFP* reporter expression in the heart, including in the endocardium, based on co-expression with *kdrl:mCherry* (Fig. S9A, Video S1). These cells arise from precursors in the LPM, which are *sox17*+ (Zhong et al. 2001; Chung et al. 2011). *Sox17* is expressed in mouse endocardium, where it is required for proper heart morphogenesis (Saba et al. 2019). Considering we find *sox17:EGFP*+ cells within the zebrafish heart (Fig. S9), a conserved role within the endocardium controlling heart morphogenesis is plausible.

Sox17 is also expressed within KV (Schneider et al. 2008; Chung et al. 2011), and MO knockdown of Sox17 leads to abnormality in KV morphogenesis and function via defective ciliogenesis (Aamar and Dawid 2010). The perturbation of cilia function results in aberrant *spaw* expression within the LPM (Kramer-Zucker et al. 2005). We show that Sox17 CRISPants exhibit aberrant *spaw* expression in the LPM, and this cannot be restored to normal left-sided *spaw* expression through co-injection of exogenous *sox17* mRNA (Fig. S18). This suggests we are not able to rescue KV function with exogenous *sox17* mRNA, leading to an inability to rescue correct brain asymmetry and heart looping. An explanation may be the off-target disruption of another gene acting in KV function. However, this seems unlikely given that MO knockdown of Sox17 also leads to KV defects (Aamar and Dawid 2010). Alternatively, since KV typically consists of ∼50 cells (Gokey et al. 2016)—a tiny proportion of the embryo—it is possible that the failure to rescue left-sided *spaw* expression is due to a lack of inheritance of exogenous *sox17* mRNA by sufficient KV cells, as *sox17* OE itself does not lead to LR defects (Fig. S16).

However, the failure to rescue KV function (and thus brain asymmetry and heart looping) makes the rescue of pancreas placement even more intriguing. As well as the KV function, visceral organ laterality and therefore pancreas placement is also determined by LPM migration, which pushes the developing pancreas to the right of the midline due to left-sided Nodal signaling (Horne-Badovinac et al. 2003). Alterations in Nodal signaling within the LPM randomize the pattern of LPM migration, resulting in aberrant pancreas placement (Horne-Badovinac et al. 2003). Remarkably, however, exogenous *sox17* mRNA can restore gut looping in Sox17 CRISPants as indicated by a significant reduction in midline-placed pancreas, even though it cannot rescue KV function (Fig. 6b). Since *sox17* is expressed in LPM (Chung et al. 2011), this suggests that LPM migration is compromised in Sox17 CRISPants, but can be restored through exogenous *sox17* mRNA injection. It seems likely that observation of a fragmented pancreas on either side of the midline after mutant *sox17* mRNA injection also reflects effects on LPM migration. However, this is not sufficient to explain the fragmentation itself.

The fragmented pancreas phenotype we observe in embryos expressing mutant forms of *sox17* is somewhat reminiscent of both *miR-375* loss-of-function and Cdx TF deficiency. Insulin (*ins*) expression in the presumptive pancreatic islet is first apparent in a disparate subset of pancreatic progenitor cells situated at the midline at the 12-somite stage (15 hpf). As these pancreatic β cells mature, they migrate to form a single cluster within the pancreatic domain by 24 hpf (Biemar et al. 2001; Chung and Stainier 2008). In the absence of *miR-375* function, the *ins*-expressing pancreatic islet cluster is intact at 24 hpf but becomes dispersed at later stages (Kloosterman et al. 2007). Conversely, combined loss of Cdx4 and Cdx1a function leads to an expanded pancreatic domain, which in turn leads to delayed convergence of *ins*-expressing cells and thus a fragmented phenotype at 24 hpf (Kinkel et al. 2008). The fragmentation phenotype that we observe could therefore reflect aberrant migration of *ins*-expressing cells or an expanded pancreatic domain. It is tempting to speculate that alterations in *miR-375* and/or *Cdx* TF expression, combined with the LR patterning defects in Sox17 CRISPants are responsible for the fragmented *ins* expression patterns we observe along the anterior-posterior and LR axes (Figs. 6 and 7). Alternatively, the mutant forms of Sox17 may be influencing other factors controlling pancreatic β-cell migration or the boundaries of the pancreatic domain.

In sum, our data confirms that Sox17 controls LR asymmetry through roles in KV and suggests new roles for Sox17 in gut looping and possibly also in heart looping through expression in the endocardium. Our analyses show that multiple peptides conserved among Sox17 orthologs are required for rescue of the pancreas placement defect in Sox17 CRISPants. Strikingly, amongst these is the Sox17CTDΔ14 aa deletion, which encompasses the EFEHCLS sequence noted to have diverged from the ancestral β-catenin interacting peptide. It is therefore possible that this divergence and likely neofunctionalization of Sox17 is key to its role in controlling gut looping in zebrafish.

An interesting outstanding question is whether either Sox32 or hSOX17 expressed from the *sox17* promoter could rescue the loss of Sox17 in LR patterning. It is unlikely that Sox32 is capable of fulfilling the role of Sox17 since it acts upstream of *sox17* and is therefore still expressed in endoderm and DFCs in Sox17 CRISPants. However, zebrafish embryos expressing *hSOX17* from the *sox17* promoter would be worthy of production and future analysis.

Overall, our results indicate that evolutionary alterations in the HMG domain of Sox32, combined with the presence of a CTD helical domain, including retention of a β-catenin interacting peptide, are key to its orchestration of the endoderm and DFC GRNs. While hSOX17 clearly cannot engage the zebrafish endoderm GRN in the context of these experiments, it would be interesting to explore whether Sox32 and hSOX17 have similar target genes and cofactors within their native species. In contrast to Sox32, alterations in the CTD of Sox17 have driven functional divergence. This alters target gene specificity in the early embryo and may be related to a new function for Sox17 in controlling gut looping to establish visceral organ asymmetry, which warrants further in-depth investigation.

## Materials and Methods

### Animals

AB, *Tg(sox17:EGFP)^ha01Tg^* and *Tg(kdrl:mCherry^S896^,gata1a:dsRed^sd2tg^)* fish were reared as described (Westerfield 2000; Traver et al. 2003; Chi et al. 2008; Mizoguchi et al. 2008). All zebrafish studies were conducted in compliance with the United Kingdom Animals (Scientific Procedures) Act 1986, licensed by the UK Home Office, and implemented by The University of Warwick.

### Confocal Microscopy

48 hpf zebrafish embryos were anesthetized with 0.168% (w/v) of ethyl 3-aminobenzoate methanesulfonate, prior to mounting in 1% lateral view agarose moulds. Moulds were generated from a 3D stamp as designed by Kleinhans and Lecaudey (2019). To ensure high-quality imaging, embryos were treated before 24 hpf with 0.003% (w/v) 1-phenyl-2-thiourea to prevent pigmentation (Karlsson et al. 2001). Imaging was carried out on a Zeiss LSM 980 with Airyscan 2.

### Cloning for In Vitro Production of mRNAs

pCS2 + *hSOX17* was cloned from PB-TRE3G-SOX17 (Miller et al. 2018) using XhoI and XbaI restriction sites. PB-TRE3G-SOX17 was a gift from David Vereide (Addgene plasmid # 104541; http://n2t.net/addgene:104541; RRID:Addgene_104541).

*Sox17* and *sox32* deletion and domain switch constructs (Table S13) were generated using pCS2 + *sox17* and pCS2 + *myc-sox32* by PCR amplification using Q5 polymerase (NEB) with the described primers (Table S14), followed by re-circularization or insertion using T4 ligase (Invitrogen). All constructs were generated using blunt-end cloning, except for Sox17Δ7aa + Sox32 25aa and Sox32ΔHMG + Sox17 HMG, where NEBuilder HiFi DNA Assembly (NEB), with Sox17Δ7aa + Sox32 25aa as a gBlock (Integrated DNA Technologies), was used.

### mRNA In Vitro Transcription

Capped mRNA was synthesized from pCS2+ plasmids containing genes of interest. Linearization with NotI was conducted, followed by transcription using SP6 RNA polymerase as previously described (Talbot et al. 2022).

### RNP Complex Production

To knockdown, *sox17* RNP complexes were generated using a pool of three guide RNAs and Cas9 protein (Integrated DNA Technologies) as described (Essner 2016). Guide RNAs that do not target the zebrafish genome were used to generate a negative control RNP. Guide RNAs were generated from specific crRNAs (Table S15) and standard tracrRNA (Integrated DNA Technologies) as described (Essner 2016). crRNAs against *sox17* were predesigned by Integrated DNA Technologies and selected based on predicted on-target and off-target scores from both Integrated DNA Technologies and CHOPCHOP (Labun et al. 2019).

### Microinjection

Equimolar amounts of mRNA (calculated based on relative mRNA length) were injected at the one-cell stage. For interspecies RNA-seq, each embryo was injected with the following amount: *sox32*—150pg, h*SOX17*—141pg, and *sox17*—175pg. For HMG Switch RNA-seq, each embryo was injected with the following amount: *sox32*—150pg, *sox32*ΔHMG—121.5pg, and *sox32*ΔHMG + *sox17*HMG—148.5pg. For *sox32* mutant qPCR, each embryo was injected with the following amount: *sox32*—150pg, -*sox32*ΔNTD—127.5pg, *sox32*ΔHMG—121.5pg, *sox32*ΔCTD23aa—142.5pg, and *sox32*ΔCTD7aa—147pg. For CTD switch qPCR, each embryo was injected with the following amount: *sox17*—150pg, *sox17*Δ7aa—147pg, *sox32*—128 ng, *sox32*Δ7aa + *sox17-*7aa—128pg, *sox17*Δ7aa + *sox32-*7aa—150pg, and *sox17*Δ7aa + *sox32-*25aa—155pg. For Sox17 functional dissection, each embryo was injected with the following amount of mRNA: *sox17*—150pg, *sox17*ΔNTD—134pg, *sox17*ΔCTD35aa—139pg, *sox17*ΔCTD14aa—145pg, *sox17*ΔCTD20aa—143pg, and h*SOX17*—108pg. For OE experiments, mRNA was injected in a volume of 1 nL. To produce CRISPant embryos, RNPs were injected in a volume of 3 nL. For experiments involving combined CRISPR RNP and mRNA injection, RNPs and mRNAs were injected in a combined volume of 4 nL.

### Whole-Mount In Situ Hybridization (WISH)

WISH of zebrafish embryos was carried out as described (Thisse and Thisse 2008). Antisense RNA probes for *myl7* (Yelon et al. 1999), *insulin* (Milewski et al. 1998), *vgll4l* (Nelson et al. 2014), *sox17* (Alexander and Stainier 1999), and *foxa2* (Odenthal and Nüsslein-Volhard 1998) were synthesized as described. *Ktcd12.1* RNA probe was generated from a PCR product using primers: F—CTGCCGGACTATTTTCCAGAG, R—TAATACGACTCACTATAGGGAGCTGCACGCGACCATCT, and transcribed using T7 polymerase (Promega). Categorical scoring on randomized samples was completed blinded.

### Total RNA Extraction

Embryos were lysed at 6 hpf via the addition of 600 µL buffer RLT and DTT (2% v/v) to at least fifty embryos per condition, with initial disruption using a plastic pestle. Further disruption was carried out using QIAshredder columns (QIAGEN), and RNA was extracted using the RNeasy Mini Kit (QIAGEN) with on-column DNase treatment (QIAGEN) as per the manufacturer's protocol.

### qPCR

cDNA was generated from 400 ng of total RNA using Random Hexamers (Promega) and Ultrascript Reverse Transcriptase (PCR Bio) as per the manufacturer's protocol. qPCR was carried out using relevant primers (Table S16) and Power SYBR Green Master Mix (Thermofisher) on the Stratagene Mx3005P (Agilent Technologies). Raw Ct values were extracted using MxPro software and analyzed using the ΔΔCt method (Livak and Schmittgen 2001), with normalization to *18S* rRNA (McCurley and Callard 2008). Statistical differences were inferred using Student’s *t*-test with significance set *at P* < 0.05.

### RNA-Sequencing Library Preparation and Analysis

Total RNA was sent to the Cologne Center for Genomics for library preparation (Illumina TruSeq Stranded mRNA) and sequencing on the Illumina NovaSeq 6000 platform with a read length of 101 bp.

Quality control of raw data was performed using FastQC. Reads were mapped to the danRer11 zebrafish genome and counts per gene (Ensembl version 97) generated using STAR with default settings (Dobin et al. 2013). Differential expression was identified through adjusted *P*-value < 0.05 using DESeq2 (1.36.0) (Love et al. 2014) in R (4.2.0). To identify whether different TFs illicit similar or different effects on the transcriptome, upregulated and downregulated DEGs were separately overlapped to identify whether TFs induce or repress similar genes. Statistical testing to identify whether the overlap in genes was significantly more/less than expected by chance was conducted using Fisher's exact test (phyper in R). This analysis used a gene universe size defined as all genes detected in RNA-seq, where samples in all conditions showed counts > 0. Heatmaps were generated on median normalized counts per condition using pheatmap (1.0.12) with “scale = row” option to display deviation in expression from the average expression of each gene. GO and anatomy term enrichment analysis was carried out on fishEnrichR (Chen et al. 2013; Kuleshov et al. 2016) using autoRIF *Z*-score, with significantly enriched terms defined by an adjusted *P*-value < 0.05.

To infer how TFs influence cell fate, Gene Set Enrichment Analysis (GSEA) was carried out using published scRNA-sequencing data (Wagner et al. 2018) as cell identity markers using GSEA (4.3.2) (Mootha et al. 2003; Subramanian et al. 2005).

### Conservation Analysis and Phylogeny

Protein and mRNA sequences used for phylogenetic and alignment analyses were obtained from Ensembl, GenBank, and Uniprot databases, as indicated in figure legends and Table S1. Peptide sequences were aligned using MAFFT v7.526 (Katoh and Standley 2013) with the option “--auto.” A maximum likelihood phylogenetic tree was constructed using IQ-TREE v2.3.6 (Minh et al. 2020) with the best-fitting model selected via option “-m MFP,” and branch support was assessed with 1,000 ultrafast bootstrap replicates with option “-B 1000.” The resulting tree was visualized using FigTree v1.4.4 (http://tree.bio.ed.ac.uk/software/figtree). GeneRax v2.1.3 (Morel et al. 2020) was used to reconcile the gene trees with the species tree using the options “--rec-model UndatedDL --geneSearchStrategy SPR.” The reconciled gene trees were then visualized with ThirdKind v3.13.1 (Penel et al. 2022).

For HMG domains, maximum likelihood phylogenetic trees were constructed with default parameters at phylogeny.fr, using MUSCLE v3.8.31 for the alignment, PhyML v3.1/3.0 aLRT for tree construction, and TreeDyn 198.3 for tree rendering (Chevenet et al. 2006; Dereeper et al. 2008). Differences in rates of nonsynonymous coding sequence changes were determined using the Relative Rate Test tool RRTree v.1.1.11 via the Phylemon 2.0 suite (Robinson-Rechavi and Huchon 2000; Sanchez et al. 2011). cDNA sequences encoding HMG domains used for the analyses are indicated in Table S7. cDNA sequences were categorized into lineages in RRTree according to the relationships in Fig. S1. *Ciona intestinalis* or elephant shark cDNA sequences were assigned as outgroups, as indicated in Results.

For the identification of peptides conserved between Sox32 or Sox17 orthologs for deletion analysis, protein sequences were obtained from UniProt (The UniProt 2023) (Table S17), and alignments were conducted using Clustal Omega in JalView (Sievers et al. 2011; Sievers and Higgins 2018). Jalview uses multiple sequence alignments according to Analysis of Multiply Aligned Sequences (AMAS) (Livingstone and Barton 1993) to calculate conservation levels. Conserved sequences were compared to 3D structures obtained from AlphaFold: Sox32 (Q90Z46), Sox17 (Q5PQZ5), and hSOX17 (Q9H6I2) (Jumper et al. 2021; Varadi et al. 2022).

## Supplementary Material

evaf213_Supplementary_Data

## Data Availability

All RNA-seq data have been submitted to NCBI Gene Expression Omnibus under accession number GSE274063.

## References

[evaf213-B1] Aamar E, Dawid IB. Sox17 and chordin are required for formation of Kupffer's vesicle and left-right asymmetry determination in zebrafish. Dev Dyn. 2010:239:2980–2988. 10.1002/dvdy.22431. 20925124 PMC3090657

[evaf213-B2] Agarwal R, Varghese R, Jesudian V, Moses J. The heterotaxy syndrome: associated congenital heart defects and management. Indian J Thorac Cardiovasc Surg. 2021:37:67–81. 10.1007/s12055-020-00935-y. 33603285 PMC7859150

[evaf213-B3] Aiello VD, et al The nomenclature, definition and classification of cardiac structures in the setting of heterotaxy. Cardiol Young. 2007:17:1–28. 10.1017/S1047951107001138. 18039396

[evaf213-B4] Alexander J, Rothenberg M, Henry GL, Stainier DYR. Casanova plays an early and essential role in endoderm formation in zebrafish. Dev Biol. 1999:215:343–357. 10.1006/dbio.1999.9441. 10545242

[evaf213-B5] Alexander J, Stainier DY. A molecular pathway leading to endoderm formation in zebrafish. Curr Biol. 1999:9:1147–1157. 10.1016/S0960-9822(00)80016-0. 10531029

[evaf213-B6] Angelozzi M, Lefebvre V. SOXopathies: growing family of developmental disorders due to SOX mutations. Trends Genet. 2019:35:658–671. 10.1016/j.tig.2019.06.003. 31288943 PMC6956857

[evaf213-B7] Aoki TO, et al Molecular integration of casanova in the Nodal signalling pathway controlling endoderm formation. Development. 2002:129:275–286. 10.1242/dev.129.2.275. 11807021

[evaf213-B8] Argenton F, Zecchin E, Bortolussi M. Early appearance of pancreatic hormone-expressing cells in the zebrafish embryo. Mech Dev. 1999:87:217–221. 10.1016/S0925-4773(99)00151-3. 10495291

[evaf213-B9] Bakkers J . Zebrafish as a model to study cardiac development and human cardiac disease. Cardiovasc Res. 2011:91:279–288. 10.1093/cvr/cvr098. 21602174 PMC3125074

[evaf213-B10] Bianco IH, Wilson SW. The habenular nuclei: a conserved asymmetric relay station in the vertebrate brain. Philosoph Trans R Soc B: Biol Sci. 2008:364:1005–1020. 10.1098/rstb.2008.0213. PMC2666075 19064356

[evaf213-B11] Biemar F, et al Pancreas development in zebrafish: early dispersed appearance of endocrine hormone expressing cells and their convergence to form the definitive islet. Dev Biol. 2001:230:189–203. 10.1006/dbio.2000.0103. 11161572

[evaf213-B12] Bowles J, Schepers G, Koopman P. Phylogeny of the SOX family of developmental transcription factors based on sequence and structural indicators. Dev Biol. 2000:227:239–255. 10.1006/dbio.2000.9883. 11071752

[evaf213-B13] Braasch I, et al The spotted gar genome illuminates vertebrate evolution and facilitates human-teleost comparisons. Nat Genet. 2016:48:427–437. 10.1038/ng.3526. 26950095 PMC4817229

[evaf213-B14] Cheatle Jarvela AM, Trelstad CS, Pick L. Regulatory gene function handoff allows essential gene loss in mosquitoes. Commun Biol. 2020:3:540. 10.1038/s42003-020-01203-w. 32999445 PMC7528073

[evaf213-B15] Chen EY, et al Enrichr: interactive and collaborative HTML5 gene list enrichment analysis tool. BMC Bioinformatics. 2013:14:128. 10.1186/1471-2105-14-128. 23586463 PMC3637064

[evaf213-B16] Chevenet F, Brun C, Banuls AL, Jacq B, Christen R. TreeDyn: towards dynamic graphics and annotations for analyses of trees. BMC Bioinformatics. 2006:7:439. 10.1186/1471-2105-7-439. 17032440 PMC1615880

[evaf213-B17] Chi NC, et al Foxn4 directly regulates tbx2b expression and atrioventricular canal formation. Genes Dev. 2008:22:734–739. 10.1101/gad.1629408. 18347092 PMC2275426

[evaf213-B18] Chung MIS, Ma ACH, Fung T-K, Leung AYH. Characterization of Sry-related HMG box group F genes in zebrafish hematopoiesis. Exp Hematol. 2011:39:986–998.e985. 10.1016/j.exphem.2011.06.010. 21726513

[evaf213-B19] Chung WS, Stainier DY. Intra-endodermal interactions are required for pancreatic beta cell induction. Dev Cell. 2008:14:582–593. 10.1016/j.devcel.2008.02.012. 18410733 PMC2396532

[evaf213-B20] Concha ML, Wilson SW. Asymmetry in the epithalamus of vertebrates. J Anat. 2001:199:63–84. 10.1046/j.1469-7580.2001.19910063.x. 11523830 PMC1594988

[evaf213-B21] Cooper MS, D'Amico LA. A cluster of noninvoluting endocytic cells at the margin of the zebrafish blastoderm marks the site of embryonic shield formation. Dev Biol. 1996:180:184–198. 10.1006/dbio.1996.0294. 8948584

[evaf213-B22] Dereeper A, et al Phylogeny.fr: robust phylogenetic analysis for the non-specialist. Nucleic Acids Res. 2008:36:W465–W469. 10.1093/nar/gkn180. 18424797 PMC2447785

[evaf213-B23] Dickmeis T, et al A crucial component of the endoderm formation pathway, CASANOVA, is encoded by a novel sox-related gene. Genes Dev. 2001:15:1487–1492. 10.1101/gad.196901. 11410529 PMC312720

[evaf213-B24] Dobin A, et al STAR: ultrafast universal RNA-seq aligner. Bioinformatics (Oxford, England). 2013:29:15–21. 10.1093/bioinformatics/bts635. 23104886 PMC3530905

[evaf213-B25] Essner JJ . Zebrafish embryo microinjection: Ribonucleoprotein delivery using the Alt-RTM CRISPR-Cas9 System. Integrated DNA Technologies. 2016.

[evaf213-B26] Essner JJ, Amack JD, Nyholm MK, Harris EB, Yost HJ. Kupffer's vesicle is a ciliated organ of asymmetry in the zebrafish embryo that initiates left-right development of the brain, heart and gut. Development. 2005:132:1247.LP–1241260. 10.1242/dev.01663. 15716348

[evaf213-B27] Figiel DM, Elsayed R, Nelson AC. Investigating the molecular guts of endoderm formation using zebrafish. Brief Funct Genomics; 2021. 10.1093/bfgp/elab013 33754635

[evaf213-B28] Force A, et al Preservation of duplicate genes by complementary, degenerative mutations. Genetics. 1999:151:1531–1545. 10.1093/genetics/151.4.1531. 10101175 PMC1460548

[evaf213-B29] Frankenberg S, Renfree MB. On the origin of POU5F1. BMC Biol. 2013:11:56. 10.1186/1741-7007-11-56. 23659605 PMC3665618

[evaf213-B30] Gamse JT, et al Directional asymmetry of the zebrafish epithalamus guides dorsoventral innervation of the midbrain target. Development. 2005:132:4869–4881. 10.1242/dev.02046. 16207761

[evaf213-B31] Gamse JT, Thisse C, Thisse B, Halpern ME. The parapineal mediates left-right asymmetry in the zebrafish diencephalon. Development. 2003:130:1059–1068. 10.1242/dev.00270. 12571098

[evaf213-B32] Gokey JJ, Ji Y, Tay HG, Litts B, Amack JD. Kupffer's vesicle size threshold for robust left-right patterning of the zebrafish embryo. Dev Dyn. 2016:245:22–33. 10.1002/dvdy.24355. 26442502 PMC5434515

[evaf213-B33] Gubbay J, et al A gene mapping to the sex-determining region of the mouse Y chromosome is a member of a novel family of embryonically expressed genes. Nature. 1990:346:245–250. 10.1038/346245a0. 2374589

[evaf213-B34] Gurska D, Vargas Jentzsch IM, Panfilio KA. Unexpected mutual regulation underlies paralogue functional diversification and promotes epithelial tissue maturation in Tribolium. Commun Biol. 2020:3:552. 10.1038/s42003-020-01250-3. 33020571 PMC7536231

[evaf213-B35] Hamada H, Meno C, Watanabe D, Saijoh Y. Establishment of vertebrate left–right asymmetry. Nat Rev Genet. 2002:3:103–113. 10.1038/nrg732. 11836504

[evaf213-B36] Horne-Badovinac S, Rebagliati M, Stainier DYR. A cellular framework for gut-looping morphogenesis in zebrafish. Science. 2003:302:662–665. 10.1126/science.1085397. 14576439

[evaf213-B37] Iwafuchi M, et al Gene network transitions in embryos depend upon interactions between a pioneer transcription factor and core histones. Nat Genet. 2020:52:418–427. 10.1038/s41588-020-0591-8. 32203463 PMC7901023

[evaf213-B38] Jumper J, et al Highly accurate protein structure prediction with AlphaFold. Nature. 2021:596:583–589. 10.1038/s41586-021-03819-2. 34265844 PMC8371605

[evaf213-B39] Kanai-Azuma M, et al Depletion of definitive gut endoderm in Sox17-null mutant mice. Development. 2002:129:2367–2379. 10.1242/dev.129.10.2367. 11973269

[evaf213-B40] Karlsson J, von Hofsten J, Olsson P-E. Generating transparent zebrafish: a refined method to improve detection of gene expression during embryonic development. Marine Biotechnology. 2001:3:522–527. 10.1007/s1012601-0053-4. 14961324

[evaf213-B41] Katoh K, Standley DM. MAFFT multiple sequence alignment software version 7: improvements in performance and usability. Mol Biol Evol. 2013:30:772–780. 10.1093/molbev/mst010. 23329690 PMC3603318

[evaf213-B42] Kikuchi Y, et al The zebrafish bonnie and clyde gene encodes a Mix family homeodomain protein that regulates the generation of endodermal precursors. Genes and Development. 2000:14:1279–1289. 10.1101/gad.14.10.1279. 10817762 PMC316618

[evaf213-B43] Kikuchi Y, et al Casanova encodes a novel Sox-related protein necessary and sufficient for early endoderm formation in zebrafish. Genes and Development. 2001:5:1493–1505 10.1101/gad.892301. PMC312713 11410530

[evaf213-B44] Kinkel MD, Eames SC, Alonzo MR, Prince VE. Cdx4 is required in the endoderm to localize the pancreas and limit beta-cell number. Development. 2008:135:919–929. 10.1242/dev.010660. 18234725

[evaf213-B45] Kleinhans DS, Lecaudey V. Standardized mounting method of (zebrafish) embryos using a 3D-printed stamp for high-content, semi-automated confocal imaging. BMC Biotechnol. 2019:19:68. 10.1186/s12896-019-0558-y. 31640669 PMC6805687

[evaf213-B46] Kloosterman WP, Lagendijk AK, Ketting RF, Moulton JD, Plasterk RH. Targeted inhibition of miRNA maturation with morpholinos reveals a role for miR-375 in pancreatic islet development. PLoS Biol. 2007:5:e203. 10.1371/journal.pbio.0050203. 17676975 PMC1925136

[evaf213-B47] Kondoh H, Kamachi Y. SOX-partner code for cell specification: regulatory target selection and underlying molecular mechanisms. Int J Biochem Cell Biol. 2010:42:391–399. 10.1016/j.biocel.2009.09.003. 19747562

[evaf213-B48] Kramer-Zucker AG, et al Cilia-driven fluid flow in the zebrafish pronephros, brain and Kupffer's vesicle is required for normal organogenesis. Development. 2005:132:1907.LP–1901921. 10.1242/dev.01772. 15790966

[evaf213-B49] Kuleshov MV, et al Enrichr: a comprehensive gene set enrichment analysis web server 2016 update. Nucleic Acids Res. 2016:44:W90–W97. 10.1093/nar/gkw377. 27141961 PMC4987924

[evaf213-B50] Labun K, et al CHOPCHOP v3: expanding the CRISPR web toolbox beyond genome editing. Nucleic Acids Res. 2019:47:W171–W174. 10.1093/nar/gkz365. 31106371 PMC6602426

[evaf213-B51] Lilly AJ, Lacaud G, Kouskoff V. SOXF transcription factors in cardiovascular development. Semin Cell Dev Biol. 2017:63:50–57. 10.1016/j.semcdb.2016.07.021. 27470491

[evaf213-B52] Livak KJ, Schmittgen TD. Analysis of relative gene expression data using real-time quantitative PCR and the 2(-ΔΔC(T)) method. Methods (San Diego, Calif). 2001:25:402–408. 11846609 10.1006/meth.2001.1262

[evaf213-B53] Livingstone CD, Barton GJ. Protein sequence alignments: a strategy for the hierarchical analysis of residue conservation. Comput Appl Biosci. 1993:9:745–756. 10.1093/bioinformatics/9.6.745. 8143162

[evaf213-B54] Long S, Ahmad N, Rebagliati M. The zebrafish nodal-related gene southpaw is required for visceral and diencephalic left-right asymmetry. Development (Cambridge, England). 2003:130:2303–2316. 12702646 10.1242/dev.00436

[evaf213-B55] Love MI, Huber W, Anders S. Moderated estimation of fold change and dispersion for RNA-seq data with DESeq2. Genome Biol. 2014:15:550. 10.1186/s13059-014-0550-8. 25516281 PMC4302049

[evaf213-B56] Lunde K, Belting HG, Driever W. 2004. Zebrafish pou5f1/pou2, homolog of mammalian Oct4, functions in the endoderm specification cascade. Curr Biol. 2004;14(1):48–55 10.1016/j.cub.2003.11.022. 14711414

[evaf213-B57] Matsui T, et al Redundant roles of Sox17 and Sox18 in postnatal angiogenesis in mice. J Cell Sci. 2006:119:3513–3526. 10.1242/jcs.03081. 16895970

[evaf213-B58] McCurley AT, Callard GV. Characterization of housekeeping genes in zebrafish: male-female differences and effects of tissue type, developmental stage and chemical treatment. BMC Mol Biol. 2008:9:102. 10.1186/1471-2199-9-102. 19014500 PMC2588455

[evaf213-B59] McDonald Angela CH, Biechele S, Rossant J, Stanford William L. Sox17-mediated XEN cell conversion identifies dynamic networks controlling cell-fate decisions in embryo-derived stem cells. Cell Rep. 2014:9:780–793. 10.1016/j.celrep.2014.09.026. 25373912

[evaf213-B60] Melby AE, Warga RM, Kimmel CB. Specification of cell fates at the dorsal margin of the zebrafish gastrula. Development. 1996:122:2225.LP–2222237. 10.1242/dev.122.7.2225. 8681803

[evaf213-B61] Milewski WM, Duguay SJ, Chan SJ, Steiner DF. Conservation of PDX-1 structure, function, and expression in zebrafish. Endocrinology. 1998:139:1440–1449. 10.1210/endo.139.3.5768. 9492081

[evaf213-B62] Miller AZ, et al Expandable arterial endothelial precursors from human CD34+ cells differ in their proclivity to undergo an endothelial-to-mesenchymal transition. Stem Cell Reports. 2018:10:73–86. 10.1016/j.stemcr.2017.12.011. 29320761 PMC5769011

[evaf213-B63] Minh BQ, et al IQ-TREE 2: new models and efficient methods for phylogenetic inference in the genomic era. Mol Biol Evol. 2020:37:1530–1534. 10.1093/molbev/msaa015. 32011700 PMC7182206

[evaf213-B64] Mizoguchi T, Verkade H, Heath JK, Kuroiwa A, Kikuchi Y. Sdf1/cxcr4 signaling controls the dorsal migration of endodermal cells during zebrafish gastrulation. Development. 2008:135:2521–2529. 10.1242/dev.020107. 18579679

[evaf213-B65] Montague TG, Gagnon JA, Schier AF. Conserved regulation of Nodal-mediated left-right patterning in zebrafish and mouse. Development. 2018:145:dev171090. 10.1242/dev.171090. 30446628 PMC6307886

[evaf213-B66] Mootha VK, et al PGC-1α-responsive genes involved in oxidative phosphorylation are coordinately downregulated in human diabetes. Nat Genet. 2003:34:267–273. 10.1038/ng1180. 12808457

[evaf213-B67] Morel B, Kozlov AM, Stamatakis A, Szollosi GJ. GeneRax: a tool for Species-tree-aware Maximum likelihood-based gene family tree inference under gene duplication, transfer, and loss. Mol Biol Evol. 2020:37:2763–2774. 10.1093/molbev/msaa141. 32502238 PMC8312565

[evaf213-B68] Mukherjee S, et al Sox17 and β-catenin co-occupy Wnt-responsive enhancers to govern the endoderm gene regulatory network. eLife. 2020:9:e58029–e58029. 10.7554/eLife.58029 32894225 PMC7498262

[evaf213-B69] Mukherjee S, Luedeke DM, McCoy L, Iwafuchi M, Zorn AM. SOX transcription factors direct TCF-independent WNT/β-catenin responsive transcription to govern cell fate in human pluripotent stem cells. Cell Rep. 2022:40:111247. 10.1016/j.celrep.2022.111247. 36001974 PMC10123531

[evaf213-B70] Nelson AC, et al Global identification of smad2 and eomesodermin targets in zebrafish identifies a conserved transcriptional network in mesendoderm and a novel role for eomesodermin in repression of ectodermal gene expression. BMC Biol. 2014:12:81. 10.1186/s12915-014-0081-5. 25277163 PMC4206766

[evaf213-B71] Ng CKL, et al Deciphering the Sox-Oct partner code by quantitative cooperativity measurements. Nucleic Acids Res. 2012:40:4933–4941. 10.1093/nar/gks153. 22344693 PMC3367189

[evaf213-B72] Niakan JHMR, et al Sox17 promotes differentiation in mouse embryonic stem cells by directly regulating extraembryonic gene expression and indirectly antagonizing self-renewal. Genes Dev. 2010:24:312–326. 10.1101/gad.1833510. 20123909 PMC2811832

[evaf213-B73] Odenthal J, Nüsslein-Volhard C. Fork head domain genes in zebrafish. Dev Genes Evol. 1998:208:245–258. 10.1007/s004270050179. 9683740

[evaf213-B74] Palasingam P, Jauch R, Ng CKL, Kolatkar PR. The structure of Sox17 bound to DNA reveals a conserved bending topology but selective protein interaction platforms. J Mol Biol. 2009:388:619–630. 10.1016/j.jmb.2009.03.055. 19328208

[evaf213-B75] Pani L, et al Hepatocyte nuclear factor 3β contains two transcriptional activation domains, one of which is novel and conserved with the Drosophila fork head protein. Mol Cell Biol. 1992:12:3723–3732. 10.1128/mcb.12.9.3723-3732.1992. 1324404 PMC360231

[evaf213-B76] Penel S, Menet H, Tricou T, Daubin V, Tannier E. Thirdkind: displaying phylogenetic encounters beyond 2-level reconciliation. Bioinformatics. 2022:38:2350–2352. 10.1093/bioinformatics/btac062. 35139153

[evaf213-B77] Pevny LH, Lovell-Badge R. Sox genes find their feet. Curr Opin Genet Dev. 1997:7:338–344. 10.1016/S0959-437X(97)80147-5. 9229109

[evaf213-B78] Poulain M, Fürthauer M, Thisse B, Thisse C, Lepage T. Zebrafish endoderm formation is regulated by combinatorial Nodal, FGF and BMP signalling. Development. 2006:133:2189–2200. 10.1242/dev.02387. 16672336

[evaf213-B79] Rebagliati MR, Toyama R, Fricke C, Haffter P, Dawid IB. Zebrafish nodal-related genes are implicated in axial patterning and establishing left–right asymmetry. Dev Biol. 1998:199:261–272. 10.1006/dbio.1998.8935. 9698446

[evaf213-B80] Reim G, Mizoguchi T, Stainier DY, Kikuchi Y, Brand M. The POU domain protein spg (pou2/oct4) is essential for endoderm formation in cooperation with the HMG domain protein casanova. Dev Cell. 2004:6:91–101. 10.1016/S1534-5807(03)00396-4. 14723850

[evaf213-B81] Robinson-Rechavi M, Huchon D. RRTree: relative-rate tests between groups of sequences on a phylogenetic tree. Bioinformatics. 2000:16:296–297. 10.1093/bioinformatics/16.3.296. 10869026

[evaf213-B82] Saba R, et al Endocardium differentiation through Sox17 expression in endocardium precursor cells regulates heart development in mice. Sci Rep. 2019:9:11953. 10.1038/s41598-019-48321-y. 31420575 PMC6697751

[evaf213-B83] Sanchez R, et al Phylemon 2.0: a suite of web-tools for molecular evolution, phylogenetics, phylogenomics and hypotheses testing. Nucleic Acids Res. 2011:39:W470–W474. 10.1093/nar/gkr408. 21646336 PMC3125789

[evaf213-B84] Sarkar A, Hochedlinger K. The sox family of transcription factors: versatile regulators of stem and progenitor cell fate. Cell Stem Cell. 2013:12:15–30. 10.1016/j.stem.2012.12.007. 23290134 PMC3608206

[evaf213-B85] Saund RS, et al Gut endoderm is involved in the transfer of left-right asymmetry from the node to the lateral plate mesoderm in the mouse embryo. Development. 2012:139:2426–2435. 10.1242/dev.079921. 22627279 PMC3367449

[evaf213-B86] Schneider I, Houston DW, Rebagliati MR, Slusarski DC. Calcium fluxes in dorsal forerunner cells antagonize β-catenin and alter left-right patterning. Development. 2008:135:75–84. 10.1242/dev.004713. 18045845

[evaf213-B87] Séguin CA, Draper JS, Nagy A, Rossant J. Establishment of endoderm progenitors by SOX transcription factor expression in human embryonic stem cells. Cell stem cell. 2008:3:182–195. 10.1016/j.stem.2008.06.018. 18682240

[evaf213-B88] Sievers F, et al Fast, scalable generation of high-quality protein multiple sequence alignments using Clustal Omega. Mol Syst Biol. 2011:7:539. 10.1038/msb.2011.75. 21988835 PMC3261699

[evaf213-B89] Sievers F, Higgins DG. Clustal Omega for making accurate alignments of many protein sequences. Protein Sci. 2018:27:135–145. 10.1002/pro.3290. 28884485 PMC5734385

[evaf213-B90] Sinclair AH, et al A gene from the human sex-determining region encodes a protein with homology to a conserved DNA-binding motif. Nature. 1990:346:240–244. 10.1038/346240a0. 1695712

[evaf213-B91] Sinner D, Rankin S, Lee M, Zorn AM. Sox17 and beta-catenin cooperate to regulate the transcription of endodermal genes. Development. 2004:131:3069–3080. 10.1242/dev.01176. 15163629

[evaf213-B92] Stafford D, et al Retinoids signal directly to zebrafish endoderm to specify insulin-expressing β-cells. Development. 2006:133:949–956. 10.1242/dev.02263. 16452093

[evaf213-B93] Stefanovic S, et al Interplay of Oct4 with Sox2 and Sox17: a molecular switch from stem cell pluripotency to specifying a cardiac fate. J Cell Biol. 2009:186:665–673. 10.1083/jcb.200901040. 19736317 PMC2742180

[evaf213-B94] Stout CC, Tan M, Lemmon AR, Lemmon EM, Armbruster JW. Resolving Cypriniformes relationships using an anchored enrichment approach. BMC Evol Biol. 2016:16:244. 10.1186/s12862-016-0819-5. 27829363 PMC5103605

[evaf213-B95] Subramanian A, et al Gene set enrichment analysis: a knowledge-based approach for interpreting genome-wide expression profiles. Proc Natl Acad Sci. 2005:102:15545–15550. 10.1073/pnas.0506580102. 16199517 PMC1239896

[evaf213-B96] Sutherland MJ, Ware SM. Disorders of left–right asymmetry: heterotaxy and situs inversus. Am J Med Genet C Semin Med Genet. 2009:151C:307–317. 10.1002/ajmg.c.30228. 19876930

[evaf213-B97] Sutherland RJ . The dorsal diencephalic conduction system: a review of the anatomy and functions of the habenular complex. Neurosci Biobehav Rev. 1982:6:1–13. 10.1016/0149-7634(82)90003-3. 7041014

[evaf213-B98] Talbot CD, et al Eomes function is conserved between zebrafish and mouse and controls left-right organiser progenitor gene expression via interlocking feedforward loops. Front Cell Dev Biol. 2022:10:982477. 10.3389/fcell.2022.982477. 36133924 PMC9483813

[evaf213-B99] Tasnim M, Wahlquist P, Hill JT. Zebrafish: unraveling genetic complexity through duplicated genes. Dev Genes Evol. 2024:234:99–116. 10.1007/s00427-024-00720-6. 39079985 PMC11612004

[evaf213-B100] The UniProt C . UniProt: the universal protein knowledgebase in 2023. Nucleic Acids Res. 2023:51:D523–D531. 10.1093/nar/gkac1052. 36408920 PMC9825514

[evaf213-B101] Thisse B, et al Expression of the zebrafish genome during embryogenesis (NIH R01 RR15402). ZFIN Direct data submission; 2001. https://zfin.org.

[evaf213-B102] Thisse C, Thisse B. High-resolution in situ hybridization to whole-mount zebrafish embryos. Nat Protoc. 2008:3:59–69. 10.1038/nprot.2007.514. 18193022

[evaf213-B103] Traver D, et al Transplantation and in vivo imaging of multilineage engraftment in zebrafish bloodless mutants. Nat Immunol. 2003:4:1238–1246. 10.1038/ni1007. 14608381

[evaf213-B104] Varadi M, et al AlphaFold protein structure database: massively expanding the structural coverage of protein-sequence space with high-accuracy models. Nucleic Acids Res. 2022:50:D439–D444. 10.1093/nar/gkab1061. 34791371 PMC8728224

[evaf213-B105] Viotti M, Niu L, Shi S-H, Hadjantonakis A-K. Role of the gut endoderm in relaying left-right patterning in mice. PLoS Biol. 2012:10:e1001276–e1001276. 10.1371/journal.pbio.1001276. 22412348 PMC3295824

[evaf213-B106] Voldoire E, Brunet F, Naville M, Volff JN, Galiana D. Expansion by whole genome duplication and evolution of the sox gene family in teleost fish. PLoS One. 2017:12:1–20. 10.1371/journal.pone.0180936. PMC5524304 28738066

[evaf213-B107] Wagner DE, et al Single-cell mapping of gene expression landscapes and lineage in the zebrafish embryo. Science. 2018:360:981.LP–981987. 10.1126/science.aar4362. 29700229 PMC6083445

[evaf213-B108] Warga RM, Kane DA. Wilson cell origin for Kupffer’s vesicle in the zebrafish. Dev Dyn. 2018:247:1057–1069. 10.1002/dvdy.24657. 30016568

[evaf213-B109] Wegner M . All purpose sox: the many roles of sox proteins in gene expression. Int J Biochem Cell Biol. 2010:42:381–390. 10.1016/j.biocel.2009.07.006. 19631281

[evaf213-B110] Westerfield M . The zebrafish book. A guide for the laboratory use of zebrafish (Danio rerio). 4th ed. Univ. of Oregon Press, Eugene; 2000.

[evaf213-B111] Wu RS, et al A rapid method for directed gene knockout for screening in G0 zebrafish. Dev Cell. 2018:46:112–125.e114. 10.1016/j.devcel.2018.06.003. 29974860

[evaf213-B112] Yelon D, Horne SA, Stainier DYR. Restricted expression of cardiac myosin genes reveals regulated aspects of heart tube assembly in zebrafish. Dev Biol. 1999:214:23–37. 10.1006/dbio.1999.9406. 10491254

[evaf213-B113] Yuan H, Corbi N, Basilico C, Dailey L. Developmental-specific activity of the FGF-4 enhancer requires the synergistic action of Sox2 and Oct-3. Genes and Development. 1995:9:2635–2645. 10.1101/gad.9.21.2635. 7590241

[evaf213-B114] Zhao J, Lambert G, Meijer AH, Rosa FM. The transcription factor Vox represses endoderm development by interacting with Casanova and Pou2. Development. 2013:140:1090–1099. 10.1242/dev.082008. 23364327

[evaf213-B115] Zhong TP, Childs S, Leu JP, Fishman MC. Gridlock signalling pathway fashions the first embryonic artery. Nature. 2001:414:216–220. 10.1038/35102599. 11700560

