# Supplementary material for: Molecular and Functional Divergence of Zebrafish Sox Paralogs Controlling Endoderm Formation and Left–Right Patterning
Source: Genome Biol Evol. 2025 Nov 11;17(11):evaf213. doi: 10.1093/gbe/evaf213 (PMC12648240; doi:10.1093/gbe/evaf213)
Supplement: evaf213_Supplementary_Data [file evaf213_supplementary_data.zip › Supplementary Figures and Tables_GBE_accepted_version.pdf]

Supplementary Material for:

Molecular and functional divergence of zebrafish Sox paralogues controlling endoderm formation and left-right patterning

Simaran Johal[1,2], Randa Elsayed[3], Dongfeng Wang[4,5], Conor D. Talbot[1], Roberto Feuda[4,5], Kristen A. Panfilio[1,6,7], Andrew C. Nelson[1*]

1. School of Life Sciences, Gibbet Hill Campus, University of Warwick, Coventry, CV4 7AL, UK

2. Current address: Translational and Clinical Research Institute, Herschel Building, Newcastle University, Newcastle Upon Tyne, NE1 7RU, UK

3. Warwick Medical School, Gibbet Hill Campus, University of Warwick, Coventry, CV4 7AL, UK

4. Neurogenetics Group, University of Leicester, Leicester, UK

5. Department of Genetics, Genomics and Cancer Sciences, University of Leicester, Leicester, UK

6. Department of Molecular Genetics, Institute of Biology, University of Hohenheim, Garbenstr. 30, 70599 Stuttgart, Germany

7. Institute for Zoology: Developmental Biology, University of Cologne, Zülpicher Straße 47b, 50674 Cologne, Germany

*Corresponding author
Email: A.Nelson.1@warwick.ac.uk (ACN)

Supplementary Figures

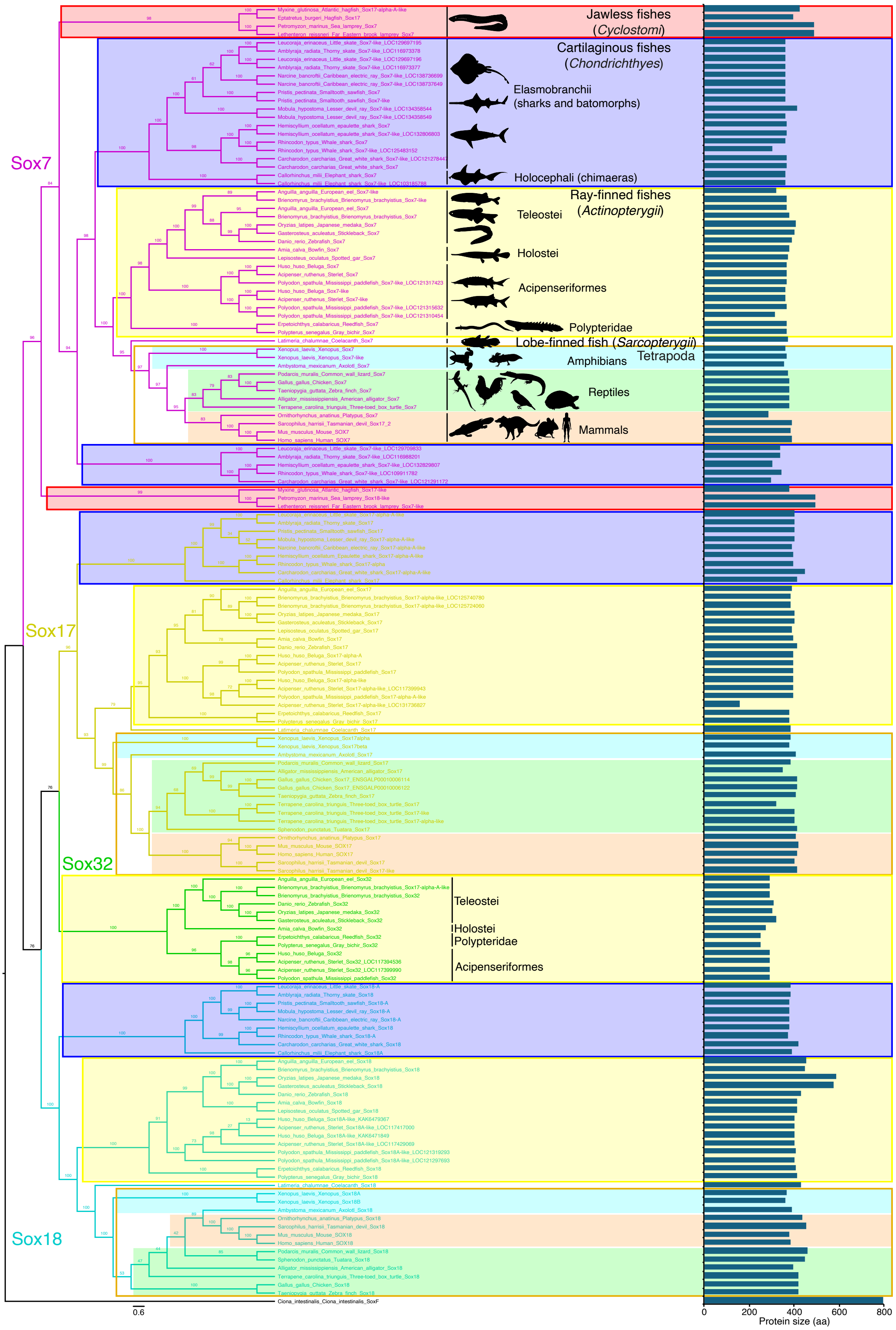

Supplementary Figure 1: Phylogenetic analysis of the origin and evolution of Sox32 within the SoxF subfamily.

Phylogenetic tree of full-length SoxF proteins supports the paralogy of Sox17 and Sox32, with greater sequence divergence in Sox32 proteins. Tree constructed with all SoxF family members identifiable in GenBank and Ensembl from 30 species with representatives from all major branches of the vertebrate evolutionary tree, as indicated. Branches are colour coded according to the individual SoxF transcription factors that they represent. Note that the present names of transcription factors in come species is inconsistent with the branch of the gene tree in why they reside, likely indicating misannotation in current databases. Branches are in colour-coded boxes indicative of the (super)classes of species they contain, with relevant subclassifications also indicated.

The orthologues of Sox7 (purple text and branches) form a well-supported clade separate from the Sox17/18/32, while Sox18 (turquoise text and branches) forms a well-supported clade separate from the Sox17/32. Sox17 (gold text and branches) and Sox32 (green text and branches) occupy separate sub-branches of the tree, with Sox32 only present in ray-finned fish (*Actinopterygii*, yellow box). Overall, orthologue relationships for each Sox factor are consistent with the accepted species tree (see Supplementary Figure 2).

Since Sox17 and Sox32 occupy distinct sub-branches but Sox32 is only present in the *Actinopterygii*, it is not possible to judge from the present tree whether Sox32 arose from a duplication of Sox17 at the root of the *Actinopterygii* followed by rapid divergence, or if Sox32 had an earlier origin and was subsequently lost in *Chondrichthyes* and *Sarcopterygii*.

Protein sizes (in amino acids, aa) for all proteins are indicated in a bar graph on the right. On average, Sox32 proteins are 30% shorter than Sox17 proteins. Thus, branch lengths in the gene tree can be attributed to higher rates of evolutionary divergence of Sox32 proteins, rather than to longer proteins with more alignable positions in the sequence alignment. (In contrast, the single *Ciona* SoxF protein as an outgroup is both large and highly divergent at the amino acid level.)

Interestingly, only Sox17, but not Sox32, could be recovered by BLAST from spotted gar (shown here) or for other gar species present in GenBank, using a range of query sequences and BLAST algorithms. This suggests that there may have been a secondary loss of Sox32 in the *Lepisosteidae*. Fish lineage relationships are based on (Near and Thacker 2024).

Protein sequences were obtained from Ensembl (accessions beginning with “EN”) or Genbank and are listed in Supplementary Table 1.

Cartoon images of representative species were obtained from Phylopic.org. We thank C. Camilo Julián-Caballero, Tony Ayling (vectorized by Milton Tan), Erika Schumacher, Ian Quigley, Edwin Price and Sarah Werning for providing images under Creative Commons licences CC BY 3.0, CC BY-SA 3.0, CC BY-NC 3.0 or CC BY 4.0 as indicated at the prior links.

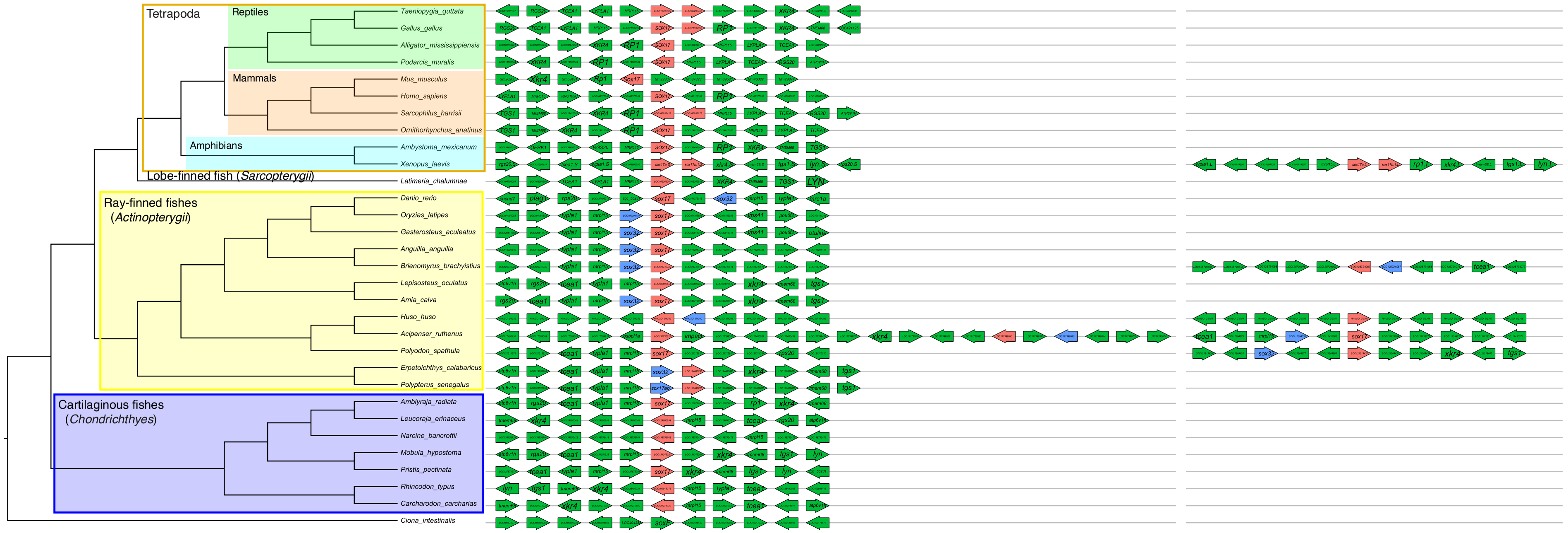

Supplementary Figure 2: Synteny analysis of the *sox17* gene neighbourhood indicates *sox32* proximal and typically adjacent to *sox17* throughout the *Actinopterygii*. Species tree constructed according to evolutionary relationships defined by (Amaral, et al. 2018; Near and Thacker 2024), representing species in Supplementary Figure 1. Also shown are the *Sox17* gene neighborhoods in these species, extracted using OrthoFinder (Emms and Kelly 2019). All identified *sox17* orthologues (peach), and *sox32* orthologues (blue) where present, plus the 5 flanking genes (green) as shown. Where multiple *sox17 genes* are present within a species, multiple gene clusters are shown. Where OrthoFinder failed to discover *sox17/32* orthologues previously identified in NCBI or Ensembl databases, these have been manually inserted. This includes for *Erpetoichthys calabaricus sox32* and *Polypterus senegalus sox17ab* (*sox32* orthologue) which are annotated in Ensembl but not RefSeq.

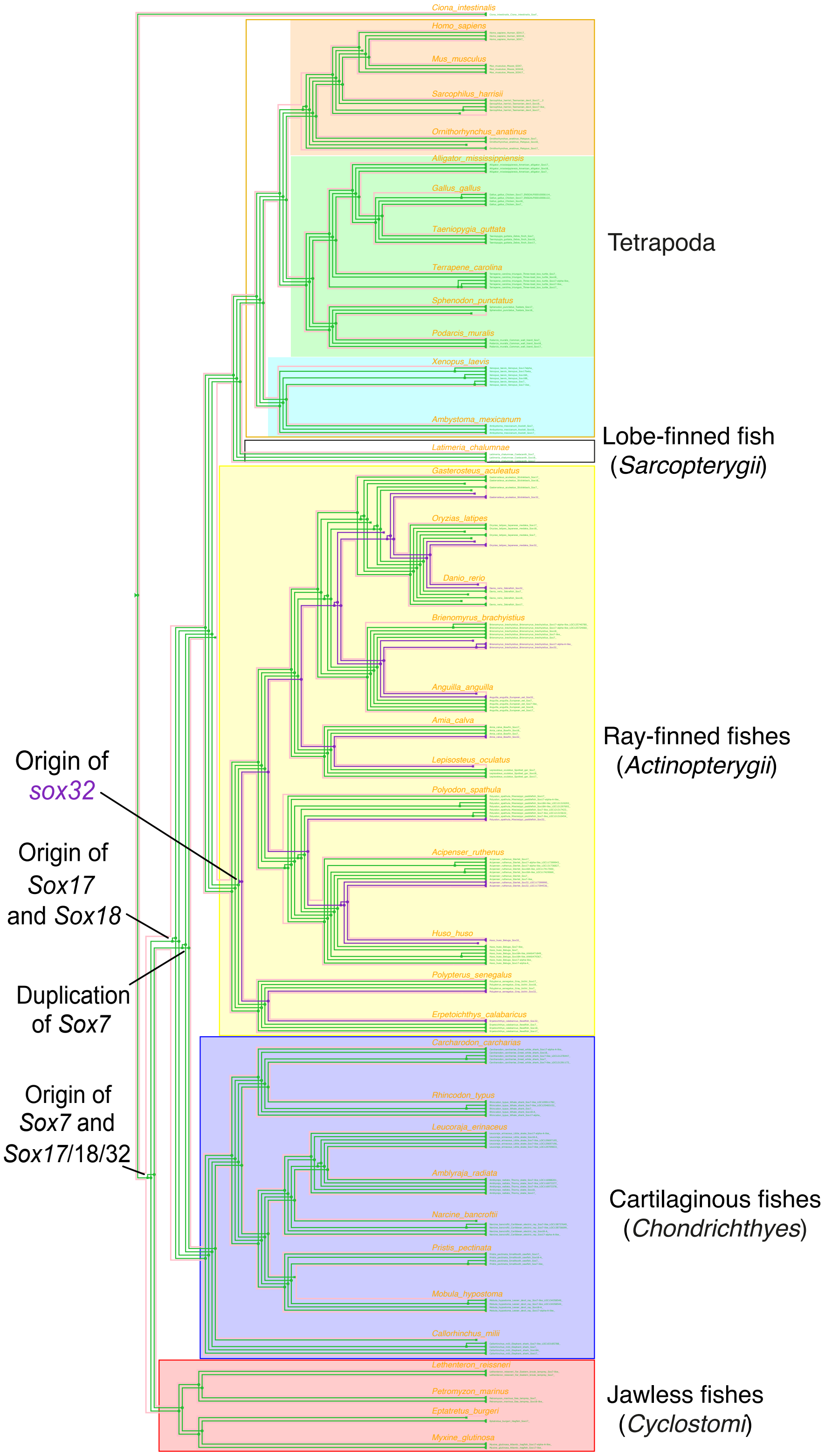

Supplementary Figure 3: Reconciliation of gene and species trees indicate *sox32* likely originated from duplication of *sox17* at the root of the *Actinopterygii.* The gene tree (Supplementary Figure 1) was reconciled with the species tree using GeneRax (Morel, et al. 2020). Nodes where individual *SoxF* genes are predicted to have been duplicated and the resulting gene identities are annotated. *Sox32* genes are indicated in purple and all other *SoxF* subfamily genes in green. Branches are in colour-coded boxes indicative of the (super)classes of species they contain, as in Supplementary Figure 1.

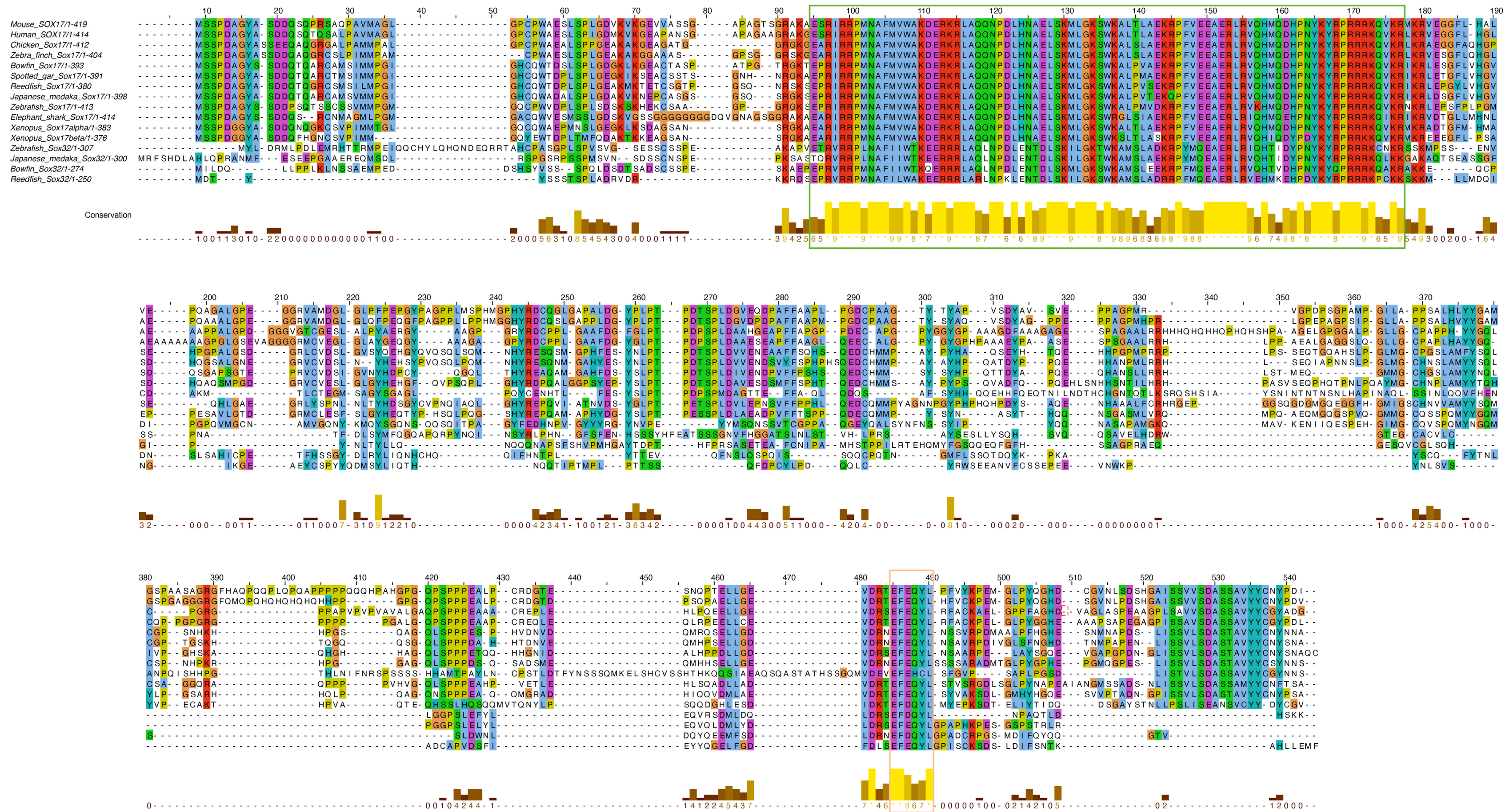

Supplementary Figure 4: Alignments of tetrapod Sox17, and fish Sox17 and Sox32. Species and order are as in Supplementary Figure S1A. Alignments conducted using MuscleWS in JalView and colour coded based on conserved amino acid properties. Conservation levels calculated according to Analysis of Multiply Aligned Sequences (AMAS) are displayed. Green box - HMG domain; peach box – CTD EF(E/D)QYL helix. Ensembl or GenBank protein sequences are as listed in Supplementary Figure S1 legend.

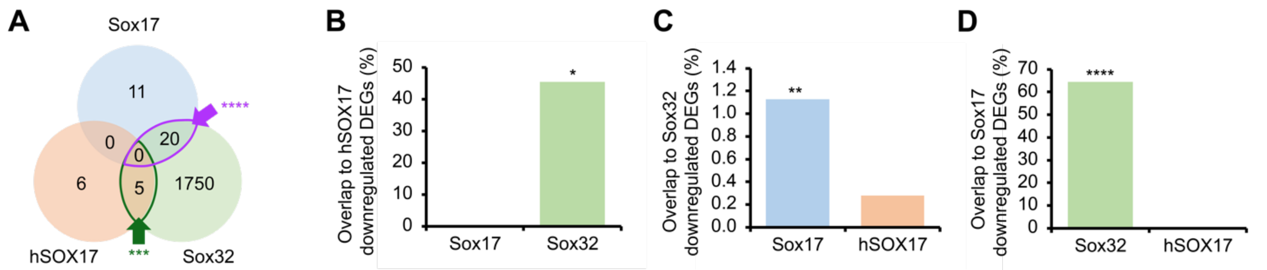

Supplementary Figure 5: Downregulated differentially expressed genes (DEGs) analysis for hSOX17, Sox32 and Sox17 RNA-seq. A) Venn diagram indicating overlap in genes downregulated by overexpression of each of the TFs. Bar charts indicating percentage overlap of significantly downregulated genes on hSOX17 OE (B), Sox32 OE (C), and Sox17 OE (D) to those downregulated by the other TFs. Bars represent the percentage of genes downregulated by the factor on the y-axis also downregulated by the factor on the x-axis. Statistical tests to determine whether the overlap was significantly greater with one factor on the x-axis compared to the other were carried out using Fisher's Exact test. Note that the only significant overlap (as determined using Fisher's Exact test) is between genes downregulated by Sox32 and Sox17. * $P < 0.02$; ** $P < 0.003$; *** $P < 2 \times 10^{-6}$; **** $P < 1 \times 10^{-8}$.

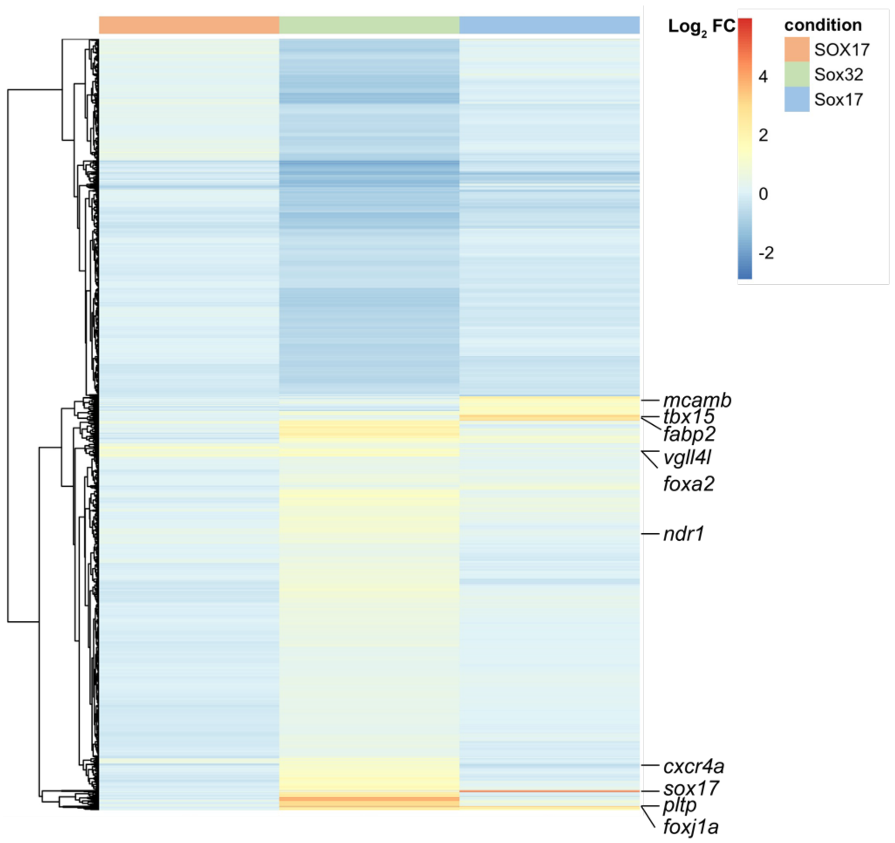

Supplementary Figure 6: hSOX17, Sox32 and Sox17 induce quantitatively different changes in gene expression on overexpression in early zebrafish embryos. RNA-seq heatmap of all 3866 differentially expressed genes (DEGs, Bonferroni adjusted $P < 0.05$, Supplementary Table S1-3), displayed as log2 fold change relative to control.

A

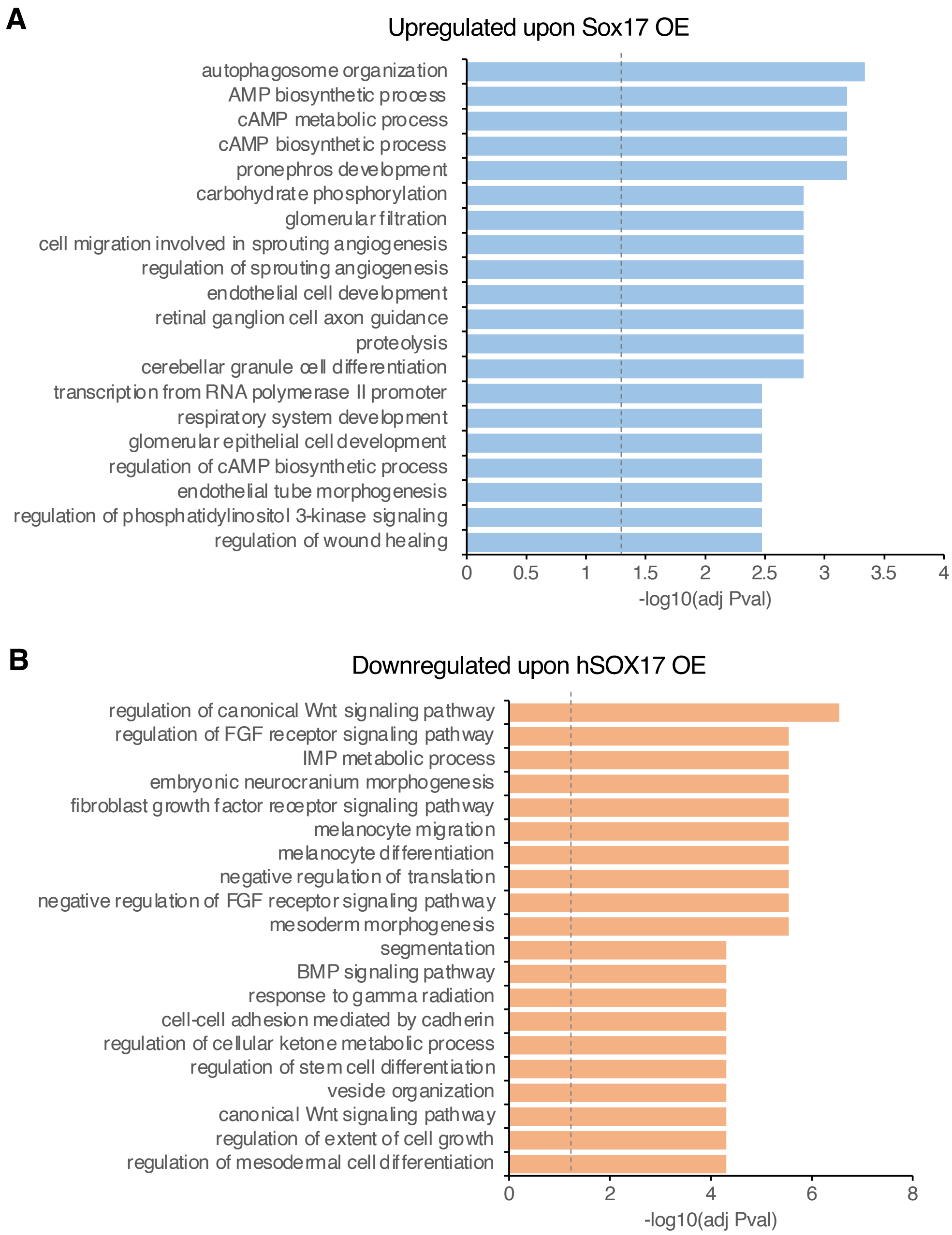

Supplementary Figure 7: Functional annotation analysis carried out using fishEnrichR for Sox17 (A) and hSOX17(B). A) GO analysis carried out on upregulated DEGs due to Sox17 OE. B) GO analysis carried out on downregulated DEGs due to hSOX17 OE. FDR<0.05 cut off applied with top 20 biological process terms graphed. Vertical lines indicate enrichment score from FDR = 0.05

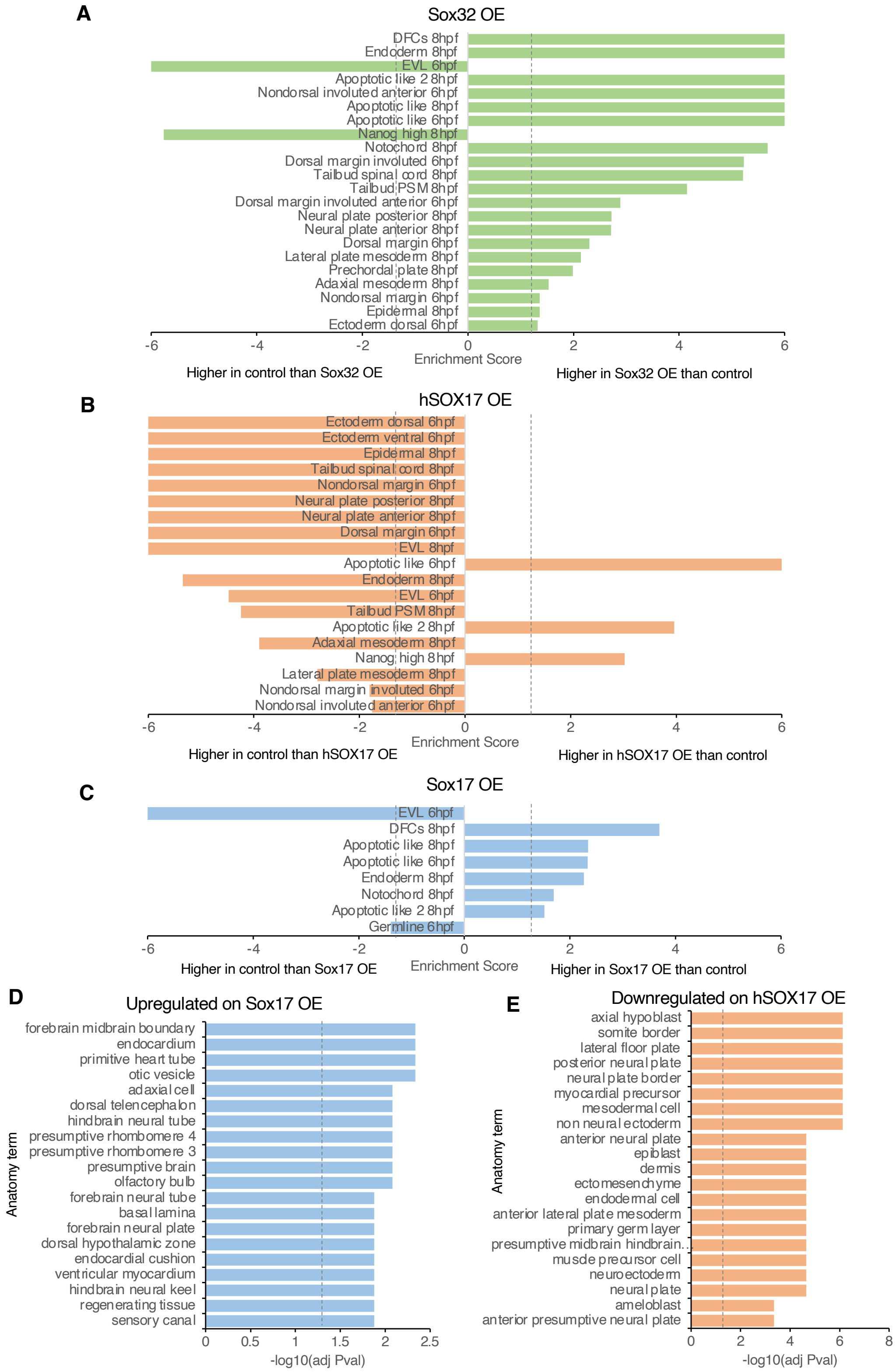

Supplementary Figure 8: Anatomical enrichment analysis. (A-C) Gene set enrichment analysis (GSEA) carried out against cell type markers defined by scRNA-seq at 6-8 hpf (Wagner, et al. 2018; Near and Thacker 2024). Enrichment score was calculated through -$\log_{10}$(FDR q-value) with a cut off of FDR q-value <0.05 applied. Vertical lines indicate enrichment score from FDR = 0.05. EVL = enveloping layer, DFC= dorsal forerunner cell, PSM = presomitic mesoderm, nondorsal involuted anterior includes the presumptive endoderm. (A) Sox32 upregulated transcripts show strongest enrichment for 8 hpf endoderm and DFC markers as expected. Conversely, hSOX17 OE shows negative enrichment for endoderm markers (B). Sox17 overexpression shows induction of 8 hpf endoderm markers but at a much weaker levels compared to Sox32 and lacks enrichment for markers of “non-dorsal involuted anterior” as defined by Wagner et al., which includes the presumptive endoderm. (D-E) Anatomy term enrichment analysis carried out using fishEnrichR for genes significantly upregulated by Sox17 (D) and genes significantly downregulated by hSOX17(E).

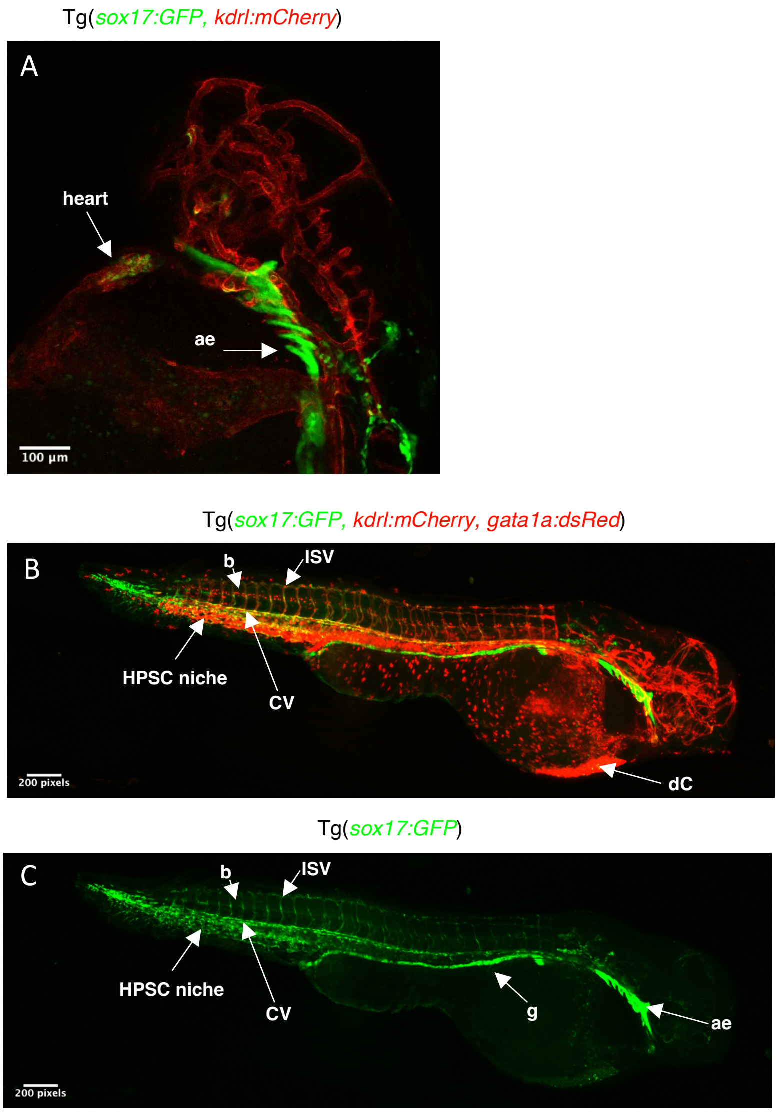

Supplementary Figure 9: Expression of *sox17:GFP* in the heart and vasculature. Lateral images of 48hpf heterozygous *sox17:GFP* and *kdrl:mCherry* embryos **(A)** and triple transgenic embryos heterozygous to *sox17:GFP*, *kdrl:mCherry* and *gata1a:dsRed* **(B-C)**. **(A)** *sox17:GFP* exhibits co-expression with *kdrl:mCherry* in the heart, with *kdrl:mCherry* marking the endocardium specifically. A) Maximum Z projection of all slices. **(B)** *sox17:GFP* exhibits co-localisation with endothelial marker *kdrl:mCherry* in the intersomitic vessels (ISV) and cardinal vein (CV), with a lack of co-expression in the duct of Cuvier (dC). Additional *sox17:GFP* co-expression is observed with erythroid marker *gata1a:dsRed* in the blood (b) and haemopoietic stem and progenitor cell (HPSC) niche.**(C)** Identical image to C but presenting *sox17:GFP* expression alone to show presence within endodermal, endothelial and erythroid lineages. ae = anterior endoderm, HSPC = haemopoietic stem and progenitor cell niche, b = blood, CV = cardinal vein, ISV = intersomitic vessels, dC = duct of Curvier, g = gut.

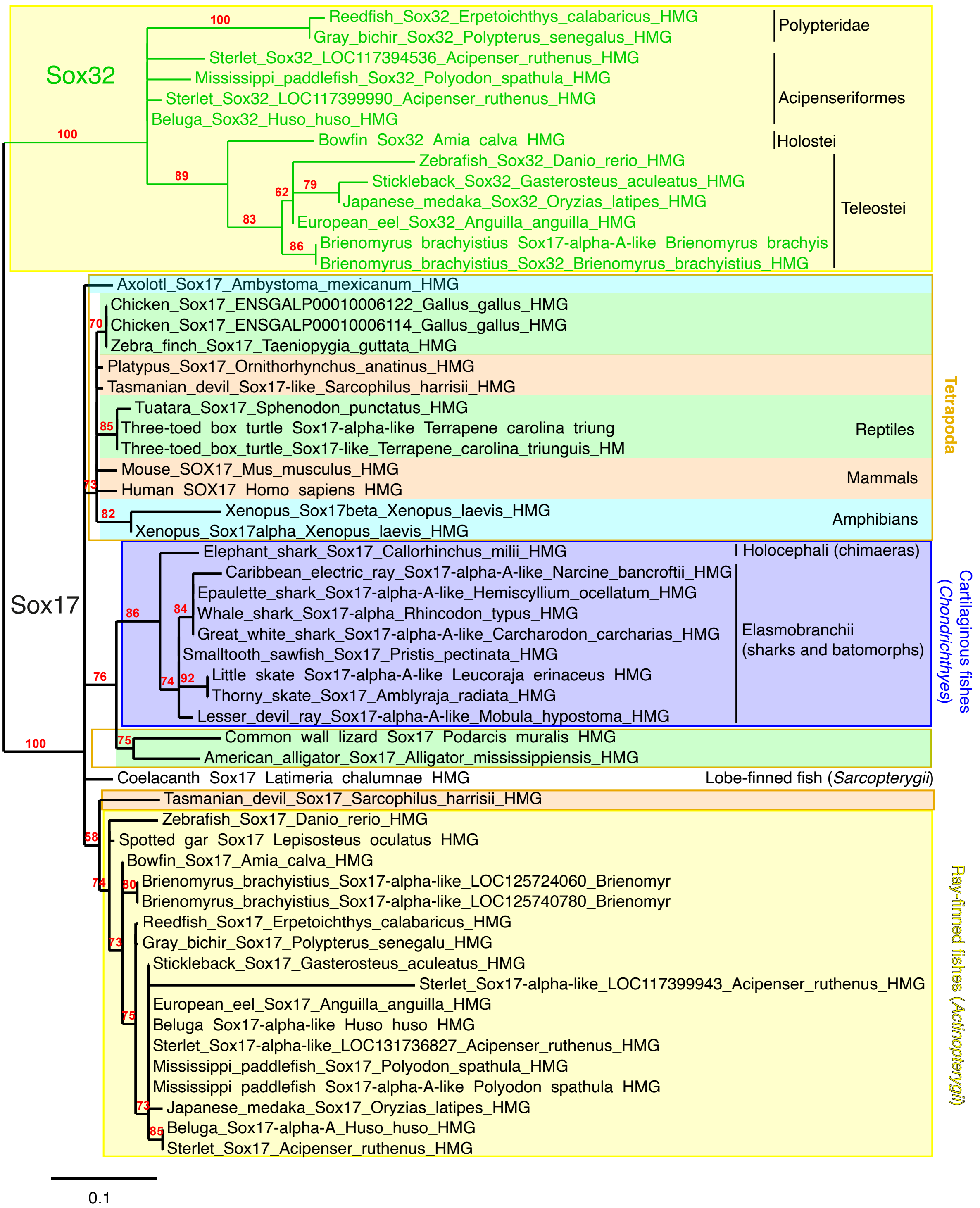

Supplementary Figure 10: Phylogenetic analysis indicates divergence of the Sox32 HMG domain after duplication of the ancestral *sox17* gene. Phylogenetic analysis of HMG domains for Sox17 and Sox32 proteins represented in Supplementary Figure 1. HMG amino acid sequences used are provided in Supplementary Table 1. Branches are in colour-coded boxes indicative of the (super)classes of species they contain, as in Supplementary Figure 1. Sox32 HMG domains form a separate well-supported clade, suggesting rapid evolution after duplication of the ancestral *sox17* gene. Fish lineage relationships are based on (Near and Thacker 2024).

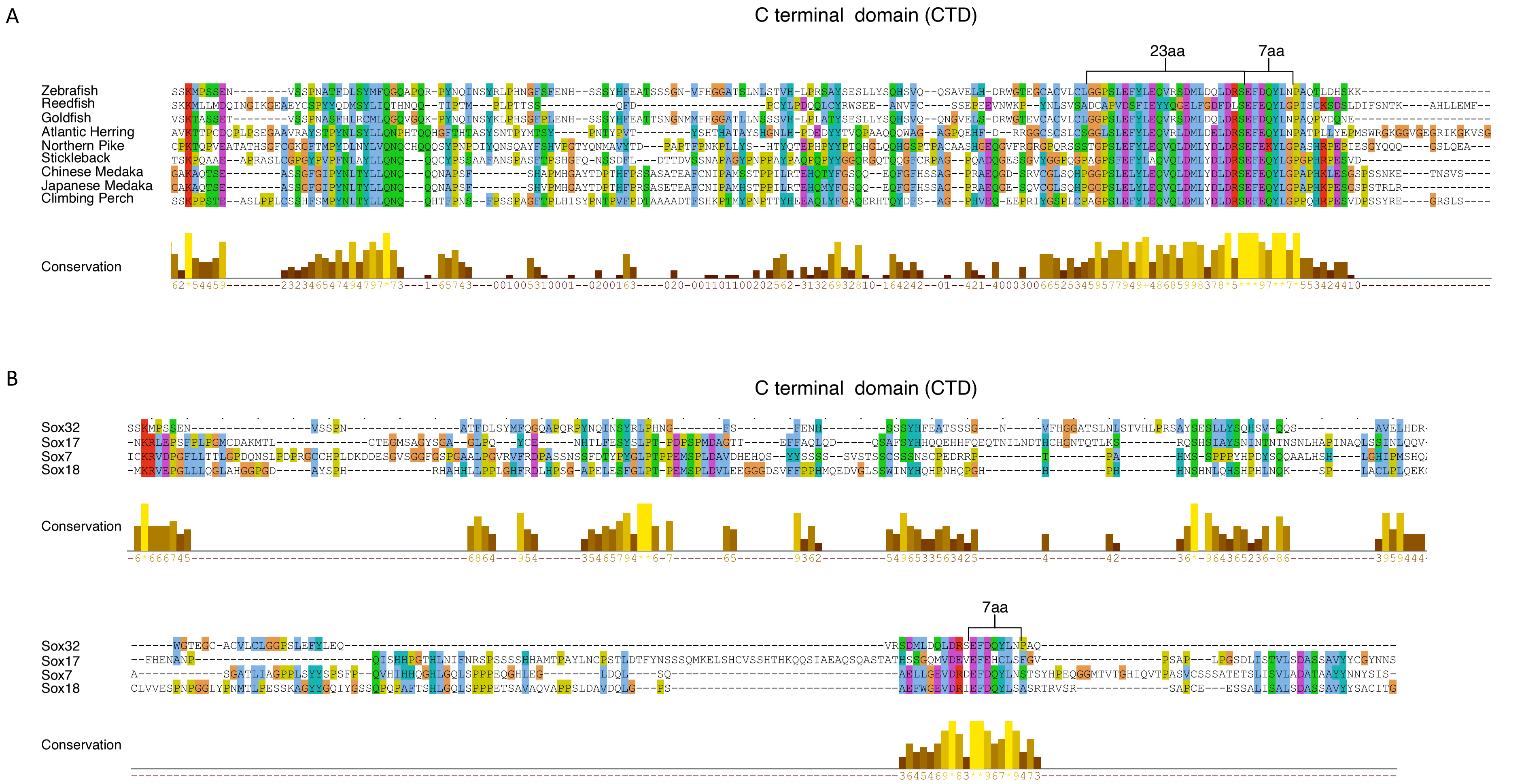

Supplementary Figure 11: Alignments of Sox32 C terminal domain with Sox32 orthologues (A) and zebrafish SoxF subfamily proteins (B). Alignments conducted using ClustalOmega in JalView and colour coded based on conserved amino acid properties. Conservation levels calculated according to Analysis of Multiply Aligned Sequences (AMAS) are displayed. A) Annotation of highly conserved 23 aa, plus the 7 aa peptide within the CTD previously identified by Kikuchi et al. (2001) and Sinner et al., (2004), B) Annotation of 7 aa peptide (equivalent to 7 aa domain in A) within the CTD. Indicates a high degree of conservation between Sox32, Sox7 and Sox18, while Sox17 shows divergence.

Group	Sequence	1	2	3	4	5	6
	Consensus	E GAG	F TTT	E GAG	Q CAG	Y TAC	L CTG
Chondrichtheyes	Elephant shark *sox17*	E GAG	F TTC	E GAG	Q CAA	Y TAC	L CTC
Polypteridae	Reedfish *sox17*	E GAG	F TTT	E GAG	Q CAG	Y TAC	L CTG
	Reedfish *sox32*	E GAA	F TTT	E GAG	Q CAG	Y TAT	L CTT
	gray bichir *sox17*	E GAG	F TTT	E GAG	Q CAG	Y TAC	L CTG
	gray bichir *sox32*	E GAG	F TTT	E GAG	Q CAG	Y TAC	L CTG
Acipenseriformes	Sterlet *sox17*	E GAA	F TTT	E GAG	Q CAG	Y TAC	L CTG
	Sterlet sox17-alpha-B-like (*sox32*)	E GAA	L CTG	E GAG	Q CAG	Y TAT	L CTT
	Mississippi paddlefish *sox17*	E GAA	F TTT	E GAG	Q CAG	Y TAC	L CTG
	Mississippi paddlefish *sox32*	E GAA	L CTG	E GAG	Q CAG	Y TAT	L CTT
Holostei	Spotted gar *sox17*	E GAG	F TTT	E GAA	Q CAG	Y TAT	L TTG
	bowfin *sox17*	E GAA	F TTT	E GAA	Q CAA	Y TAT	L TTG
	bowfin *sox32*	E GAA	F TTT	D GAT	Q CAA	Y TAT	L CTT
Teleostei	Zebrafish *sox17*	E GAG	F TTT	E GAG	H CAC	C TGT	L CTC
	Zebrafish *sox32*	E GAA	F TTT	D GAC	Q CAG	Y TAC	L CTC
	Atlantic cod *sox17*	Peptide lost					
	Atlantic cod *sox32*	E GAG	F TTC	E GAG	Q CAG	Y TAC	L CTG
	Stickleback *sox17*	E GAG	F TTC	E GAG	Q CAG	Y TAT	L TTG
	Stickleback *sox32*	E GAG	F TTT	E GAA	Q CAG	Y TAC	L CTG
	Medaka *sox17*	E GAG	F TTC	E GAG	Q CAG	Y TAT	L CTA
	Medaka *sox32*	E GAG	F TTT	E GAA	Q CAG	Y TAC	L TTG
Mammalia	Human *SOX17*	E GAA	F TTT	E GAA	Q CAG	Y TAT	L CTG
	Mouse *Sox17*	E GAA	F TTC	E GAA	Q CAG	Y TAT	L CTG
Aves	Chicken *Sox17*	E GAG	F TTC	E GAG	Q CAG	Y TAC	L CTG
	Zebra finch *Sox17*	E GAG	F TTC	E GAG	Q CAG	Y TAC	L CTG
Amphibia	Xenopus Sox17alpha	E GAA	F TTC	E GAG	Q CAG	Y TAT	L CTG
	Xenopus Sox17beta	E GAG	F TTT	D GAC	Q CAG	Y TAC	L CTT
		**	*	**	**	*	*

Supplementary Figure 12: Alignment of putative β-catenin interacting peptide in the CTD helices of Sox17 and Sox32 across selected fish and tetrapod species. Codons are presented with the encoded amino acid above. The consensus sequence was calculated using EMBOSS Cons. Base substitutions relative to the consensus sequence are colour-coded. Green = synonymous change in the first position; yellow = synonymous change in the third position; lilac = non-synonymous change; * = base conserved across all species. Ensembl or GenBank accessions for encoding transcripts are: Elephant shark (*Callorhinchus milii sox17*: ENSCMIT00000039060.1); reedfish (*Erpetoichthys calabaricus sox17: ENSGT00940000156694, sox32: ENSECRT00000007161); gray bichir (Polypterus senegalu sox17*: XM_039754518.1, *sox32*: JAATIS010007298.1); Sterlet (*Acipenser ruthenus sox17*: XM_033992297.3, *sox32*: XM_033999454.2); Mississippi paddlefish (*Polyodon spathula sox17*: XM_041247570.1, *sox32*: XM_041241218.1); spotted gar (*Lepisosteus oculatus sox17*: ENSLOCT00000006828); bowfin (*Amia calva sox17*: XM_066719778.1, *sox32*: XM_066689702.1); zebrafish (*Danio rerio sox17*: ENSDART00000167433.3, *sox32*: ENSDART00000171095.3); Atlantic cod (*Gadus morhua sox17*: ENSGMOT00000029202.1, *sox32*: ENSGMOT00000006450.2); stickleback (*Gasterosteus aculeatus aculeatus sox17*: ENSGACT00000005749.2, *sox32*: ENSGACT00000048202.1); medaka (*Oryzias latipes sox17*: ENSORLT00000014462.2, *sox32*: ENSORLT00000014442.2); human (*Homo sapiens SOX17*: ENST00000297316.5); mouse (*Mus musculus Sox17*: ENSMUST00000027035.10); chicken (*Gallus gallus Sox17*: ENSGALT00010010493); zebra finch (*Taeniopygia guttata Sox17*: ENSTGUT00000034496); African clawed frog (*Sox17α*: NM_204094.1, *Sox17β*: NM_203518.1);

Cypriniformes sox17

Catostomidae	*Xyrauchen texanus*	E	F	E	Q	Y	L
	(Razorback sucker)	gag	ttt	gag	caa	tac	ctc
Cobitidae	*Misgurnus anguillicaudatus*	E	F	E	Q	Y	L
	(Pond loach)	gag	ttt	gag	cag	tac	ctc
	Paramisgurnus dabryanus	E	F	E	Q	Y	L
		gag	ttt	gag	cag	tac	ctc
Cyprinidae	*Cyprinus carpio carpio*	E	F	E	Q	C	L
	(Common carp)	gag	ttt	gag	caa	tgc	ctc
	Carassius auratus	E	F	E	Q	C	L
	(Goldfish)	gag	ttt	gag	caa	tgc	ctt
	Carassius gibelio	E	F	E	Q	C	L
	(Prussian carp)	gag	ttt	gag	caa	tgc	ctt
	Labeo rohita	A	F	E	Q	C	L
	(Rohu)	gct	ttt	gag	caa	tgt	ctc
Danionidae	*Danio aesculapii*	E	F	E	H	C	L
	(Panther danio)	gag	ttt	gag	cac	tgt	ctc
	Danio rerio	E	F	E	H	C	L
	(Zebrafish)	gag	ttt	gag	cac	tgt	ctc

Supplementary Figure 13: Alignment of putative β-catenin interacting peptide in the CTD helices of Sox17 across selected *Cypriniformes* fish species. Codons are presented with the encoded amino acid above. Fish species are classified according to the NCBI Taxonomy database. Base substitutions relative to *Xyrauchen texanus* (with which the other species share the most ancient common ancestor) are colour-coded. Yellow = synonymous change in the third position; lilac = non-synonymous change. Ensembl or GenBank accessions for encoding transcripts are: *Xyrauchen texanus* - XM_0521475221; *Misgurnus anguillicaudatus* - XM_0552171151; *Paramisgurnus dabryanus* - XM_0652617881; *Cyprinus carpio carpio* - ENSCCRT000001986421; *Carassius auratus* - ENSCART000001273041; *Carassius gibelio* - XM_0525604591; *Labeo rohita* - XM_0511157551; *Danio aesculapii* - XM_056462958; *Danio rerio* - ENSDART00000167433.

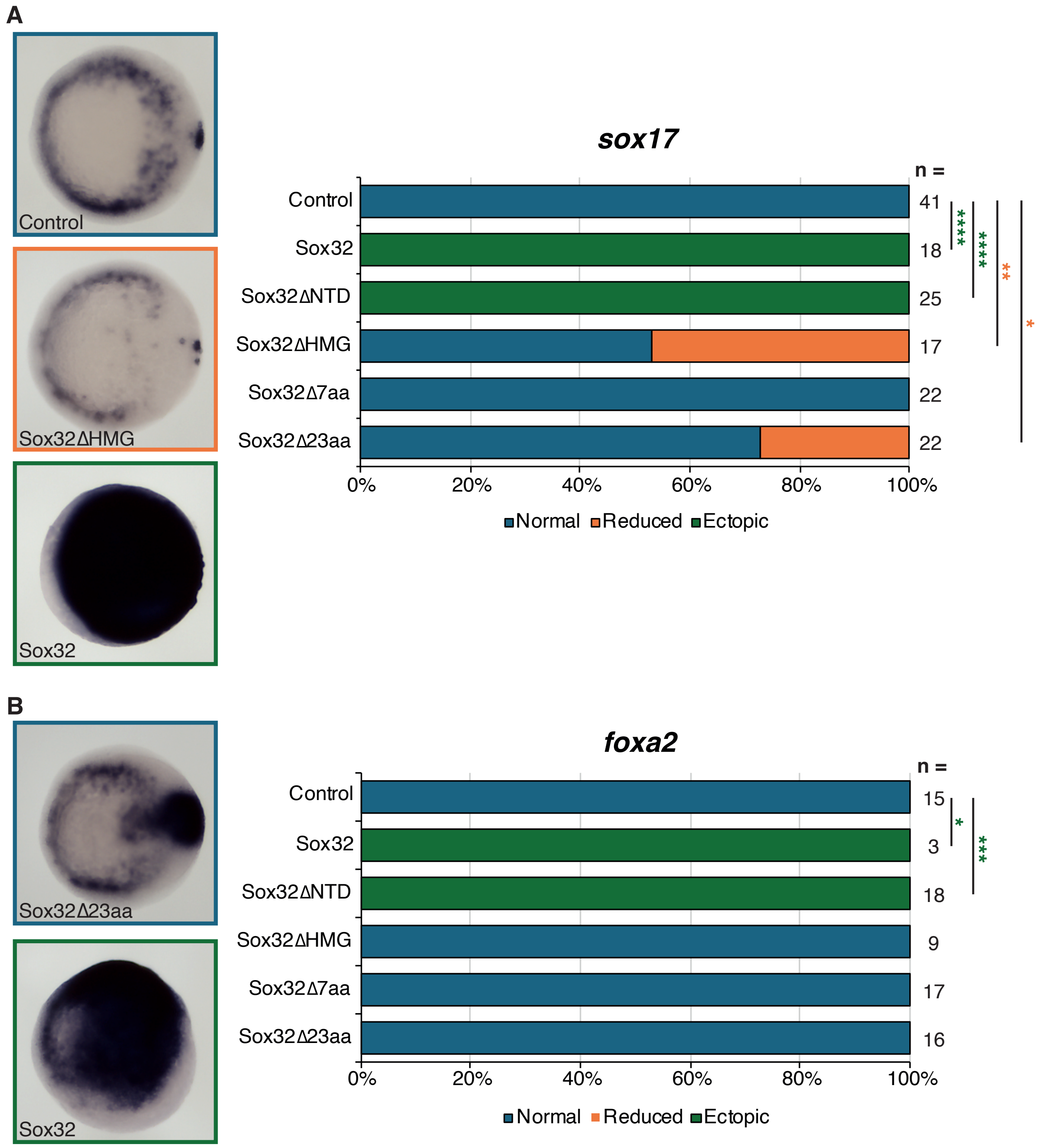

Supplementary Figure 14: *In situ* hybridisation analysis of *sox17* and *foxa2* under the same conditions as Figure 4. Analysis of endoderm and DFC marker *sox17* (A) and endoderm and axial chorda mesoderm marker *foxa2* (B) in early/mid gastrula (6.5 h.p.f.) embryos. Animal pole views; dorsal to the right. Embryos were injected at the 1 cell stage to overexpress full-length Sox32 or different mutant forms as indicated. Total numbers of embryos scored per condition are indicated. Representative images of expression patterns per gene per category are shown. Fisher's Exact two-tailed probability test raw P values: * $P \leq 1 \times 10^{-3}$; ** $P = 1 \times 10^{-5}$; *** $P \leq 1 \times 10^{-9}$; **** $P \leq 1 \times 10^{-4}$. Green asterisks indicate significant differences in fractions of embryos exhibiting ectopic expression vs. other categories. Orange asterisks indicate significant differences in fractions of embryos exhibiting reduced expression vs. other categories.

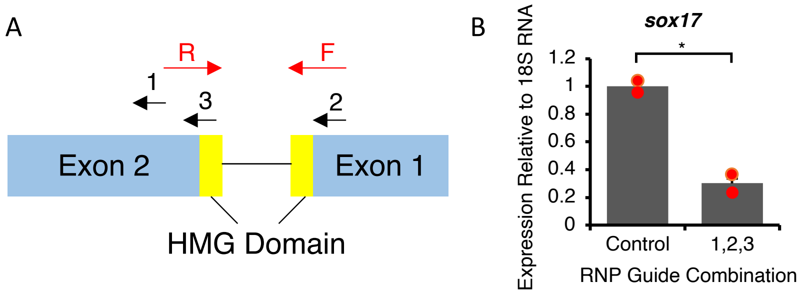

Supplementary Figure 15: Guide RNAs against *sox17* efficiently disrupt the gene locus A) Gene model representing the exons and HMG domain of Sox17 with gRNA positioning (1-3) and qPCR primers (F,R) annotated**. B)** *sox17* qPCR normalised to 18S rRNA on 75% epiboly (8 hpf) control or *sox17* Cas9 RNP injected embryos with indicated gRNAs. Statistically significant differences were inferred using Students t-test test on independent biological duplicate datasets, * P < 0.05. Error bars represent relative standard deviation.

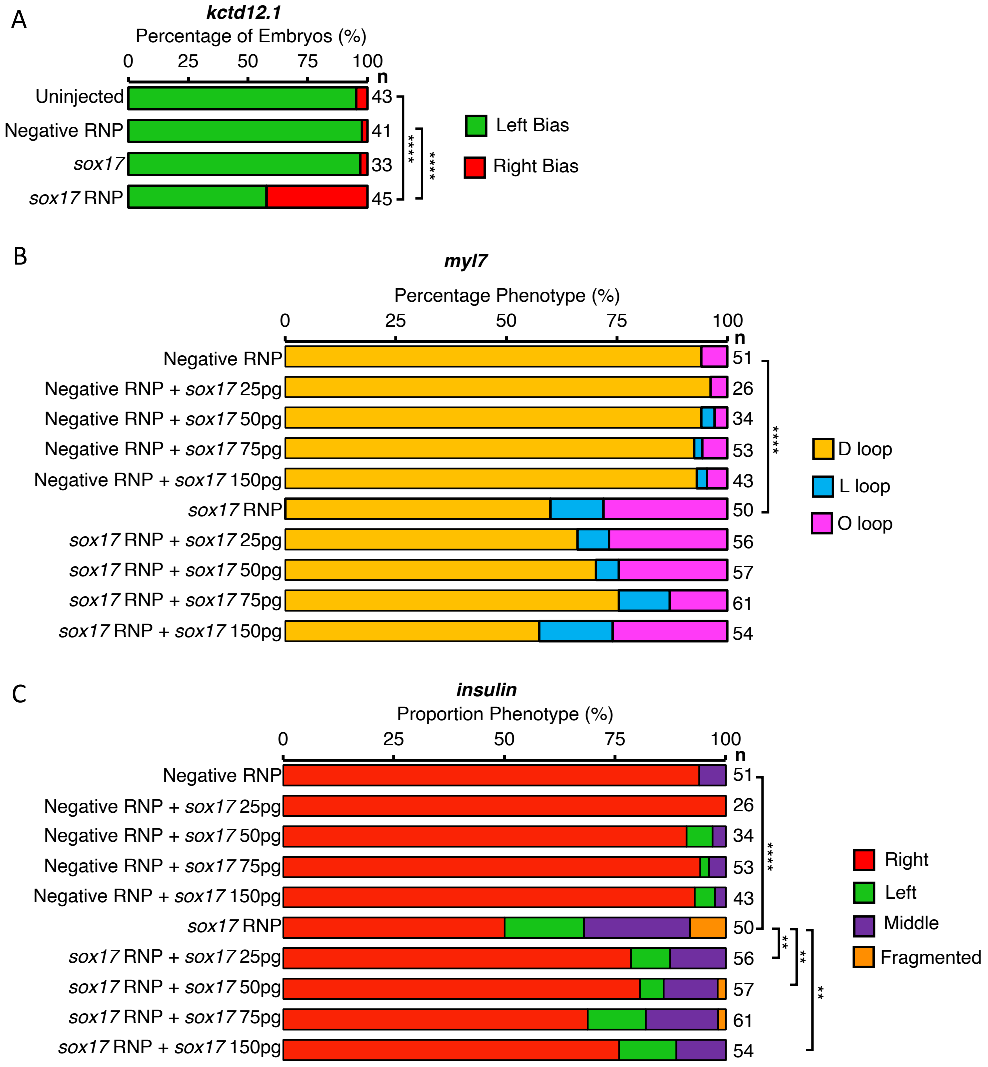

Supplementary Figure 16: Sox17 overexpression does not affect organ asymmetry. A) Whole mount *in situ* hybridisation (WISH) for asymmetrically expressed habenula marker *kctd12.1* carried out at 4 dpf. Statistically significant differences in categorical scoring was inferred using Fisher's Exact test on independent biological triplicate datasets. **B)** WISH for cardiac marker *myl7* at 48 hpf. Experiment carried out on a range of conditions to investigate dose dependent overexpression of Sox17 and rescue of Sox17 RNP phenotype by Sox17 mRNA. **C)** WISH for endocrine pancreas marker *insulin* at 48 hpf. Experiment carried out on a range of conditions to investigate dose dependent overexpression of Sox17 and rescue of Sox17 RNP phenotype by Sox17 mRNA. Statistically significant differences in categorical scoring was inferred using Fisher's Exact test on independent biological duplicate datasets. Raw P values are indicated - ** P<0.01, **** P<0.0001. n = number of embryos analysed. Representative images can be viewed in Figure 6.

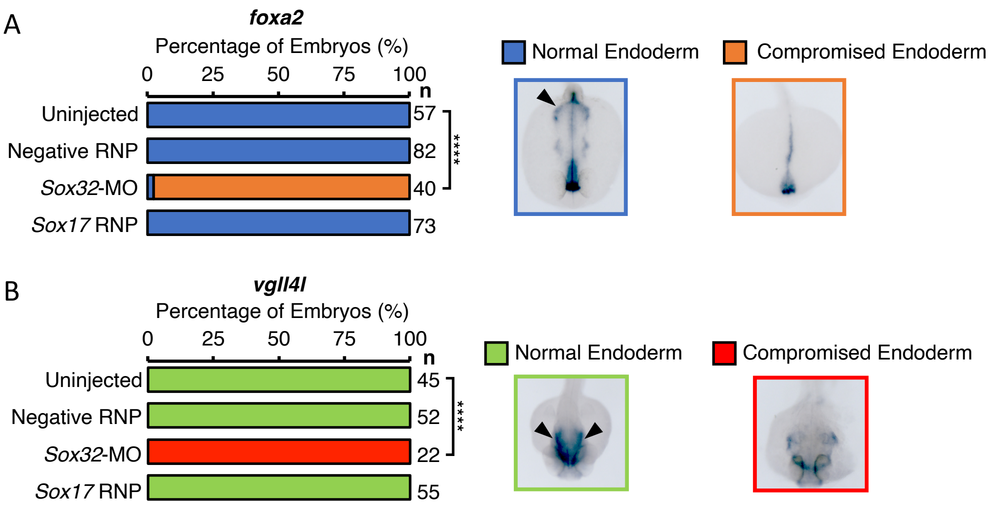

Supplementary Figure 17: Anterior endoderm is present in Sox17 CRISPants. Whole mount *in situ* hybridisation for anterior endoderm in uninjected, Negative RNP, *sox32* MO (positive control) and *sox17* RNP injected embryos. **A)** *foxa2* at 24 hpf. **B)** *vgll4l* at 48 hpf. Dorsal views with anterior to the bottom, arrowheads indicate anterior endoderm. Statistically significant differences in categorical scoring was inferred using Fisher's Exact test on independent biological triplicate datasets, Raw P values are indicated - **** P < 0.0001. Sox32 MO injected embryos showed compromised, absent anterior endoderm as expected, while anterior endoderm appears normal in Sox17 CRISPants. n = total number of embryos analysed.

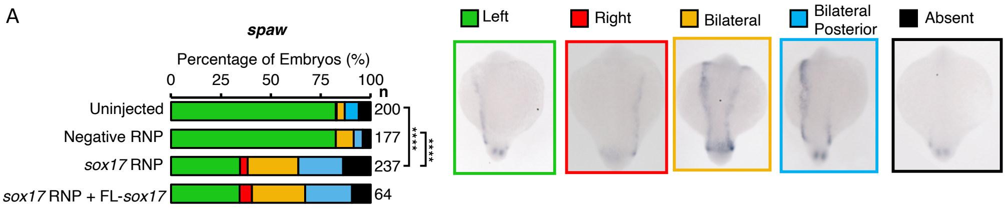

Supplementary Figure 18: Sox17 CRISPants exhibit abnormal *spaw* expression that cannot be rescued through co-injection of exogenous *sox17* mRNA. Wholemount *in situ* hybridisation for *spaw* conducted at 18 somite stage. Sox17 CRISPants show a significant increase in the percentage of embryos depicting abnormal *spaw* expression, phenotypes include right LPM expression, bilateral, bilateral posterior and absent expression. Abnormal *spaw* expression could not be rescued by co-injection of sox17 RNP with exogenous sox17 mRNA. Statistically significant differences in categorical scoring was inferred using Fisher's Exact test on independent biological triplicate datasets, **** P<0.0001 (raw P values). n= number of embryos analysed.

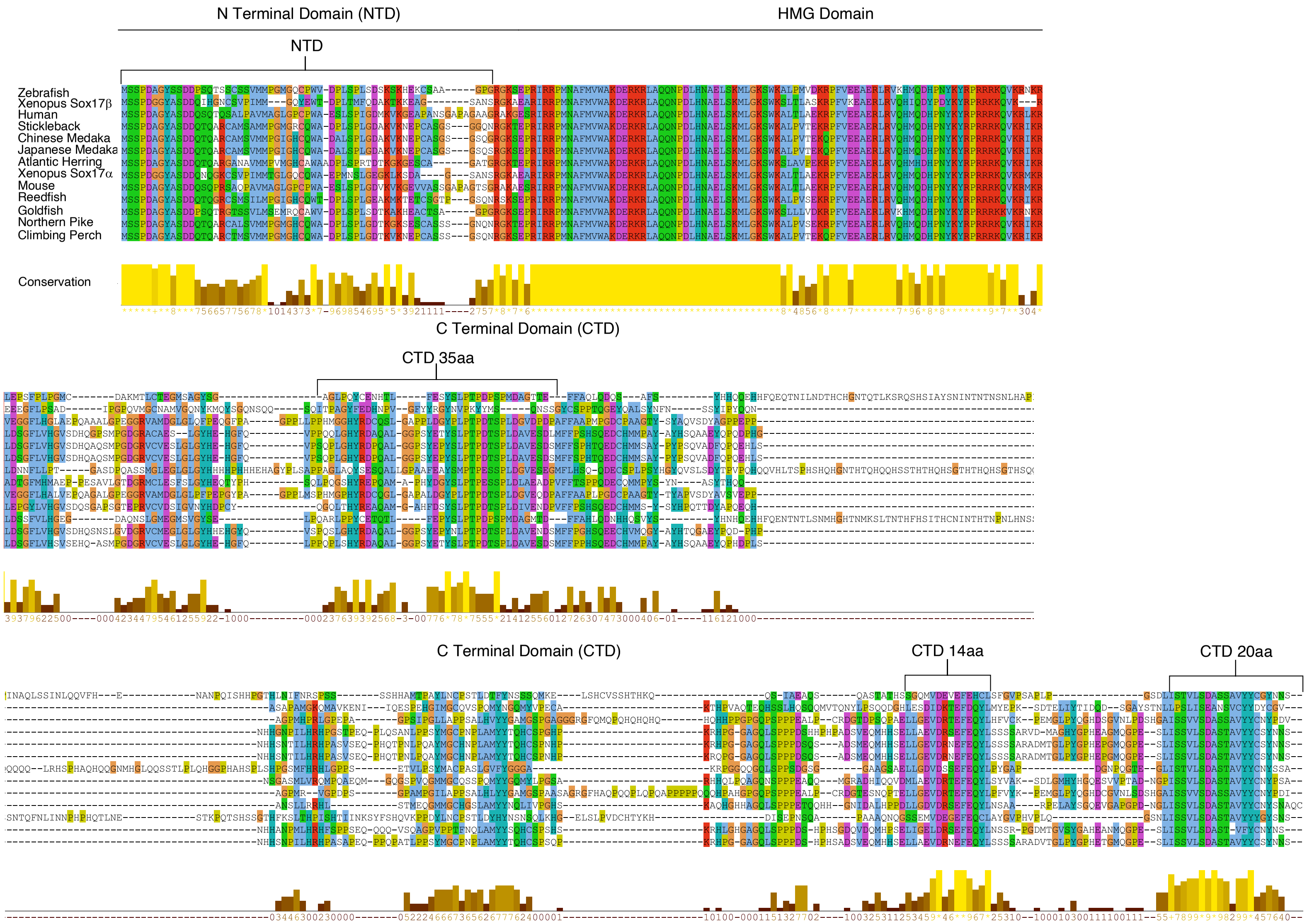

Supplementary Figure 19: Alignment of Sox17 teleost and mammalian orthologues. Alignment carried out using Clustal Omega in Jalview and colour coded based on conserved amino acid properties. Conservation levels calculated according to Analysis of Multiply Aligned Sequences (AMAS) are displayed. Four distinct highly conserved domains predicted to be important for the role of Sox17 in LR patterning selected for deletion are indicated in brackets.

Supplementary Video 1 - Co-localisation of *sox17:GFP* and *kdrl:mCherry* expression in the zebrafish heart. Lateral view of the heart region shown in Figure S4A. 48hpf embryos heterozygous for *sox17:GFP* and *kdrl:mCherry*. *kdrl:mCherry* marks the vascular endothelial lineage. Video depicting all Z slices from heart region shown in Figure S6A.

Supplementary Tables

Supplementary Table 13: Deletion and hybrid constructs. Depicts the amino acids removed or added to Sox32/Sox17 to generate constructs referred to within the manuscript.

pCS2+ Construct	Description
Sox17Δ7aa	Removal of amino acids 374-380 (EFEHCLS)
Sox32Δ7aa	Removal of amino acids 291-297 (EFDQYLN)
Sox17Δ7aa+Sox32 7aa	Replacement of Sox17 amino acids 374-380 (EFEHCLS) with Sox32 amino acids 291-297 (EFDQYLN)
Sox32Δ7aa+Sox17 7aa	Replacement of Sox32 amino acids 291-297 (EFDQYLN) with Sox17 amino acids 374-380 (EFEHCLS)
Sox17Δ7aa+Sox32 25aa	Replacement of Sox17 amino acids 374-380 (EFEHCLS) with Sox32 amino acids 373-397 (EFYLEQVRSDMLDQLDRSEFDQYLN)
Sox17ΔNTD	Removal of amino acids 1-55 (MSSPDAGYSSDDPSQTSSCSSVMMPGMGQCPW VDPLSPLSDSKSKHEKCSAAGPG)
Sox17ΔCTD35aa	Removal of amino acids 173-207 (GAGLPQYCENHTLFESYSLPTPDPSPMDAGTTEFF)
Sox17ΔCTD14aa	Removal of amino acids 366-379 (SGQMVDEVEFEHCL)
Sox17ΔCTD20aa	Removal of amino acids 394-413 (ISTVLSDASSAVYYCGYNNS)
Sox32ΔHMG	Removal of amino acids 67-149(ETRVRRPLNAFIIWTKEERRRLAQLNPDLENTD LSKILGKTWKAMSLADKRPYMQEAERLRIQHTIDYP NYKYRPRRRKCNKR)
Sox32ΔHMG+ Sox17 HMG	Replacement of Sox32 amino acids 67-149 (ETRVRRPLNAFIIWTKEERRRLAQLNPDLENTDLSK ILGKTWKAMSLADKRPYMQEAERLRIQHTIDYPNYK YRPRRRKCNKR) with Sox17 amino acids 60-142 (EPRIRRPMNAFMVWAKDERKRLAQQNPDLHNAEL SKMLGKSWKALPMVDKRPFVEEAERLRVKHMQDH PNYKYRPRRRKQVKR)

Supplementary Table 14: Primers used for PCR to generate deletion and hybrid constructs.

pCS2+ Construct	F Primer (5' → 3')	R Primer (5' → 3')
myc-Sox32	TCAGAAGAGGATCTGTATCTCGACCGGATGCTCCC	GATGAGTTTTTGTTCCATGCTGTTTTGCGTCCACT
myc-Sox32ΔNTD	CGAGTAAGACGCCCTTTAAA	CAGATCCTCTTCTGAGATGA
myc-Sox32ΔHMG	TGCAGCAAGATGCCTTCAAGTGAGAACGTCAGCTCTCCAAATGCCACCTTTGATC	CACGGGCGCTTTCGCTTCGGGACTTGAGCAGCTGGATTCAGACCCGACAGACAC
myc-Sox32ΔCTD23aa	GAATTTGACCAGTACCTCAATCC	CAAACACAGCACACACGCAC
myc-Sox32ΔCTD7aa	CCAGCACAGACTTTGGACCACAGC (Zhao , et al. 2013)	ACTGCGATCAAGCTGGTCCAAC (Zhao, et al. 2013)
Sox17Δ7 aa	TTTGGGGTCCCCAGTG	CACCTCGTCCACCATTTG
myc-Sox32Δ7aa + Sox17 7aa	CTGTCTCTCTCCAGCACAGACTTTGGAC	TGCTCAAACTCACTGCGATCAAGCTGGTC
Sox17Δ7aa + Sox32 7aa	AGTACCTCAATTTTGGGGTCCCCAGTG	GGTCAAATTCCACCTCGTCCACCATTTG
hSOX17	CCGGCTCGAGGCCACCATGAGCAGCCCGGATG	CTAGTCTAGATTACACGTCAGGATAGTTGCA
HiFi primers for Sox17 HMG	AAGCGAAAGCGCCCGTGGAGCCGCGCATCCGAAGG	GAAGGCATCTTGCTGCATCGTTTCACCTGTTTGCGACGs
HiFi primers for Sox17Δ7aa+Sox32 25aa gblock	CCATCGATTCGAATTCAAGGATGAGCAGTCCCGATG	AGTTCTAGAGGCTCGAGAGGTCAAGAATTATTATAGCCGC
Sox17ΔNTD	CGCGGAAAGAGCGAG	CATCCTTGAATTCGAATCGA
Sox17ΔCTD35aa	GCCCAGCTTCAGGATC	TGAATAACCAGCACTCATAC
Sox17ΔCTD14aa	TTTGGGGTCCCCAGTGC	AGAACTGTGGGTTGCTGTAG
Sox17ΔCTD20aa	CCTCTCGAGCCTCTAGAACT	TAAGTCAGACCCCGGCAAAG

Supplementary Table 15: crRNA sequences used to generate guide RNAs for RNP complex production.

	crRNA (5' → 3')
Dr.Cas9.SOX17.1.AF	TACACCTGACCCCTCT CCTA
Dr.Cas9.SOX17.1.AE	ACACGAGAAATGCTCT GCGG
Dr.Cas9.SOX17.1.AC	GGACAAACGTCCATTC GTTG
Alt-R® CRISPR-Cas9 Negative Control crRNA #1 (cat# 1072544)	CGTTAATCGCGTATAAT ACG
Alt-R® CRISPR-Cas9 Negative Control crRNA #2 (cat# 1072545)	CATATTGCGCGTATAGT CGC
Alt-R® CRISPR-Cas9 Negative Control crRNA #3 (cat# 1072546)8	GGCGCGTATAGTCGCG CGTA

Supplementary Table 16: qPCR primers.

	**F Primer (5'→3')**	**R Primer (5'→3')**
18S	TCGCTAGTTGGCATCGTTTA TG	CGGAGGTTCGAAGACGATCA
fabp2	GCCCATGACAACCTGAAGAT	TGTCCTTGCGTGTGAAAGTC
tbx15	GGTCAGCTTTGATAAACTCA AAC	CTGTTGGTTCTGGTAGGCT
mcamb	CTGCTATGCACAAGGCTACC	TTGGCAATGAGATCAGAGGTG
foxj1a	CAGATCCCACCTGGCAGAA CTC	ACTGGAGGTAATCTGCGCTTC
pltp	ACAACAGAGGAAACACTTG GACC	GGTCTCTGCCTTCAACATCTCC
vgll4l	CAGGACTGCTGCAATCACTC	GAAGGTTGGACTGCTTGGTG
ndr1	GCGAGCTGAACTTCGCATT	TCAATTAGCCCAACCGCAAG
sox17	CACAATGCGGAGCTGAGTAA	ATCGCTTGTTTCGTTTCACC
cxcr4a	TGGCTTATTACGAACACATC G	GAGCCGAATTCAGAGCTGTT

Supplementary Table 17: Species and Uniprot accession numbers of teleost and tetrapod Sox factor orthologues investigated for conservation in Supplementary Figures S11 and S19.

Species	Accession Number
Danio rerio Sox32	Q90Z46
Erpetoichthys calabaricus Sox32	A0A8C4RSN7
Carassius auratus Sox32	A0A6P6N386
Clupea harengus Sox32	A0A6P3VPI6
Esox lucius Sox32	A0A3P8ZP73
Gasterosteus aculeatus Sox32	A0AAQ4QAA5
Oryzias sinensis Sox32	A0A8C7YNR6
Oryzias latipes Sox32	H2M7I6
Anabas testudineus Sox32	A0A3Q1H6I0
Danio rerio Sox7	Q6TEN5
Danio rerio Sox18	E7F9I2
Danio rerio Sox17	Q5PQZ5
Xenopus laevis Sox17β.1	O42601
Homo sapiens SOX17	Q9H6I2
Gasterosteus aculeatus Sox17	G3NK71
Oryzias sinensis Sox17	A0A8C7YKM0
Oryzias latipes Sox17	C3VV17
Clupea harengus Sox17	A0A6P3VND2
Xenopus tropicalis Sox17α	Q8AWH3
Mus musculus SOX17	Q61473-1
Erpetoichthys calabaricus Sox17	A0A8C4RTG1
Carassius auratus Sox17	A0A6P6N1F7
Esox lucius Sox17	A0A3P8XTN7
Anabas testudineus Sox17	A0A3Q1H8J4

Amaral CRL, Pereira F, Silva DA, Amorim A, de Carvalho EF. 2018. The mitogenomic phylogeny of the Elasmobranchii (Chondrichthyes). Mitochondrial DNA A DNA Mapp Seq Anal 29:867-878.

Emms DM, Kelly S. 2019. OrthoFinder: phylogenetic orthology inference for comparative genomics. Genome Biol 20:238.

Morel B, Kozlov AM, Stamatakis A, Szollosi GJ. 2020. GeneRax: A Tool for Species-Tree-Aware Maximum Likelihood-Based Gene Family Tree Inference under Gene Duplication, Transfer, and Loss. Mol Biol Evol 37:2763-2774.

Near TJ, Thacker CE. 2024. Phylogenetic Classification of Living and Fossil Ray-Finned Fishes (Actinopterygii). Bulletin of the Peabody Museum of Natural History 65:3-302, 300.

Wagner DE, Weinreb C, Collins ZM, Briggs JA, Megason SG, Klein AM. 2018. Single-cell mapping of gene expression landscapes and lineage in the zebrafish embryo. Science 360:981 LP-987.

Zhao J, Lambert G, Meijer AH, Rosa FM. 2013. The transcription factor Vox represses endoderm development by interacting with Casanova and Pou2. Development 140:1090-1099.
