# Supplementary figures and images for: Molecular and Functional Divergence of Zebrafish Sox Paralogs Controlling Endoderm Formation and Left–Right Patterning
Source: Genome Biol Evol. 2025 Nov 11;17(11):evaf213. doi: 10.1093/gbe/evaf213 (PMC12648240; doi:10.1093/gbe/evaf213)

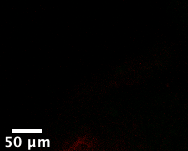

Supplement: evaf213_Supplementary_Data [file evaf213_supplementary_data.zip › Supplementary_Video_1.gif]
